# Supplementary material for: Integrating human behavior and snake ecology with agent-based models to predict snakebite in high risk landscapes
Source: PLoS Negl Trop Dis. 2021 Jan 22;15(1):e0009047. doi: 10.1371/journal.pntd.0009047 (PMC7857561; doi:10.1371/journal.pntd.0009047)
Supplement: S1 Fig — A. model outlines B. model structure. (DOCX) [file pntd.0009047.s001.docx]

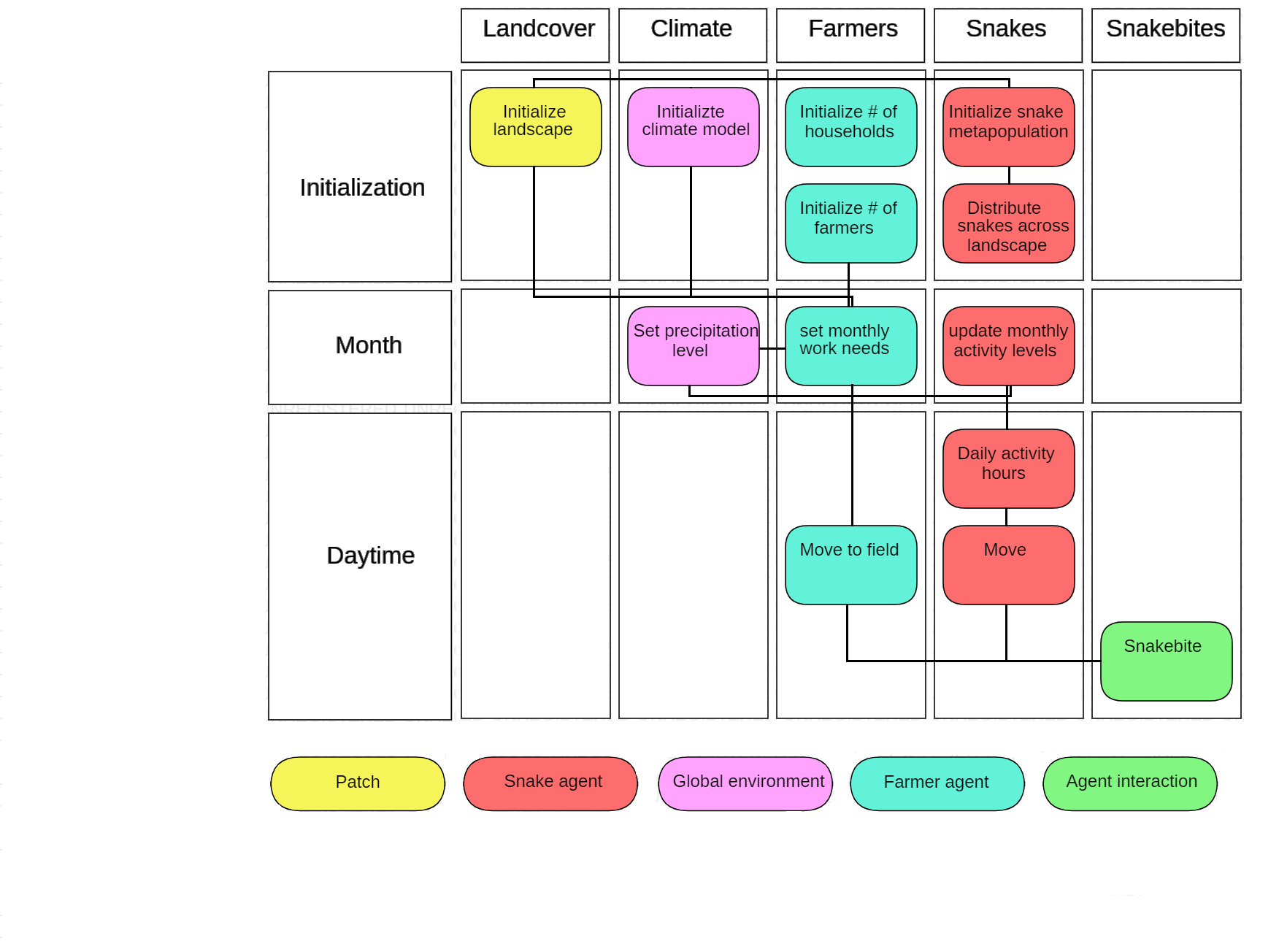


**Figure A:** **Model outline.** Columns represent the different subprocesses in the model, rows represent the different units of scheduling, and colours represent the different agents and inputs/outputs of the model. Lines between boxes represent the relationship between the agents and variables. Climate and landcover affect the agents’ behaviours across space and time, and this generates the spatio-temporal overlap between snake and humans that drive snakebite patterns.


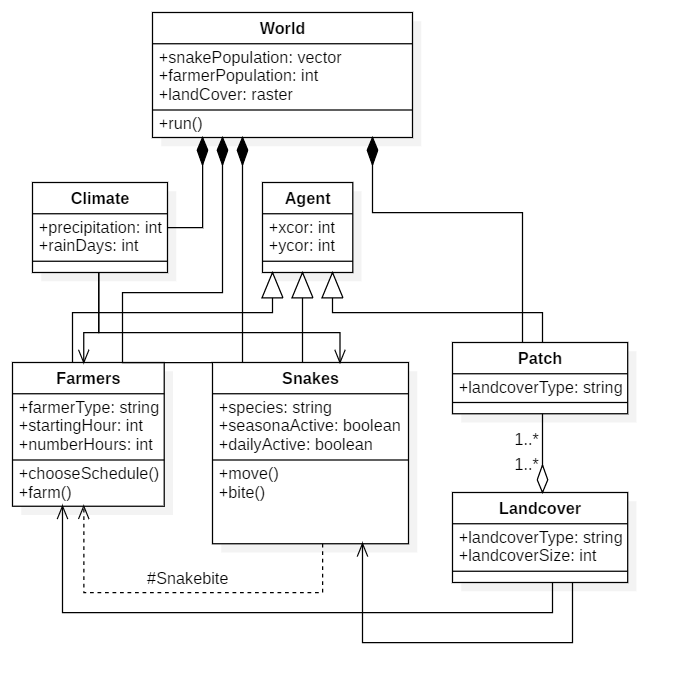


**Figure B:** **Model structure.** The model structure represented through UML class diagram. The attributes and operations of each class are simplified in order to represent the entire process and the relations between classes.
