# Supplementary material for: Integrating human behavior and snake ecology with agent-based models to predict snakebite in high risk landscapes
Source: PLoS Negl Trop Dis. 2021 Jan 22;15(1):e0009047. doi: 10.1371/journal.pntd.0009047 (PMC7857561; doi:10.1371/journal.pntd.0009047)
Supplement: S2 Fig — (DOCX) [file pntd.0009047.s002.docx]

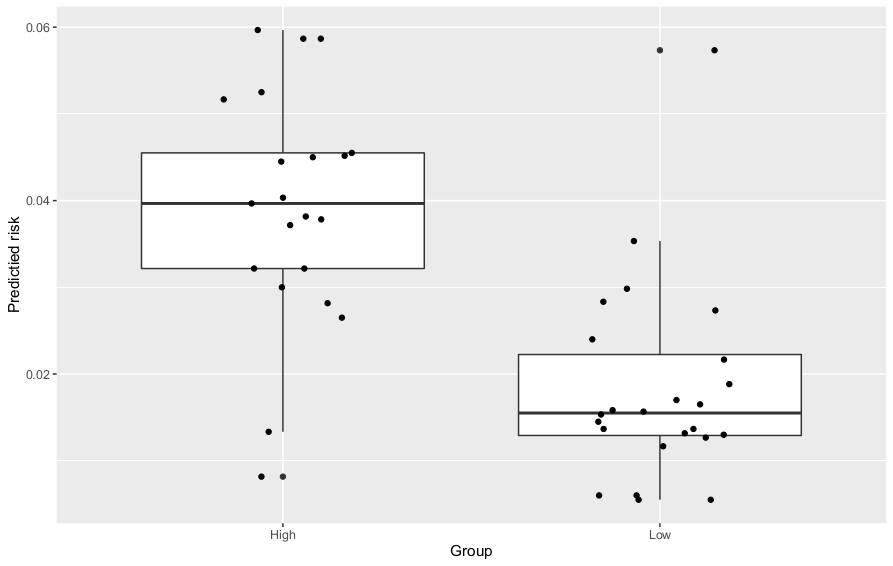


**Model output of mean snakebite risk for locations with high and low snakebite occurrence values estimated by Ediriweera et al 2016.** Results are based on 30 simulation runs for 45 different locations across the entire district of Ratnapura, with high and low defined as above or below the median snakebite risk for all locations.
