# Supplementary material for: Integrating human behavior and snake ecology with agent-based models to predict snakebite in high risk landscapes
Source: PLoS Negl Trop Dis. 2021 Jan 22;15(1):e0009047. doi: 10.1371/journal.pntd.0009047 (PMC7857561; doi:10.1371/journal.pntd.0009047)
Supplement: S3 Fig — A. Rice farmers B. Tea farmers C. Rubber farmers. (DOCX) [file pntd.0009047.s003.docx]

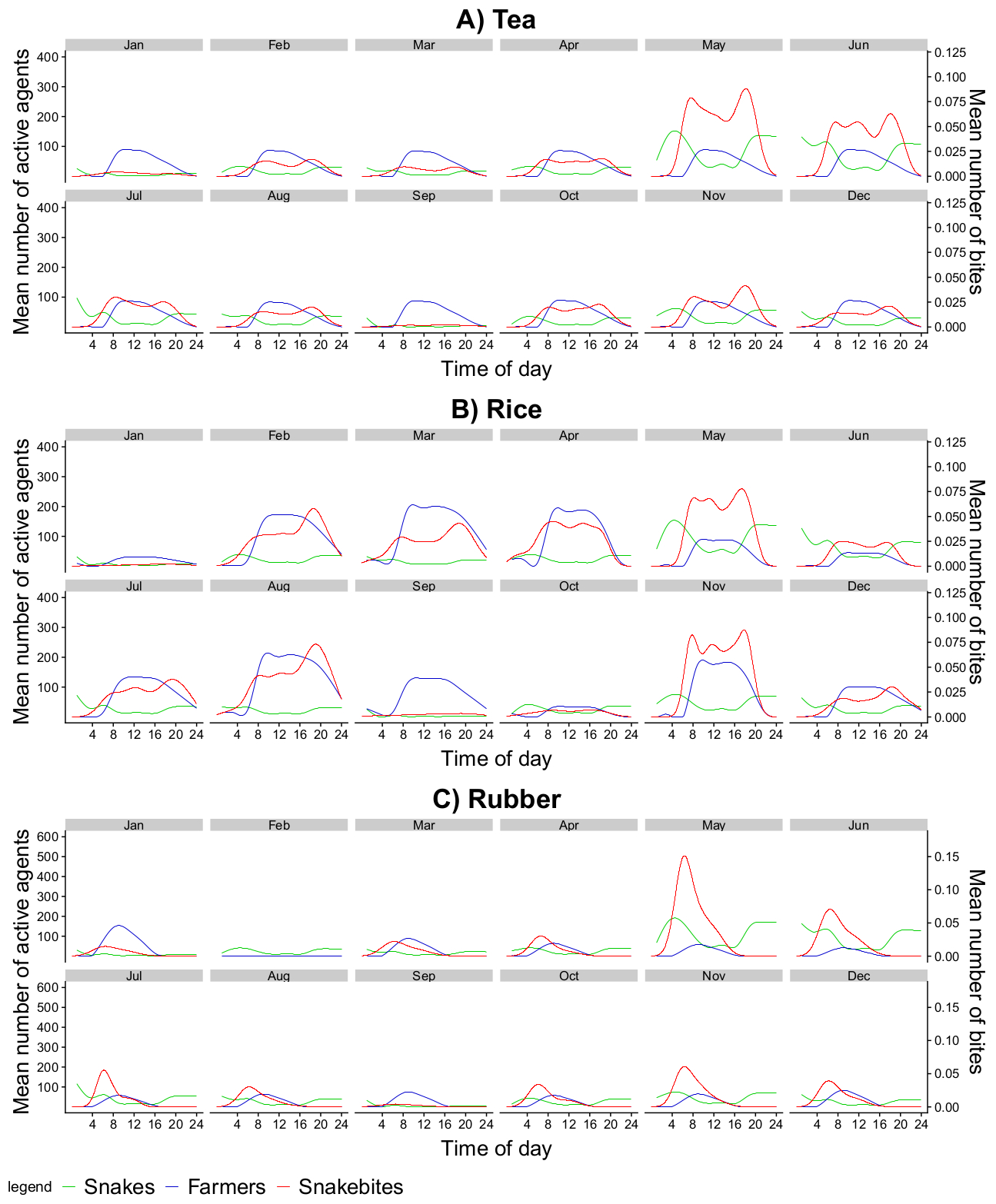


**The daily spatial temporal overlap of farmers and snakes.** The values are an average of 660 simulation runs. Each graph follows the daily spatio-temporal overlap between farmers and snakes that cause the emergence of snakebites patterns for **A)** tea **B)** rice, and **C)** rubber.
