# Supplementary material for: Integrating human behavior and snake ecology with agent-based models to predict snakebite in high risk landscapes
Source: PLoS Negl Trop Dis. 2021 Jan 22;15(1):e0009047. doi: 10.1371/journal.pntd.0009047 (PMC7857561; doi:10.1371/journal.pntd.0009047)
Supplement: S7 Fig — (DOCX) [file pntd.0009047.s007.docx]

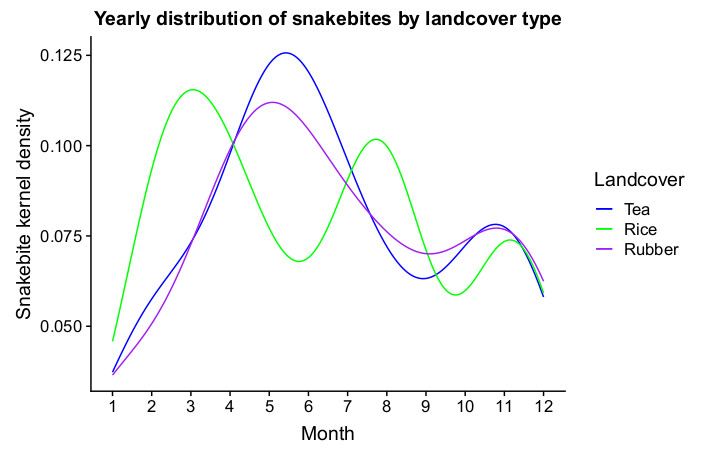


**A secondary predictions monthly distribution of bites by landcover.** Our model shows that the different landcovers are going to have different patterns of snakebites distribution across the year.
