# Supplementary material for: Integrating human behavior and snake ecology with agent-based models to predict snakebite in high risk landscapes
Source: PLoS Negl Trop Dis. 2021 Jan 22;15(1):e0009047. doi: 10.1371/journal.pntd.0009047 (PMC7857561; doi:10.1371/journal.pntd.0009047)
Supplement: S1 Table — A. Support vector machine B. Maximum likelihood. (DOCX) [file pntd.0009047.s009.docx]

**Table A**: accuracy assessment for support vector machine classifications

Overall Accuracy = (53684/64481) 83.2555%

Kappa Coefficient = 0.6847

| Class | Commission | Omission | Commission | Omission |
| --- | --- | --- | --- | --- |
|  | (Percent) | (Percent) | (Pixels) | (Pixels) |
| Rubber | 43.58 | 87.05 | 659/1512 | 5732/6585 |
| Water | 0.59 | 0.00 | 43/7265 | 0/7222 |
| Forest | 16.43 | 3.68 | 7556/45990 | 1469/39903 |
| Rice | 13.39 | 29.14 | 654/4885 | 1740/5971 |
| Tea | 39.03 | 38.67 | 1885/4829 | 1856/4800 |

| Class | Prod. Acc. | User Acc. | Prod. Acc. | User Acc. |
| --- | --- | --- | --- | --- |
|  | (Percent) | (Percent) | (Pixels) | (Pixels) |
| Rubber | 12.95 | 56.42 | 853/6585 | 853/1512 |
| Water | 100.00 | 99.41 | 7222/7222 | 7222/7265 |
| Forest | 96.32 | 83.57 | 38434/39903 | 38434/45990 |
| Rice | 70.86 | 86.61 | 4231/5971 | 4231/4885 |
| Tea | 61.33 | 60.97 | 2944/4800 | 2944/4829 |

**Table B**: accuracy assessment for maximum likelihood classifications

Overall Accuracy = (52041/64481) 80.7075%

Kappa Coefficient = 0.6664

| Class | Commission | Omission | Commission | Omission |
| --- | --- | --- | --- | --- |
|  | (Percent) | (Percent) | (Pixels) | (Pixels) |
| Rubber | 57.38 | 81.97 | 1598/2785 | 5398/6585 |
| Water | 0.12 | 0.00 | 9/7231 | 0/7222 |
| Forest | 11.86 | 10.88 | 4783/40344 | 4342/39903 |
| Rice | 14.34 | 33.96 | 660/4603 | 2028/5971 |
| Tea | 56.63 | 14.00 | 5390/9518 | 672/4800 |

| Class | Prod. Acc. | User Acc. | Prod. Acc. | User Acc. |
| --- | --- | --- | --- | --- |
|  | (Percent) | (Percent) | (Pixels) | (Pixels) |
| Rubber | 18.03 | 42.62 | 1187/6585 | 1187/2785 |
| Water | 100.00 | 99.88 | 7222/7222 | 7222/7231 |
| Forest | 89.12 | 88.14 | 35561/39903 | 35561/40344 |
| Rice | 66.04 | 85.66 | 3943/5971 | 3943/4603 |
| Tea | 86.00 | 43.37 | 4128/4800 | 4128/9518 |
