# Supplementary material for: Integrating human behavior and snake ecology with agent-based models to predict snakebite in high risk landscapes
Source: PLoS Negl Trop Dis. 2021 Jan 22;15(1):e0009047. doi: 10.1371/journal.pntd.0009047 (PMC7857561; doi:10.1371/journal.pntd.0009047)
Supplement: S2 Table — (DOCX) [file pntd.0009047.s010.docx]

| Specie | Forest | Rubber | Rice | Home | Tea |
| --- | --- | --- | --- | --- | --- |
| *Hypnale hypnale* | 0.929 | 0.976 | 0.348 | 2.472 | 1.387 |
| *Naja naja* | .653 | .895 | 1.352 | 5.209 | .387 |
| *Daboia russelii* | 0.573 | 0.953 | 1.440 | 5.599 | 0 |
| *Bungarus ceylonicus* | 0.825 | 1.087 | 0 | 24.92 | 1.27 |
| *Bungarus caeruleus* | 0.722 | 1.012 | 1.289 | 4.277 | 0 |
| *Trimeresurus trigonocephalus* | 1.051 | 0.947 | 0.235 | 2.269 | 0.777 |
| *Echis carinatus* | 0.391 | 0.408 | 2.429 | 4.585 | 0 |

The land association factor between different snake species and landcover types.
