# Supplementary material for: Integrating human behavior and snake ecology with agent-based models to predict snakebite in high risk landscapes
Source: PLoS Negl Trop Dis. 2021 Jan 22;15(1):e0009047. doi: 10.1371/journal.pntd.0009047 (PMC7857561; doi:10.1371/journal.pntd.0009047)
Supplement: S3 Table — (DOCX) [file pntd.0009047.s011.docx]

| Hours | Rice | Tea | Rubber |
| --- | --- | --- | --- |
| 3 | 0 | 0 | 1 |
| 4 | 1 | 1 | 0 |
| 5 | 1 | 1 | 2 |
| 6 | 0 | 3 | 2 |
| 7 | 1 | 2 | 3 |
| 8 | 1 | 8 | 0 |
| 9 | 5 | 2 | 0 |
| 10 | 1 | 3 | 1 |
| 11 | 5 | 1 | 0 |
| 12 | 2 | 0 | 0 |
| 13 | 1 | 1 | 0 |
| 14 | 0 | 0 | 0 |
| 15 | 0 | 0 | 0 |
| 16 | 0 | 0 | 0 |
| 17 | 1 | 0 | 0 |
| 18 | 1 | 0 | 0 |

The table represents the different possible number of hours worked on the different landcover types as reported by farmers during our field work.
