# Supplementary material for: Integrating human behavior and snake ecology with agent-based models to predict snakebite in high risk landscapes
Source: PLoS Negl Trop Dis. 2021 Jan 22;15(1):e0009047. doi: 10.1371/journal.pntd.0009047 (PMC7857561; doi:10.1371/journal.pntd.0009047)
Supplement: S1 Appendix — A. Model selection B. Sensitivity analysis C. Results. (DOCX) [file pntd.0009047.s014.docx]

# Appendix – Technical Evaluation

## A. Model selection:

For model selection we removed models that gave unrealistic representations of the system we are modeling, including non-realistic proportions of snakebites between species and unlikely months for snakebite peaks. If a certain mathematical formulation of our model caused the loss of a pattern that was previously observed on the macro level, then it was not used for later analysis.

We checked the structure of the model by comparing different relationships between variables and outcomes. More specifically, we checked for the possible relationship between the aggressiveness index and the propensity of snakes to bite, and the different possible relationships between precipitation and snake activity. This allowed us to make sure our modeling method was sensible before checking for the sensitivity of our model to changes in variable magnitude. The different relationships were observed through the yearly distribution of bites, the daily distribution of bites, the attack assemblage of species causing the bites, and the total number of bites.

1. The aggressiveness index was collected as ordinal data, a ranking of the propensity to bite of the different snake species. For this index, in addition to the linear relationship between the aggressiveness index and the propensity to bite as described above, we checked two additional mathematical relationships: a concave relationship represented by $\sqrt{aggressiveness}$, where snakes with a low score would be more affected by the index while snake species with high scores would show similar behavior; and a convex relationship represented by (aggressiveness)^2^, where snakes with a high score would be more affected by the index and snakes with low scores would show more similar behavior.
2. We also checked three different relationships between precipitation and snake activity, a linear relationship as described above, where snake activity is directly proportional to precipitation; a concave (decelerating) relationship represented by $\sqrt{\frac{precipitation}{{precipitation}_{max}}}$, and a convex (accelerating) relationship that was represented by (precipitation/precipitation_max_)^2^.

## B. Sensitivity analysis

We then conducted a sensitivity analysis against four different variables for which we had insufficient data or no data at all. Our sensitivity analysis helped identify which variables were the most influential on the simulation output. During the sensitivity analysis we used a linear relationship between the aggressiveness index and the propensity to bite for the sake of simplicity and saving computation time.

The variables used for the sensitivity analysis were:

1. After the model selection step, we chose to conduct additional sensitivity analysis for both a linear relationship and a concave relationship between precipitation and snake seasonal activity, but not for a convex relationship because it produced unrealistic patterns in the model selection step. For the two possibilities we checked the effects of different strengths of association between the precipitation and activity as:
   1. (precipitation / precipitation_max_)^x^ with x = 0.1, 0.25, 0.5, for the concave relationship.
   2. (x + precipitation)/(x + precipitation_max_), with x = 100, 500, 900 For the linear relationship.
2. We originally defined the baseline dial activity levels of snakes with a probability of being active at p = 0.1. For the sensitivity analysis we checked a baseline probability of activity with a value of p = 0, 0.2 and 0.3 for all species except for cathemeral snakes, which were kept at a probability of 0.1 across all times of day.
3. The labour index value was collected during the field work and represented the expected number of people working in a 1 km^2^ area for each landcover. This index was used as an input for the algorithm that decides how farmers allocated their time to different land cover types according to seasonal needs. For the sensitivity analysis we checked the lowest and the highest value of the index.
4. Since our snake population size was calibrated using previous research, for the sensitivity analysis we checked for different population sizes. We changed the factor that was used for scaling up the PPM models by values that ranged between 1x10^10^ and 9x10^10^ .

The results of the sensitivity analyses were monitored with several different model outputs: frequency of bites in different landcovers, frequency of bites across snake species, daily distribution of bites, monthly distribution of bites, number of bites per location, and total number of bites per simulation run.

## C. Results

The most-probable relationships between the aggressiveness index and the propensity of snakes to bite were convex and linear, based on the aggressiveness index (see Figures C.1-4). The most probable relationships between snake seasonal activity and rainfall were linear and concave (decelerating) (see Figures C.5-8).

In the sensitivity analysis, for the precipitation signal strength we found a threshold value with which a yearly pattern of snakebites is transformed (see Figures C.10, C.14). For population size a significant threshold was observed when the PPM models were scaled up by a value of 1x10^10^ under which our model no longer showed any significant pattern (see Figures C.26). For the labour index and the baseline activity, we kept the values as they were originally defined (see Figure C.17-24), since we did not find any significant thresholds.


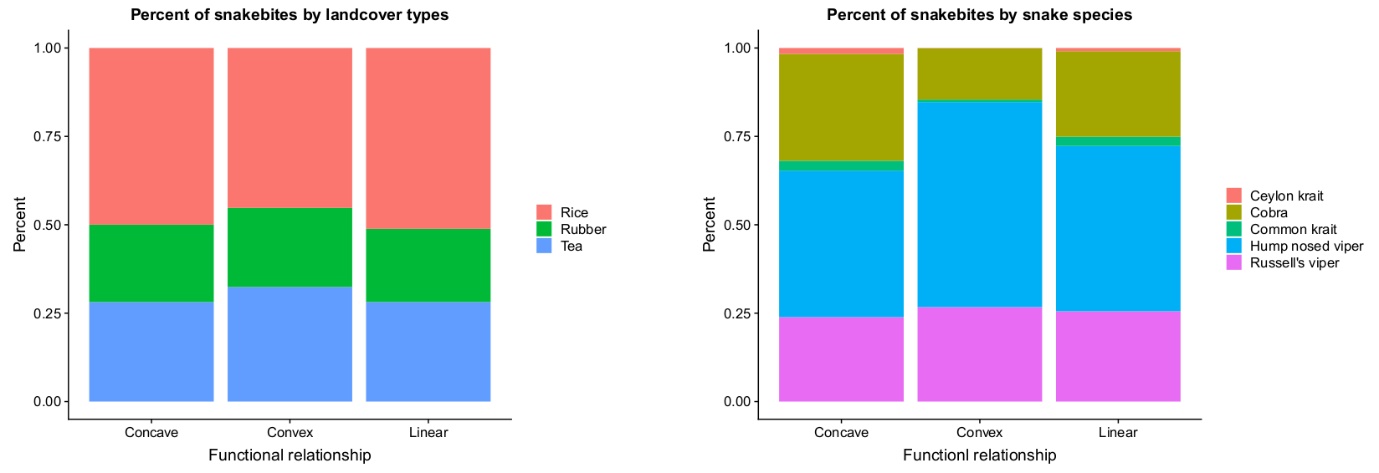
Figure 1: Our simulation was executed 30 times for each functional relationship at each one of the locations modelled. Changing the functional relationships for the aggressiveness index and propensity to bite had only a small effect on the percentage of snakebites occurring on each landcover type, but had a large effect on the percentage of bites caused by each one of the species, with a convex functional relationship showing an increased number of Hump nosed viper bites, and a decreased number of Cobra bites.


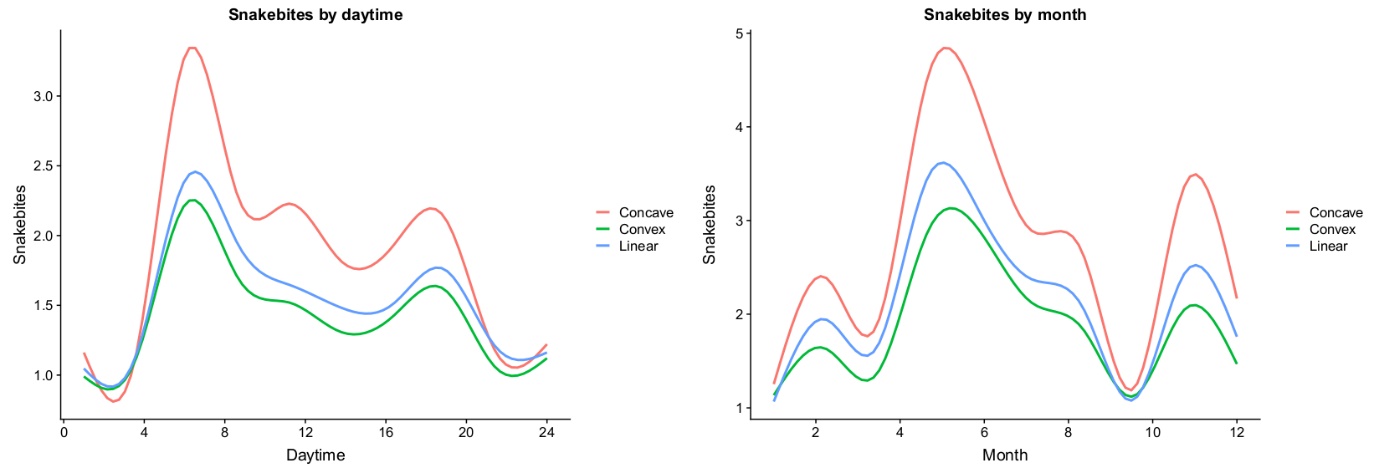


Figure 2: Changing the functional relationships between the aggressiveness index and the propensity to bite had an effect on the daily distribution of snakebites, with the early morning snakebite peak becoming much large when a concave relationship was defined. For the yearly distribution of snakebites there was change in magnitude but not in pattern.


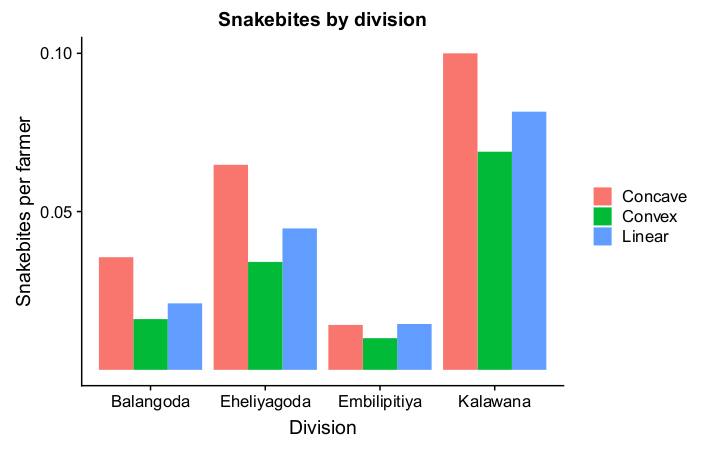


Figure 3: Changing the functional relationships between the aggressiveness index and propensity to bite had a only a moderate effect on the geographical patterns of snakebites, where each one of the divisions showed similar patterns when the functional relationship changed.


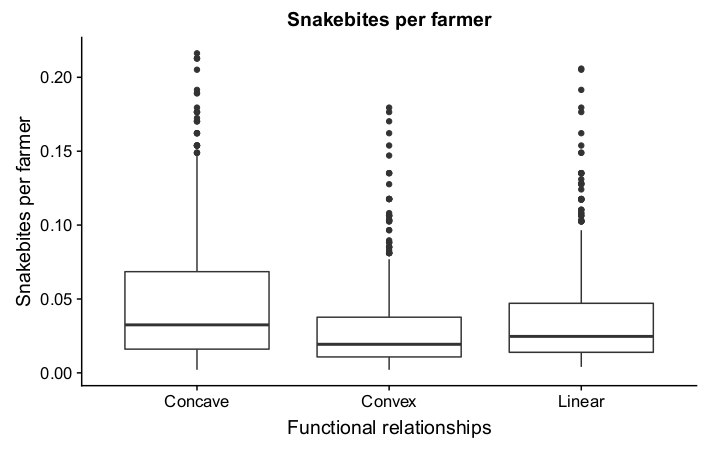


Figure 4: Changing the functional relationships had some effect on the total number of bites, with the mean number of snakebites remaining similar, but for the convex relationships there was a lower mean and less variance in comparison with the linear, and concave.


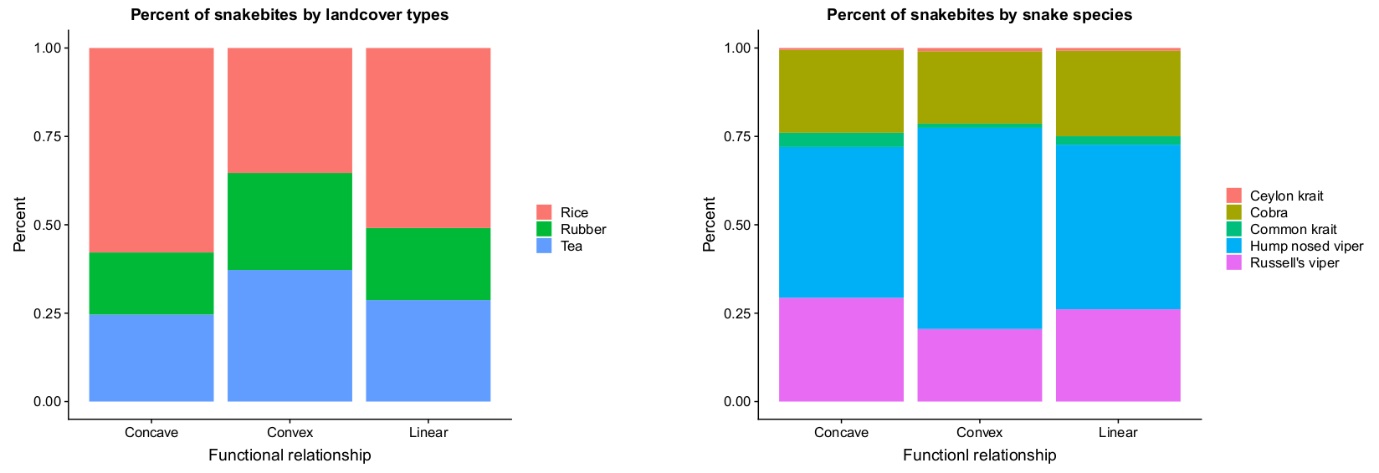


Figure 5: Our simulation was executed 30 times for each functional relationship at each one of the locations modelled. Changing the functional relationships influenced the percentage of snakebites occurring on each landcover type with the convex relationship causing less snakebites on rice paddies and more snakebites on rubber plantations. There was also an effect on the percentage of bites caused by each one of the species, with a convex functional relationship showing an increased number of Hump nosed viper bites, and fewer Russell’s viper bites.


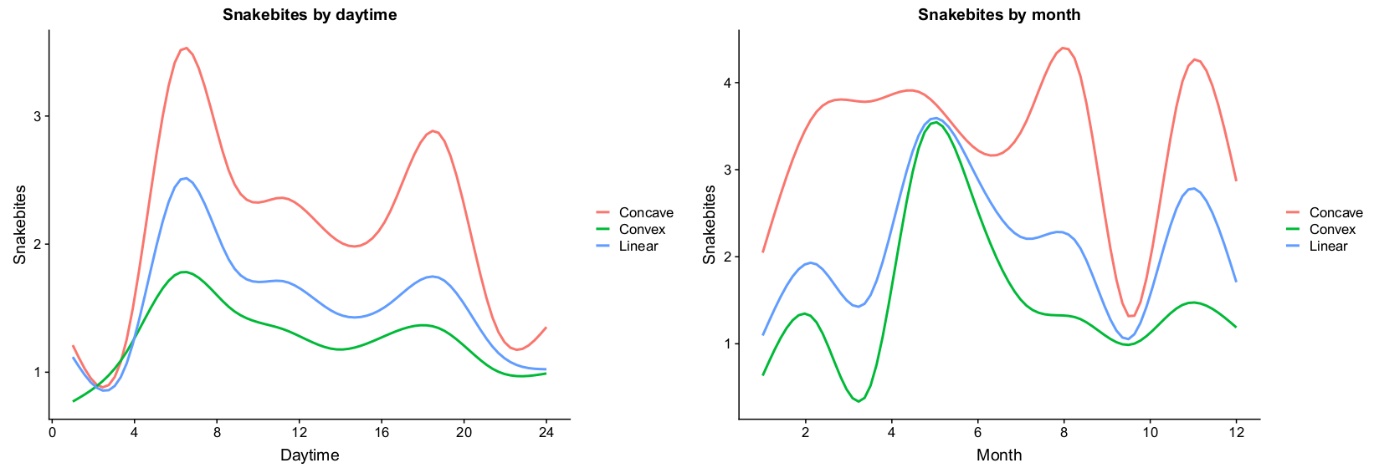


Figure 6: Changing the functional relationships influenced the distribution of bites both on the daily level and on the monthly level. On the daily level there was only a change in magnitude, with the concave relationship showing a higher number of snakebites through the different hours of the day. On the monthly level there was also a change in pattern. While the linear and convex relationships showed similar patterns across the year, the concave relationship showed a different patter, with a much larger peak between February and May, and a second large snakebite peak at the month of August.


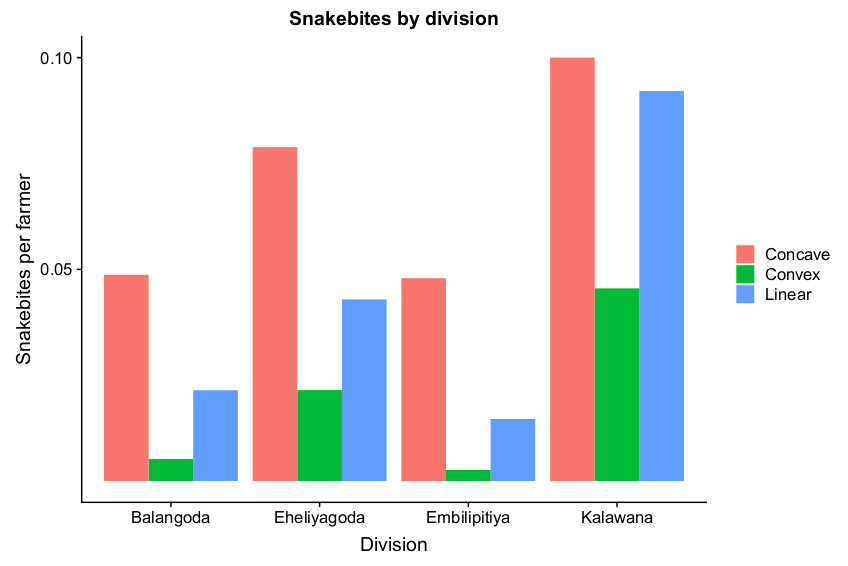


Figure 7: Changing the functional relationships between precipitation and snake seasonal activity levels influenced the different locations modelled at the different divisions in different ways, meaning that the different functional relationships had an effect on the geographical distribution of snakebites.


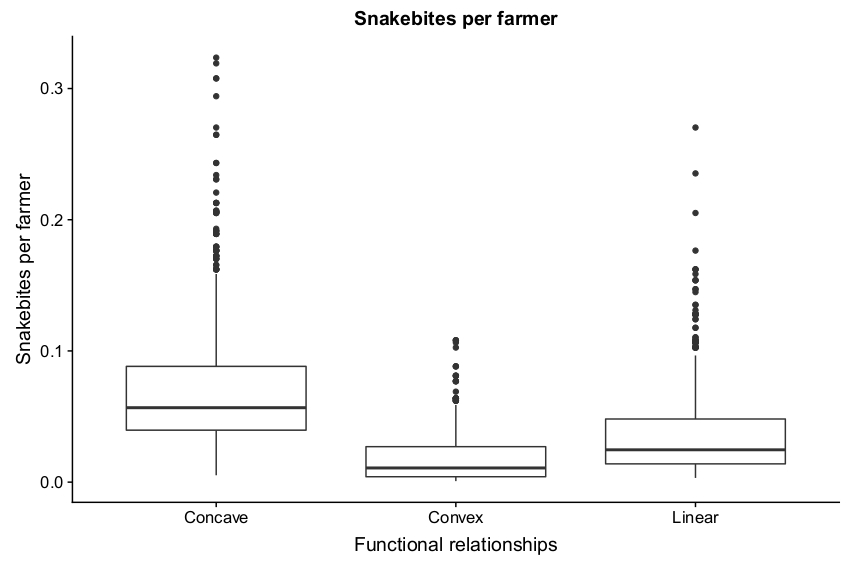


Figure 8: Changing the functional relationships between precipitation and the snake seasonal activity influenced the total number of snakebites. The convex relationship showed a smaller mean of snakebites across different simulations, and less variation in the number of snakebites as well. The concave relationship showed a high mean than the other two, and much more uncertainty in model outcomes.


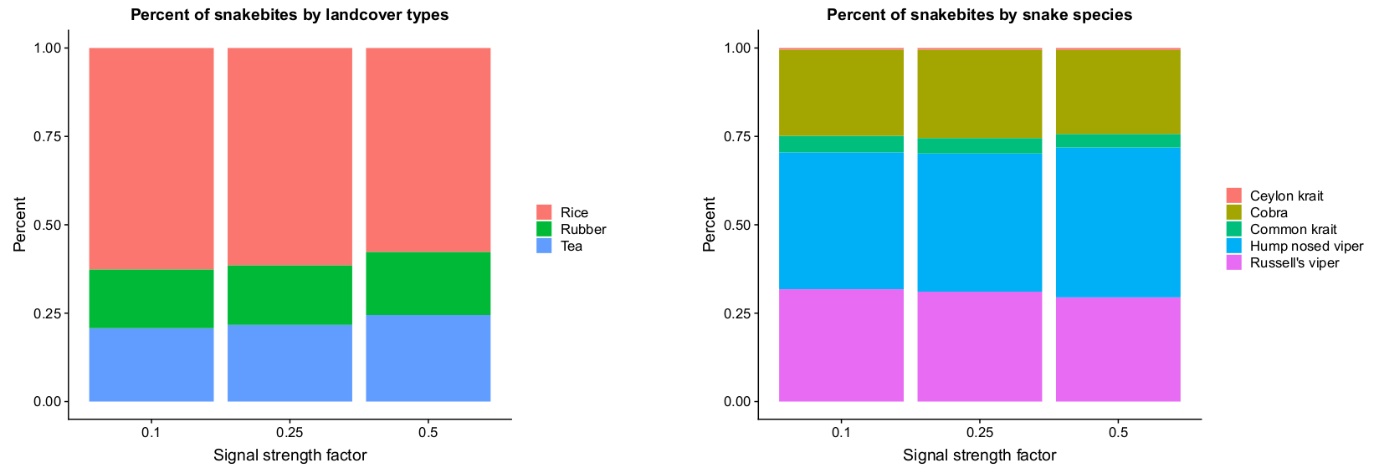


Figure 9: Our simulation was executed 30 times for each relationship intensity at each one of the locations modelled according to the following posibilites: (precipitation / precipitation_max_)^x^ with x = 0.1, 0.25, 0.5,. The different signal strengths between a concave precipitation function precipitation and snake seasonal activity had only a small effect on the percentage of snakebites occurring on each landcover type, and the percentage of snakebites caused by each one of the snake species.


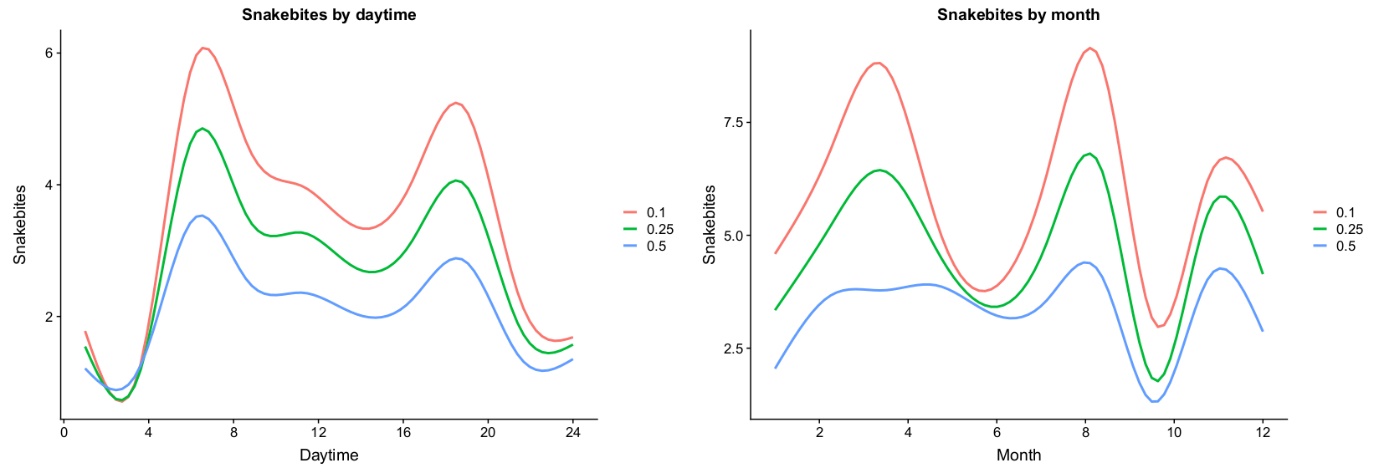


Figure 10: The different relationships intensities between a concave precipitation function and snake seasonal activity had a strong effect on the temporal distribution of snakebites. On the daytime level the differences only amounted to change in magnitude as the signal was strengthened. On the monthly level we observed change in pattern as well, when after a certain reduction in signal strength we lose some of the distinct yearly snakebite patterns such as a snakebite peak between March and May.


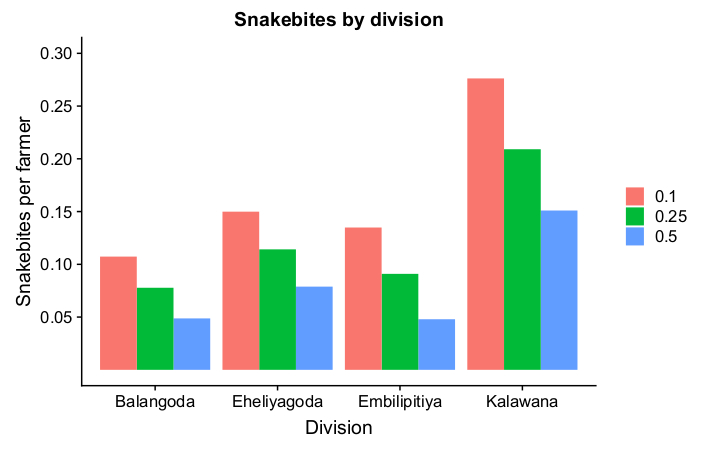


Figure 11: The different relationship intensities between a concave precipitation function and snake seasonal activity tended to effect different regions in similar ways. An increase in intensity of signal had the same effect regarding number of snakebites across all locations that we modelled.


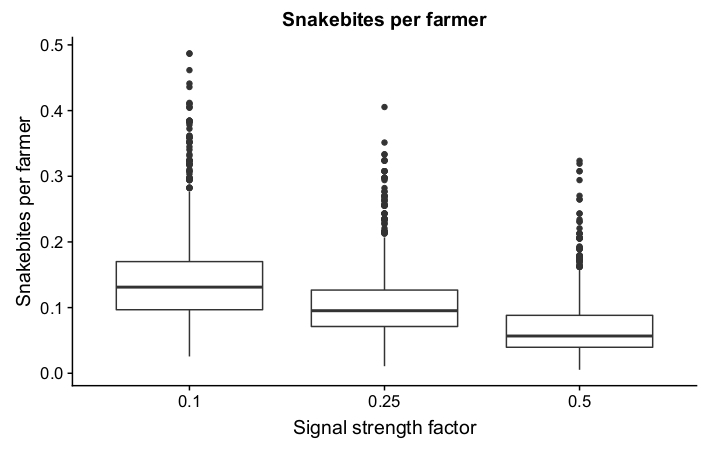


Figure 12: The different relationship intensities between a concave precipitation function and snake seasonal activity showed that an increase in intensity causes an increase in total number of snakebites. The distribution of snakebites around the mean remained relatively similar regardless of the intensities.


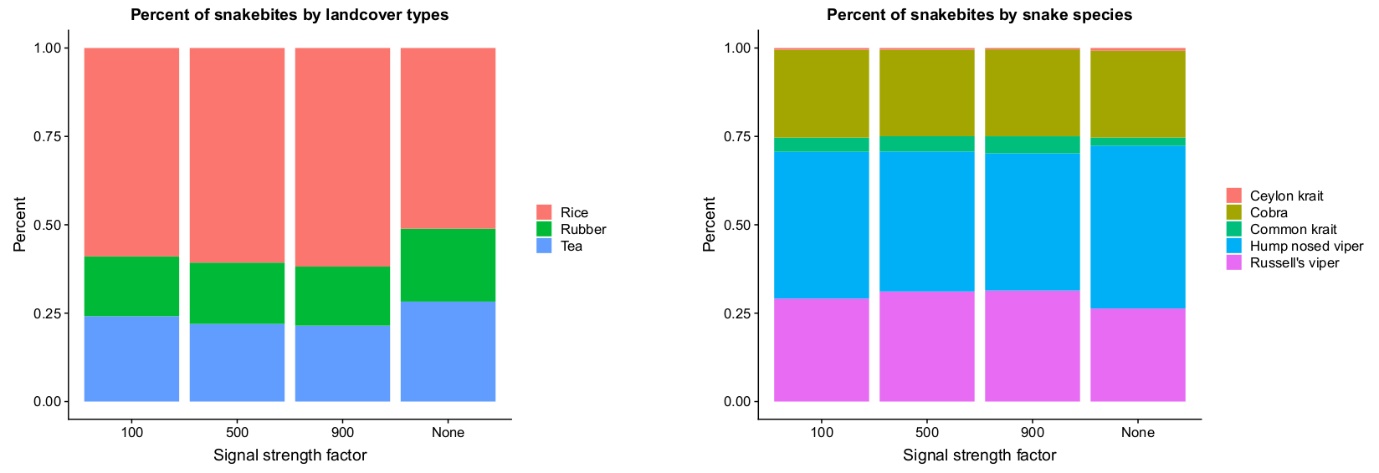


Figure 13: Our simulation was executed 30 times for each signal strength at each one of the locations modelled according to the following possibilities: (x + precipitation)/(x + precipitation_max_), with x = 100, 500, 900. Changing the signal strength had only a small effect on the percentage of snakebites occurring in each landcover type, and only minor effects on the percentage of bites caused by each on the of the snake species.


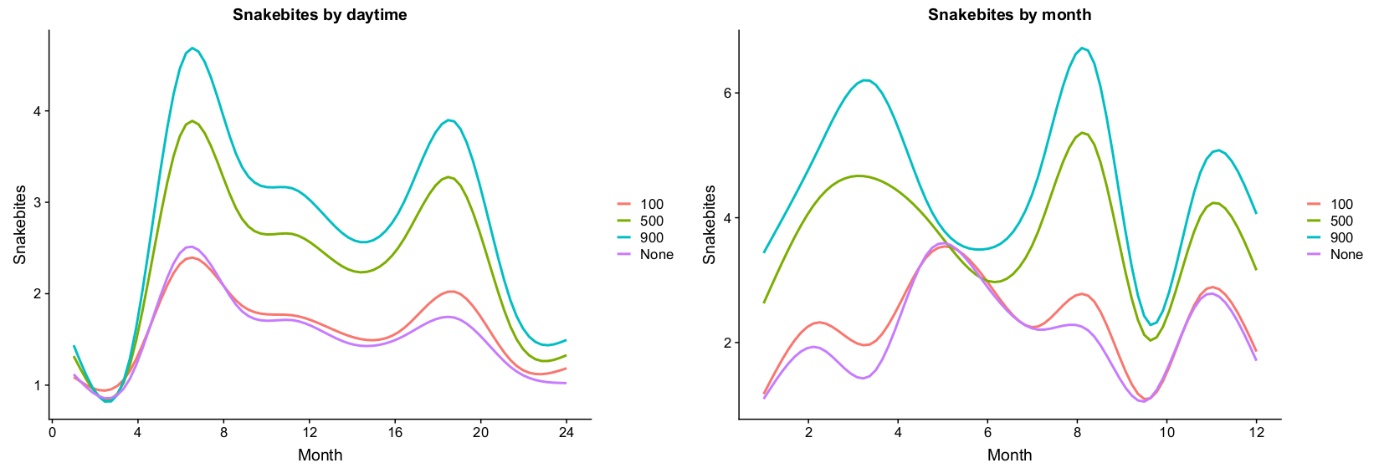


Figure 14: Changing the signal strength had an effect both on the daily distribution of snakebites and on the monthly distribution of snakebites. With a weak and medium signal, the snakes were mostly active regardless of precipitation, so snakebite patterns tended to follow the working patterns of the farmers, while with the strong and linear signals snakes were only active when precipitation was high, and in these two cases snakebite patterns tended to be more influenced by snake activity.


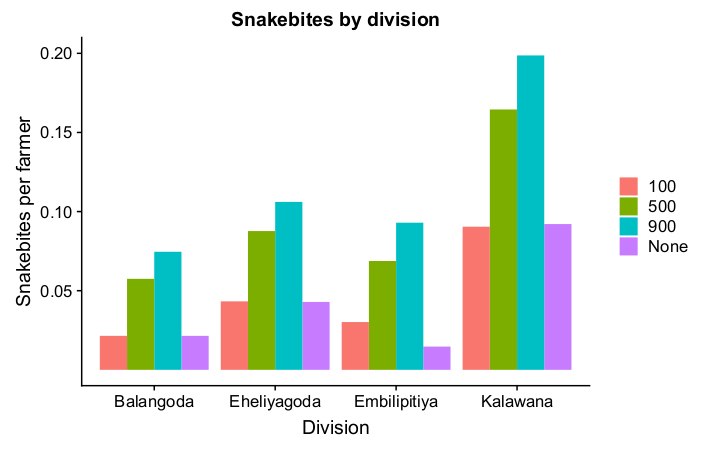


Figure 15: changing the signal strength tended to effect different regions in similar ways across different divisions.


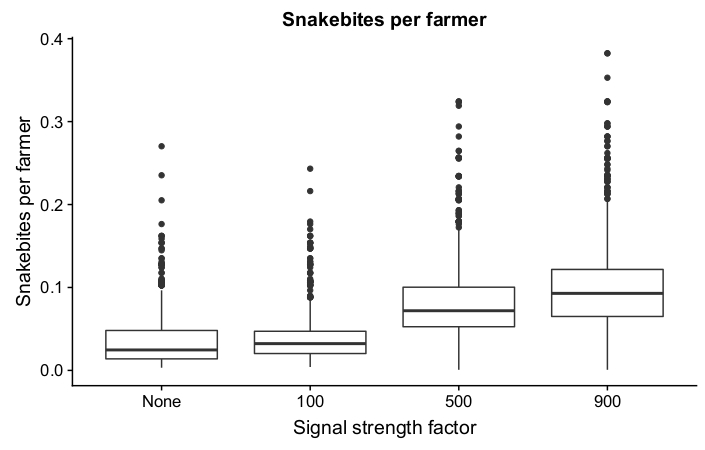


Figure 16: Changing the signal strength factor influenced the total number of snakebites both on the mean and the variance of snakebites per farmer.


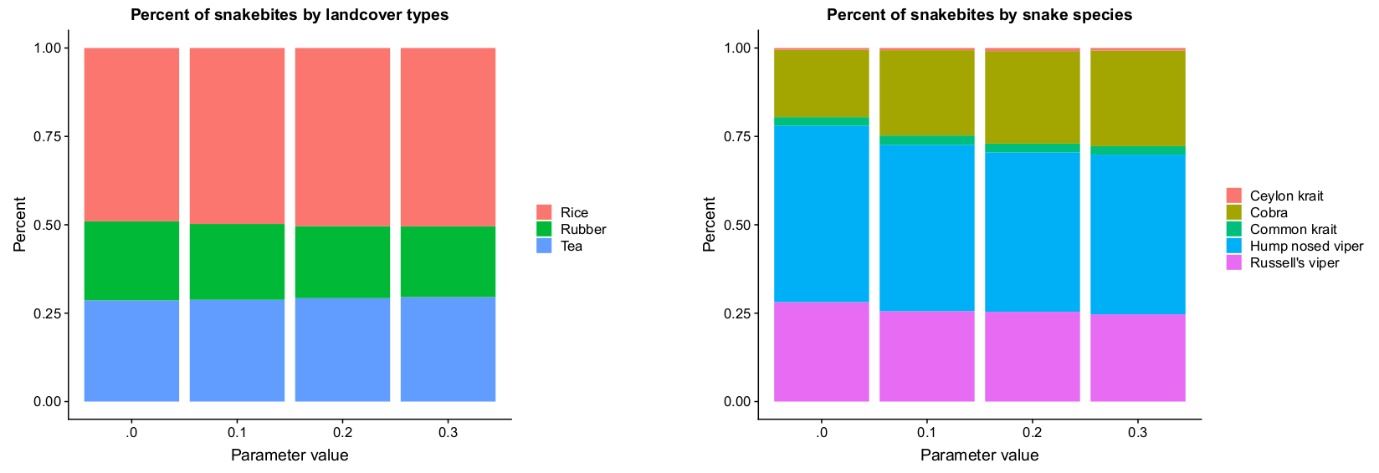


Figure 17: Our simulation was executed 30 times for each activity baseline at each one of the locations modelled with the following possibilities for baseline activity: p = 0, 0.1, 0.2 and 0.3 for all species except for cathemeral snakes, which were kept at a probability of 0.1 across all times of day. Changing the baseline activity probability had only a small effect on the percentage of snakebites on each landcover type but had some effect on the percentage of bites caused by each one of the snake species. A lower baseline probability tended to result in a higher proportion of Russell’s viper bites, while a higher baseline level increased the proportion of bites caused by Cobras.


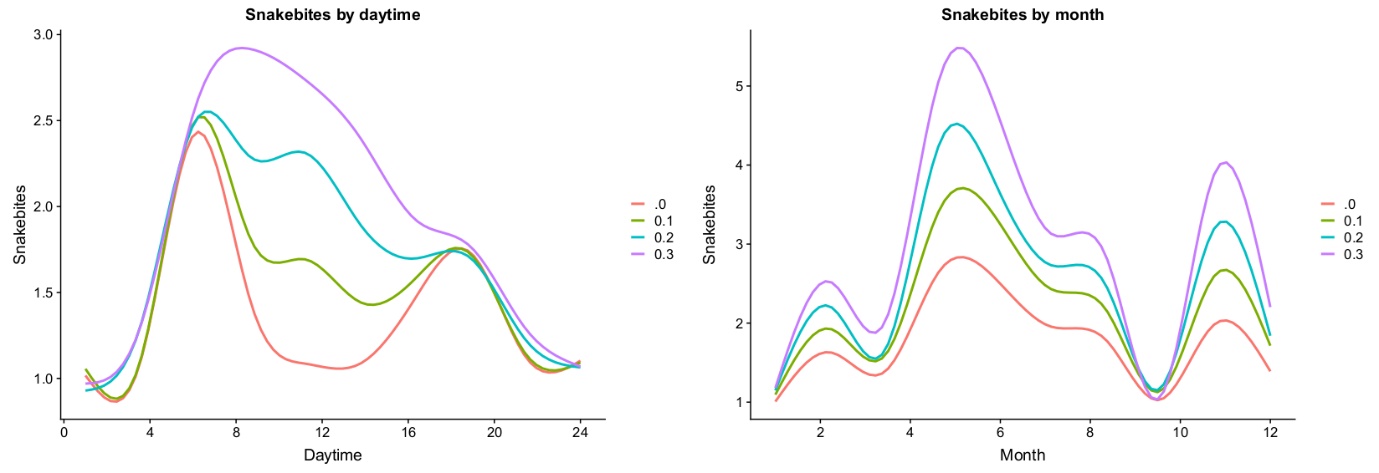


Figure 18: Changing the baseline activity probability had a very strong effect on the distribution of snakebites across the day. A high baseline probability caused a shift of snakebite peak later into the day. Lower baseline activity levels tended to generate a bimodal peak pattern with one large peak in the morning and a second large peak at late afternoon. Changing baseline activity caused a change in magnitude for the yearly distribution of snakebites, but not in pattern.


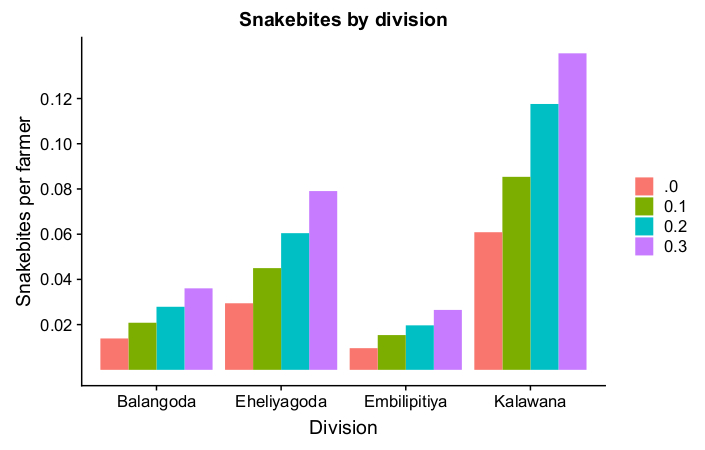


Figure 19: Different baseline probabilities tended to effect different divisions in similar ways. An increase in baseline probability had the same effect regarding the relative number of snakebites across all divisions that we modelled.


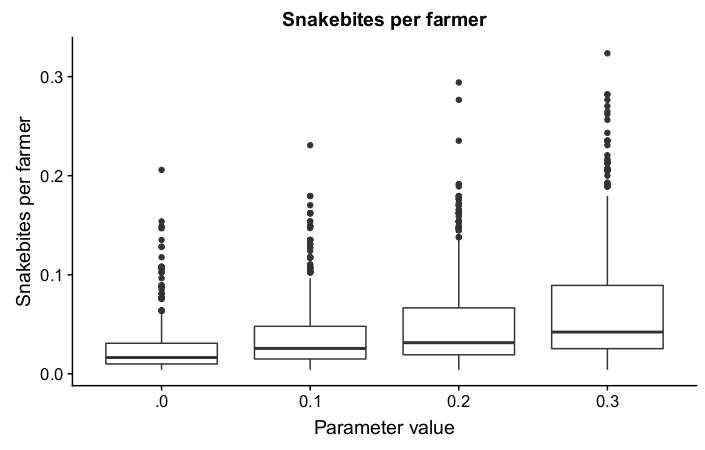


Figure 20: The different baseline probabilities showed that an increase in value would cause an increase in total number of snakebites. The distribution of snakebites around the mean also changed, with a larger variance for higher baseline probability levels, meaning that the uncertainty levels were increase as well.


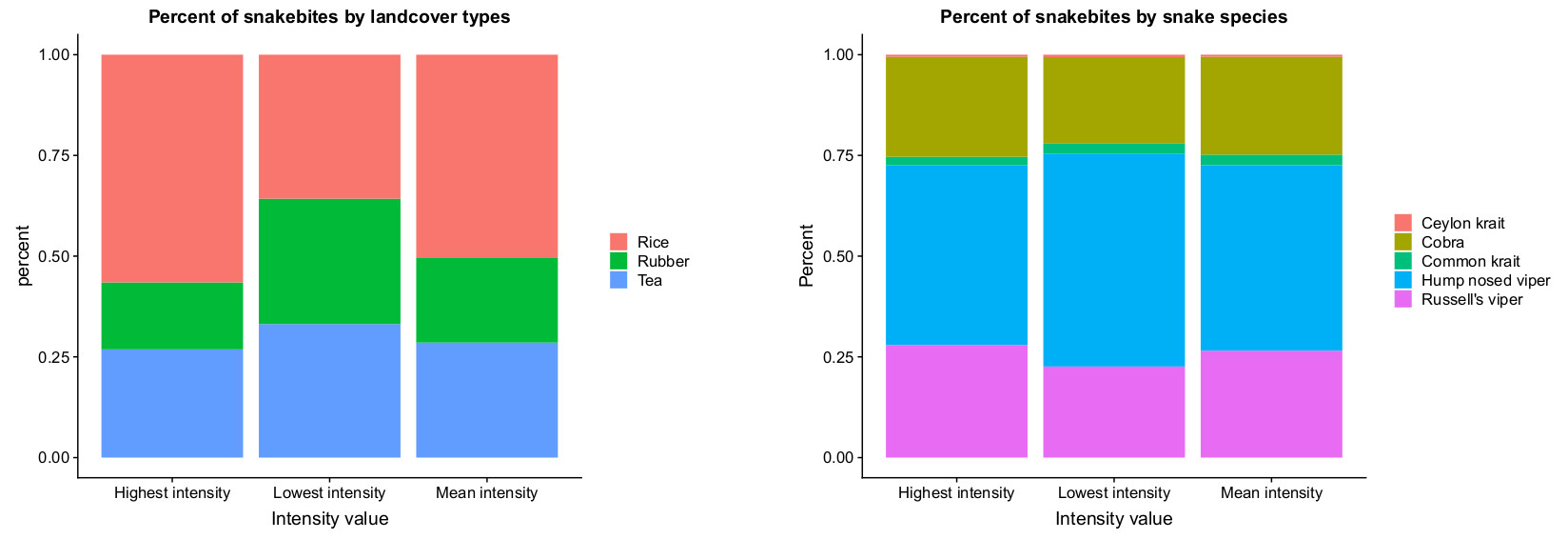


Figure 21: Our simulation was executed 30 times for each intensity value at each one of the locations modelled, where we checked the lowest and highest index values in addition to the mean value. Changing the index influenced the percentage of bites that occurred on the different landcover types. The higher index values that were used caused more snakebites on the rice landcover. Rice had the largest difference between the lowest index value and the highest index values, and this was most likely driving the variation in output. The species of snakes causing snakebites only showed a moderate variation between index values.


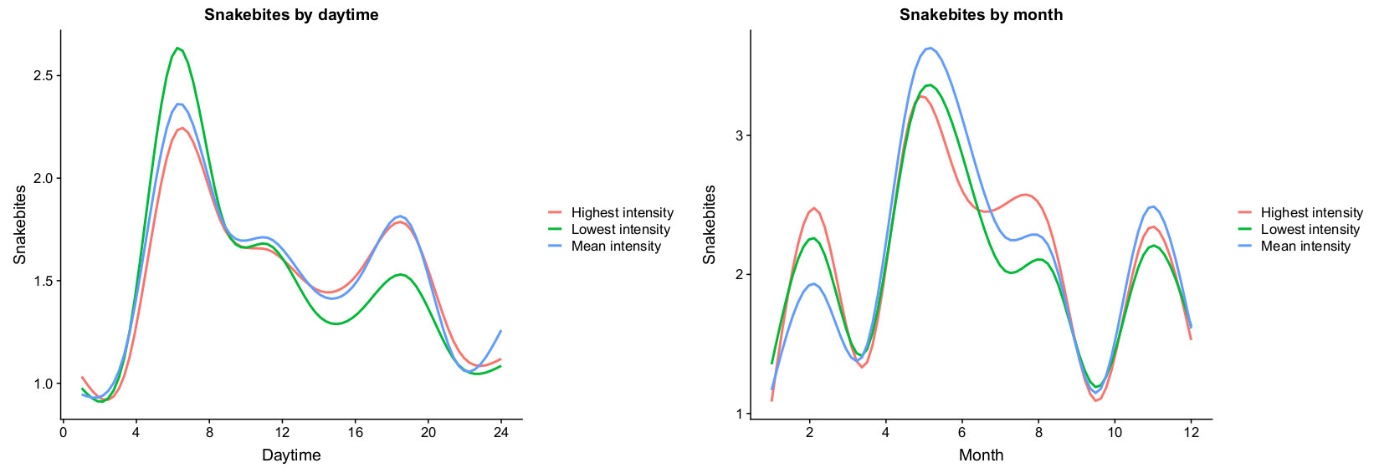


Figure 22: Changing the index value had almost no effect on the patterns of snakebites both on the daily level and a moderate effect on monthly level, with minor differences in the August peak between the different index values.


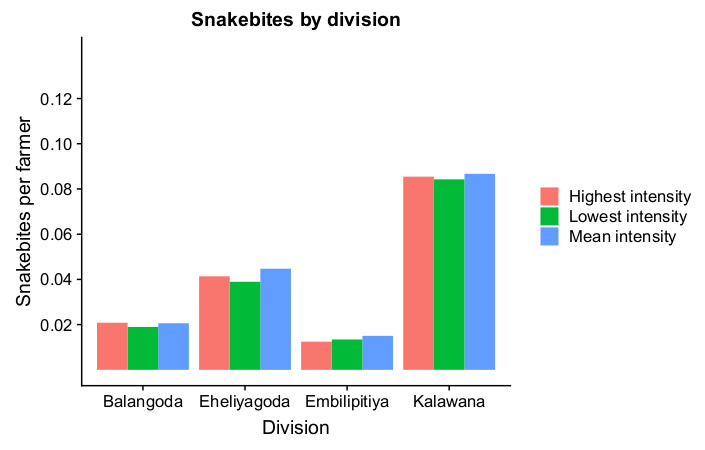
Figure 23: Changing the work index values had only small differences in the total number of snakebites on the division level.


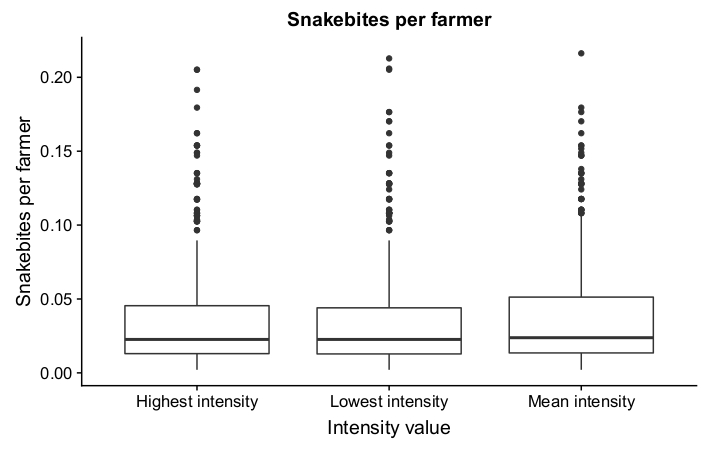


Figure 24: Changing the work index values had not difference in the total number of snakebites. In this output measurement our system showed robustness to the variation in values that we collected during our field work.


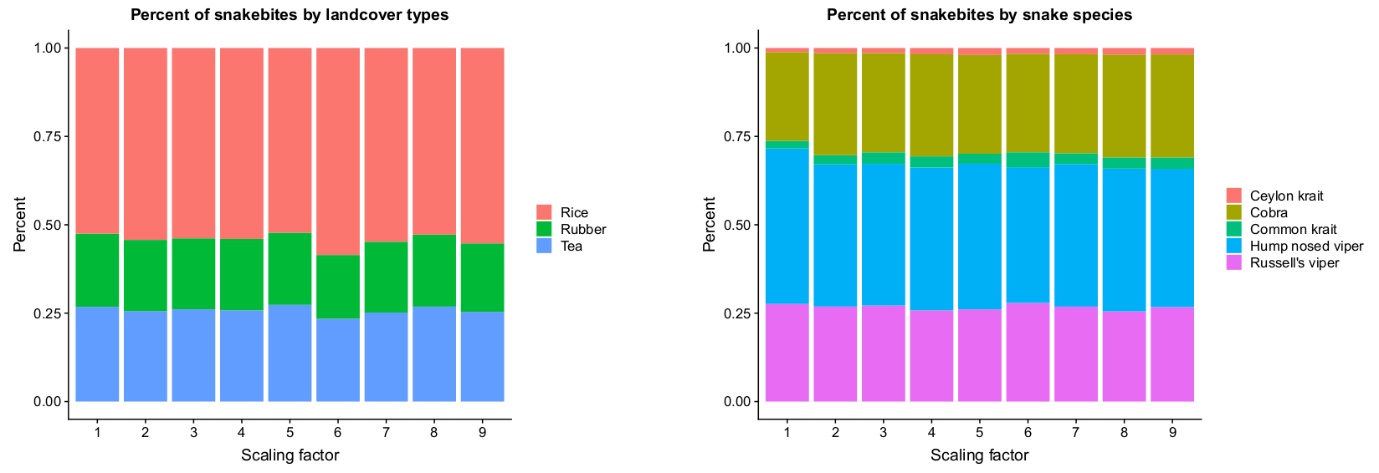


Figure 25: Our simulation was executed 30 times for each population factor at each one of the locations modelled, where changed the factor that was used for scaling up the PPM models by values that ranged between 1x10^10^ and 9x10^10^. Different population sizes had only a minor effect on either the percentage of snakebites on each landcover type and the percentage of bites caused by each one of the snake species.


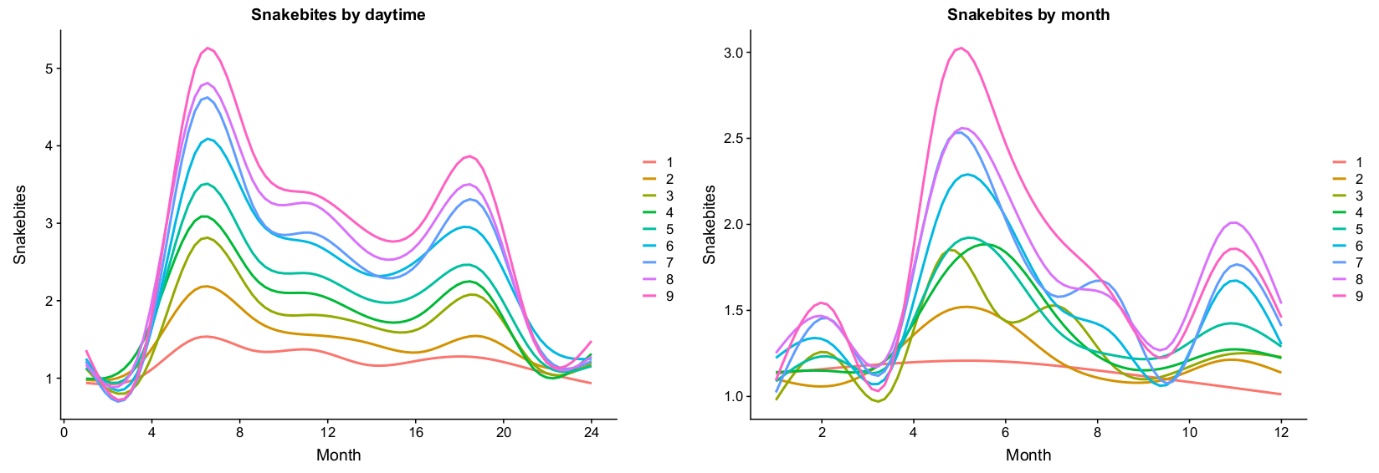


Figure 26: changing the population size had a change in magnitude in the number of snakebites across days, and across months. Below a certain population size the monthly pattern of snakebites stopped showing a distinct yearly pattern where there are distinct major peaks in snakebites, as well as distinct peaks in the daily distribution of snakebites.


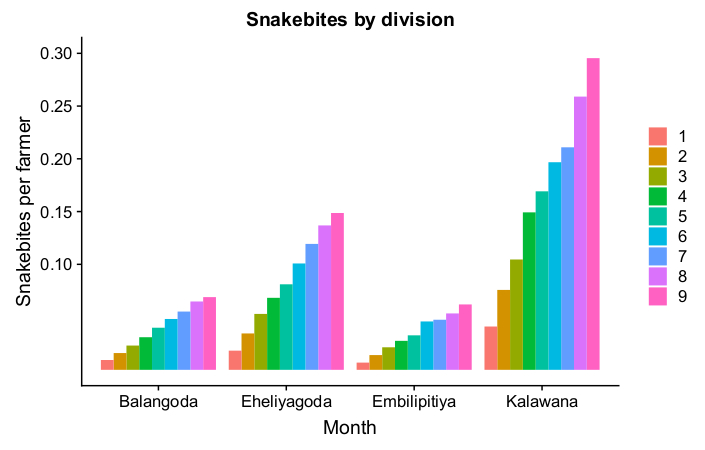


Figure 27: The different population sizes effect the different divisions in distinct ways. While all divisions showed a linear increase in number of snakebites as the snake population was increased, the rate of increase between regions was different.


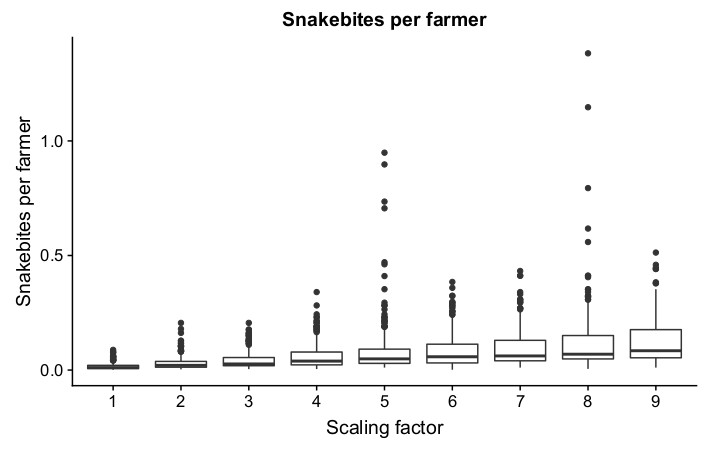


Figure 28: Different population sizes tended to increase the mean number of snakebites, but also increase the variance in number of snakebites per farmer. The larger population sizes had a larger uncertainty level regarding number of bites per farmer.
