# Supplementary material for: Integrating human behavior and snake ecology with agent-based models to predict snakebite in high risk landscapes
Source: PLoS Negl Trop Dis. 2021 Jan 22;15(1):e0009047. doi: 10.1371/journal.pntd.0009047 (PMC7857561; doi:10.1371/journal.pntd.0009047)
Supplement: Attachment — Submitted filename: PNTD-D-20-01368_reviewed.pdf [file pntd.0009047.s016.pdf]

PLOS Neglected Tropical Diseases

Integrating human behavior and snake ecology with agent-based models to predict snakebite in high risk landscapes

--Manuscript Draft--

**Manuscript Number:**	PNTD-D-20-01368
**Full Title:**	Integrating human behavior and snake ecology with agent-based models to predict snakebite in high risk landscapes
**Short Title:**	Agent-based models to predict snakebite in high risk landscapes
**Article Type:**	Research Article
**Keywords:**	snakebite; agent based modeling; ecological epidemiology; farmers behavior
**Abstract:**	Snakebite causes more than 1.8 million envenoming cases annually and is a major cause of death in the tropics especially for poor farmers. While both social and ecological factors influence the chance encounter between snakes and people, the spatio-temporal processes underlying snakebites remain poorly explored. Previous research has heavily focused on statistical correlates between snakebites and ecological, sociological, or environmental factors, but the human and snake behavioral patterns that drive the spatio-temporal process have not yet been integrated into a single model. Here we use a bottom-up simulation approach using agent-based modelling (ABM) parameterized with datasets from Sri Lanka, a snakebite hotspot, to characterise the mechanisms of snakebite and identify risk factors. Spatio-temporal dynamics of snakebite risks are examined through the model incorporating six snake species and three farmer types (rice, tea, and rubber). We find that snakebites are mainly climatically driven, but the risks also depend on farmer types due to working schedules as well as species present in landscapes. Snake species are differentiated by both distribution and by habitat preference, and farmers are differentiated by working patterns that are climatically driven, and the combination of these factors leads to unique encounter rates for different landcover types as well as locations. Validation using epidemiological studies demonstrated that our model can explain observed patterns, including temporal patterns, and relative contribution of bites by each snake specie. Our predictions can be used to generate hypotheses and inform future studies and decision makers. Additionally, our model is transferable to other locations with high snakebite burden as well.
**Additional Information:**	
**Question**	**Response**
**Financial Disclosure**<br><br>Enter a financial disclosure statement that describes the sources of funding for the work included in this submission. Review the submission guidelines for detailed requirements. View published research articles from *PLOS NTDs* for specific examples.<br><br>This statement is required for submission and **will appear in the published article** if the submission is accepted. Please make sure it is accurate.	This work was supported by the Medical Research Council [MP/P024513/1]. The grant was obtain by AP, KM, TI, DGL, HJdeS, and PJD. The funders had no role in study design, data collection, and analysis, decision to publish, or preparation of the manuscript.

Powered by Editorial Manager® and ProduXion Manager® from Aries Systems Corporation

**Unfunded studies**<br><br>Enter: *The author(s) received no specific funding for this work.*<br><br>**Funded studies**<br><br>Enter a statement with the following details:<br>• Initials of the authors who received each award<br>• Grant numbers awarded to each author<br>• The full name of each funder<br>• URL of each funder website<br>• Did the sponsors or funders play any role in the study design, data collection and analysis, decision to publish, or preparation of the manuscript?<br>• **NO** - Include this sentence at the end of your statement: *The funders had no role in study design, data collection and analysis, decision to publish, or preparation of the manuscript.*<br>• **YES** - Specify the role(s) played.<br><br>* typeset	
**Competing Interests**<br><br>Use the instructions below to enter a competing interest statement for this submission. On behalf of all authors, disclose any competing interests that could be perceived to bias this work—acknowledging all financial support and any other relevant financial or non-financial competing interests.<br><br>This statement **will appear in the published article** if the submission is accepted. Please make sure it is accurate. View published research articles from *PLOS NTDs* for specific examples.	The authors have declared that no competing interests exist

**NO authors have competing interests**<br><br>Enter: *The authors have declared that no competing interests exist.*<br><br>**Authors with competing interests**<br><br>Enter competing interest details beginning with this statement:<br><br>*I have read the journal's policy and the authors of this manuscript have the following competing interests: [insert competing interests here]*<br><br>* typeset	
**Data Availability**<br><br>Authors are required to make all data underlying the findings described fully available, without restriction, and from the time of publication. PLOS allows rare exceptions to address legal and ethical concerns. See the PLOS Data Policy and FAQ for detailed information.<br><br>A Data Availability Statement describing where the data can be found is required at submission. Your answers to this question constitute the Data Availability Statement and **will be published in the article**, if accepted.	Yes - all data are fully available without restriction

**Important:** Stating 'data available on request from the author' is not sufficient. If your data are only available upon request, select 'No' for the first question and explain your exceptional situation in the text box.<br><br>Do the authors confirm that all data underlying the findings described in their manuscript are fully available without restriction?	
**Describe where the data may be found in full sentences. If you are copying our sample text, replace any instances of XXX with the appropriate details.**<br><br>• If the data are **held or will be held in a public repository**, include URLs, accession numbers or DOIs. If this information will only be available after acceptance, indicate this by ticking the box below. For example: *All XXX files are available from the XXX database (accession number(s) XXX, XXX.*).<br>• If the data are all contained **within the manuscript and/or Supporting Information files**, enter the following: *All relevant data are within the manuscript and its Supporting Information files.*<br>• If neither of these applies but you are able to provide **details of access elsewhere**, with or without limitations, please do so. For example:<br><br>*Data cannot be shared publicly because of [XXX]. Data are available from the XXX Institutional Data Access / Ethics Committee (contact via XXX) for researchers who meet the criteria for access to confidential data.*<br><br>*The data underlying the results presented in the study are available*	All relevant data are within the manuscript and its Supporting Information files.

from (include the name of the third party and contact information or URL).<br>• This text is appropriate if the data are owned by a third party and authors do not have permission to share the data.<br><br>* typeset	
Additional data availability information:	

Manuscript Click here to access/download;Manuscript;Goldstein et al..docx

Type: Article

Title: Integrating human behavior and snake ecology with agent-based models to predict snakebite in high risk landscapes

Authors: Eyal Goldstein[1]*, Joseph J. Erinjery[1], Gerardo Martin[2,3], Anuradhani Kasturiratne[4], Dileepa Senajith Ediriweera[5], Hithanadura Janaka de Silva[6], Peter Diggle[7,8], David Griffith Lalloo[9], Kris A. Murray[2,3,10], Takuya Iwamura[1,11]

Affiliations:

[1] *School of Zoology, Department of Life Sciences, Tel Aviv University, Israel*

[2] *MRC Centre for Global Infectious Disease Analysis, Department of Infectious Disease Epidemiology, School of Public Health, Imperial College London, UK*

[3] *Grantham Institute - Climate Change and Environment, Imperial College London, UK*

[4]*Department of Public Health, Faculty of Medicine, University of Kelaniya, Kelaniya, Sri Lanka*

[5]*Centre for Health Informatics, Biostatistics and Epidemiology, Faculty of Medicine, University of Kelaniya, Ragama, Sri Lanka*

[6]*Deparment of Medicine, Faculty of Medicine, University of Kelaniya, Ragama, Sri Lanka*

[7]*CHICAS, Lancaster University Medical School, Lancaster, United Kingdom*

[8]*Johns Hopkins Bloomberg School of Public Health, UNITED STATES*

[9]*Liverpool School of Tropical Medicine, Liverpool, United Kingdom*

[10]*MRC Unit the Gambia at London School of Hygiene and Tropical Medicine, Fajara, The Gambia*

[11]*Department of forest ecosystem and society, Oregon state University, Corvallis, Oregon, United States*

* Corresponding Author

Funding:

This work was supported by the Medical Research Council [MP/P024513/1]. The grant was obtain by AP, KM, TI, DGL, HJdeS, and PJD. The funders had no role in study design, data collection, and analysis, decision to publish, or preparation of the manuscript.

Abstract:

Snakebite causes more than 1.8 million envenoming cases annually and is a major cause of death in the tropics especially for poor farmers. While both social and ecological factors influence the chance encounter between snakes and people, the spatio-temporal processes underlying snakebites remain poorly explored. Previous research has heavily focused on statistical correlates between snakebites and ecological, sociological, or environmental factors, but the human and snake behavioral patterns that drive the spatio-temporal process have not yet been integrated into a single model. Here we use a bottom-up simulation approach using agent-based modelling (ABM) parameterized with datasets from Sri Lanka, a snakebite hotspot, to characterise the mechanisms of snakebite and identify risk factors. Spatio-temporal dynamics of snakebite risks are examined through the model incorporating six snake species and three farmer types (rice, tea, and rubber). We find that snakebites are mainly climatically driven, but the risks also depend on farmer types due to working schedules as well as species present in landscapes. Snake species are differentiated by both distribution and by habitat preference, and farmers are differentiated by working patterns that are climatically driven, and the combination of these factors leads to unique encounter rates for different landcover types as well as locations. Validation using epidemiological studies demonstrated that our model can explain observed patterns, including temporal patterns, and relative contribution of bites by each snake specie. Our predictions can be used to generate hypotheses and inform future studies and decision makers. Additionally, our model is transferable to other locations with high snakebite burden as well.

Introduction

Globally, five million people are bitten by snakes every year, resulting in approximately 94,000 deaths out of 1.8 million envenoming cases, and up to 400,000 morbidities [1,2]. Most of this burden occurs in the tropics of south east Asia and Sub Saharan Africa [2]. Despite its impacts, snakebite is still considered a neglected tropical disease that is concentrated among the poorest of the poor [2,3], and this may have contributed to the lack of funding and scientific research on snakebite relative to other disease of similar or lesser burden [3–5]. In 2017 snakebite was declared a neglected tropical disease by the World Health Organization [3], which prompted the scientific community to increase efforts for combating this disease, including the development of a global snake bite strategy and roadmap [6].

Several past studies have hypothesized on the importance of overlap between snake and human activities as a cause of snakebite patterns (e.g. [7,8]). However, previous research on snakebite has relied heavily on correlative models, that statistically relate bite data (e.g., from hospital admissions) to a range of social and, less often, environmental variables to identify key risk factors [9]. Such studies include those which incorporate climatic factors such as precipitation, humidity, and mean temperature [4,10–12], social factors including human population density, poverty, and farming activities [4,11,13–16], and ecological factors such as snake activity or distribution information [10,13,17,18]. For example, Yañez-arenas et al., (2016) show a correlation between snake distributions and bites, and Akani et al., (2013) matched patterns of snake activity with agricultural activity of local farmers across different months to reveal correlation with snakebite occurrences. However, no studies have yet taken a mechanistic socio-ecological approach that integrates both human and snake distributions and behaviors to investigate the ways in which snakebite epidemiology is simultaneously shaped by ecology, climate, and landscape characteristics.

Agent based modelling (ABM) is a bottom up approach for modeling complex and adaptive systems. ABMs are comprised of collections of individuals (agents) that are programmed to display behavioral traits, while their interactions with each other generate phenomena at a higher level [19–22]. ABM is used both for representing the internal dynamics of complex systems, and discovering emergent patterns that may be found in those systems [23,24]. Spatially explicit social-ecological dynamics are increasingly modelled using an ABM approach (e.g.: [21,25,26]), such as those involving land use and land cover change [25,27,28]. ABM has also been used for modelling ecological epidemiology, including zoonotic disease transmission across landscapes (e.g.: [29]), mosquito behavior in models for malaria transmission [30], rabies transmission among foxes [31], and the spread of foot and mouth disease [32]. With snakebite sharing many socio-ecological characteristics with zoonotic diseases [9], ABM is an ideal and novel approach to investigate the epidemiology of snakebite from a mechanistic perspective (see Figure 1).

Sri Lanka is a global snakebite hotspot [2]. It has been estimated that nationally there are more than 80,000 snakebites a year, 30,000 of which involve envenoming. Due to high quality health systems, only around

400 of these result in deaths annually [11]. Nevertheless, morbidity is considerable and the total annual economic burden on households of snakebite envenoming in Sri Lanka amounts to almost $4 million, while it costs the public health system around $10 million per year [33]. Sri Lanka is home to over a hundred snake species, as well as nine medically important land snake species, including: *Daboia russelii*, *Naja naja*, *Bungarus caeruleus*, *Bungarus ceylonicus*, *Echis carinatus*, *Hypnale hypnale,* [34]. Many of these species also contribute to an extensive burden in neighboring regions in South Asia [35]. Previous studies have shown that the frequency of snakebites in Sri Lanka is spatially correlated with climatic, geographic, and socio-economic factors, such as ethnicity, age, occupation, and income [11], with bites occurring seasonally (primarily in the months of November-December, March-May and August) [7]. Snakebite incidence is broadly congruent with the geographical patterns of snake species occurrence across the island [36].

In this study, we integrate socio-ecological factors associated with snakebites in Sri Lanka into a single model by constructing an ABM simulation based on detailed datasets of snake distributions, snake behaviors, landscape characteristics, and farmers' behavioral patterns. Sri Lanka provides an ideal case study for modeling snakebite mechanisms in this way, as not only is it a global hotspot of snakebite, it also provides highly reliable snakebite incidence data and has a high volume of accumulated medical research from which the model can be developed and validated [7,11,34,36]. We developed a spatially explicit ABM to analyze the spatio-temporal overlap between the different medically important snake species and farmers of different crops in Sri Lanka, and integrated climate and landcover as drivers of human-snake interaction across different affected landscape, in order to create a predictive model that can inform both future research as well as decision makers.

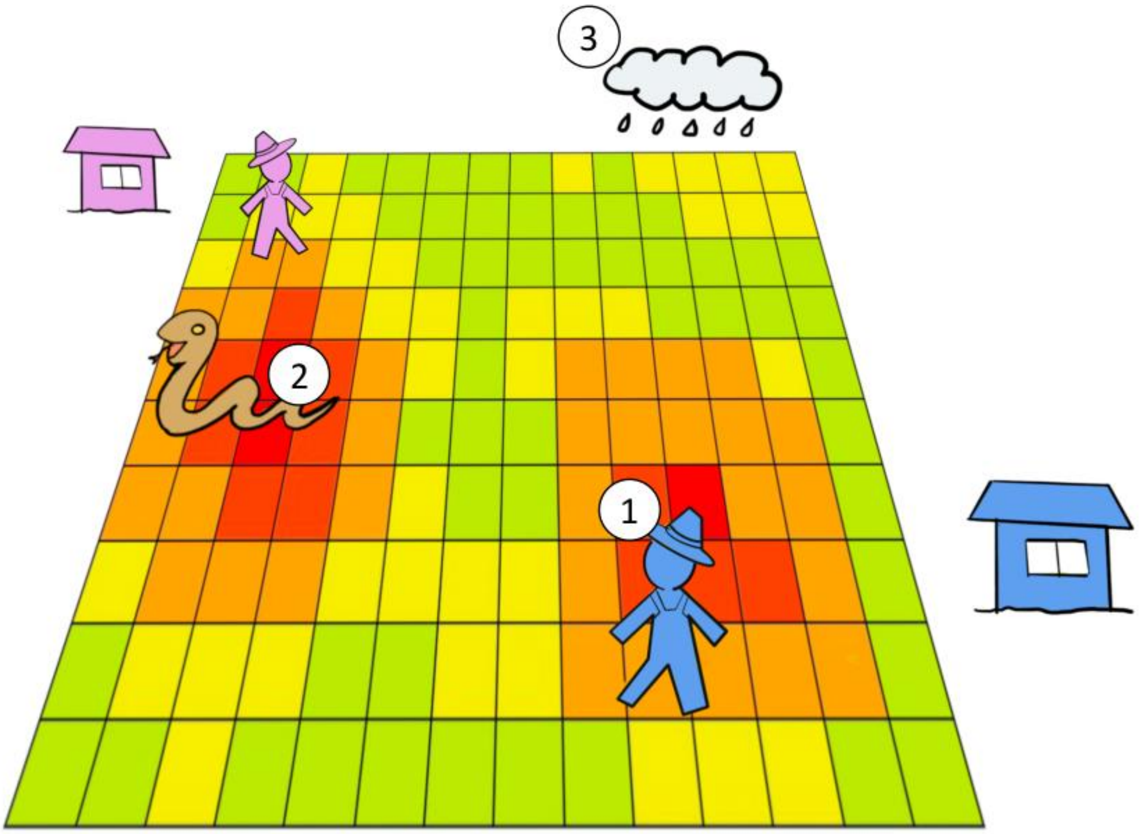

Figure 1: Modeling approach: Our model simulates daily and seasonal cycles. A day is represented as 24 time steps. **1. Farmer agent**: Farmer agent has its own daily/seasonal activity schedule according to farmer types (rice, tea, rubber). It owns its piece of crop land. Farmer agent commutes from its home location to its field. It moves inside of her crop area. **2. Snake activity layer:** Snake activity level is determined by the snake species, crop types (habitat types) and precipitation. Snake species determines its distribution probability, habitat preferences, daily/seasonal activity schedule and attack rate. **3. Precipitation cycle**: Precipitation affects snake activity and farmer's activity.

Materials and Methods

Ethics statement

Our research has been reviewed by the ethics review committee of the faculty of medicine, university of Kelaniya, reference number p/22512/2018. Our study included permission of consent by all participants who were interviewed during the field work.

1. Agent based modeling

Agent based modelling (ABM) is a bottom-up approach for modeling complex and adaptive systems using autonomous agents, where decisions by individuals is used for explaining macro level phenomenon [19–22]. ABM is used both for explaining complex phenomenon that are not easily reducible to differential equations, and discovering emergent patterns and phenomenon found in those systems, as well as study the internal dynamics of these system [23,24]. ABM has been extensively used in different field of study for modeling complex phenomenon, such as social, political, and economical science [23]. There are now multiple programs used for ABM, including NetLogo [37], Repast [38], as well as the SpaDES package in R. Recently, spatially explicit social-ecological dynamics are increasingly modelled using an Agent-based modeling approach (e.g.:[21,25,26,39]). It is commonly used for modelling social behavior including modeling land use and land cover change [25,27,28], as well as zoonotic disease transmission across landscape (e.g.:[29]).

We used Netlogo [37] to develop a spatially explicit model that represents the dynamics of snakebites among farmers (S1 Figure 1-2). The model simulates real landscapes in the Study Area (see above), each of which is represented by a 2x2 km study location comprised of a matrix of 10x10m grid cells. We simulated 17 study locations in total.

For the design and analysis of our model we used pattern oriented modelling (POM) [40,41]. This approach emphasizes use of multiple patterns at different hierarchical scales for calibration and validation in order to reduce uncertainty in model structure and paraments. This approach allows us to examine not only large scale phenomena (such as macro level epidemiological observations), but also probe the dynamics and intricacies of the mechanism(s) that may be hidden or unobservable by just examining the different patterns individually.

The pattern oriented modelling protocol is comprised of four steps [40]: 1) aggregate known biological data regarding a system and use it to construct a model that is related to a hypothesis and is theoretically capable of reproducing previously observed patterns; 2) determine the parameter values of the system; 3) compare systematically between the independently observed data and those patterns predicted by the model, which may involve iteratively improving the model by removing certain parameters or choosing combinations of parameters that are more plausible or better represent observed patterns; and 4) look for secondary predictions in the model, which are different from the original patterns to which the model was compared during the third step of the process.

For each one of the locations studied, the model uses a range of input data (see below) to simulate the movement and interactions of different 'agents' among cells for a fixed duration of time. We used a discrete time series comprised of both months and hours. Each month is condensed to 24 timesteps which are representative of individual hours of the daytime, and the simulation is performed across the 12 months of the year, comprising of 288 timesteps in total. Parameters and variables in the simulation are recorded and

updated both hourly and monthly, depending on the agent (snake seasonal activity and farmers' working schedules update at the beginning of each month; snake daily activity is updated at the beginning of each hour, see below).

There are two types of agents in the model: farmers and snakes. Farmers are abl[illegible]ork in multiple land cover classes, depending on seasonal needs (see 'Recording Farmer Characteristics' below). Farmers have a state variable of working schedule, which includes the land cover type they should be farming, time of day they begin to work, and the number of hours they will spend working in that land cover class. Using the work schedule, the farmers move between the land cover they are farming and their home.

Each snake agent is characterized by a set of ecological and behavioral traits, including: species, daily activity, habitat preference, aggressiveness, and seasonal act[illegible]ess. Each species is given a set of probabilities for movement between land cover classes depending on the land association factor (see "Snake distribution and behaviour" below) and number of patches for each land cover class (see "Remote sensing dataset" below).

The influence of the environment on agent activity is represented by climatic variables (precipitation and number of non-rainy days (see "Climate dataset" below)).

2. Study area and spatial data

We focused our modelling effort on the district of Ratnapura in the wet zone of Sri Lanka, which is characterized by high precipitation (see Figure 2). This district has a great diversity of crop types, including tea, rubber, coconut, as well as rice cultivation albeit practiced here on a smaller scale in comparison to other zones of Sri Lanka due to topographic conditions [42–44]. Within the district, we focused our research on four different divisions (Eheliyagoda, Balangoda, Kalawana, and Embilipitiya) that represented the variation in crop types within the district, and at each division level we ran simulations on between 4-5 locations, with 17 locations in total (see Figure 3).

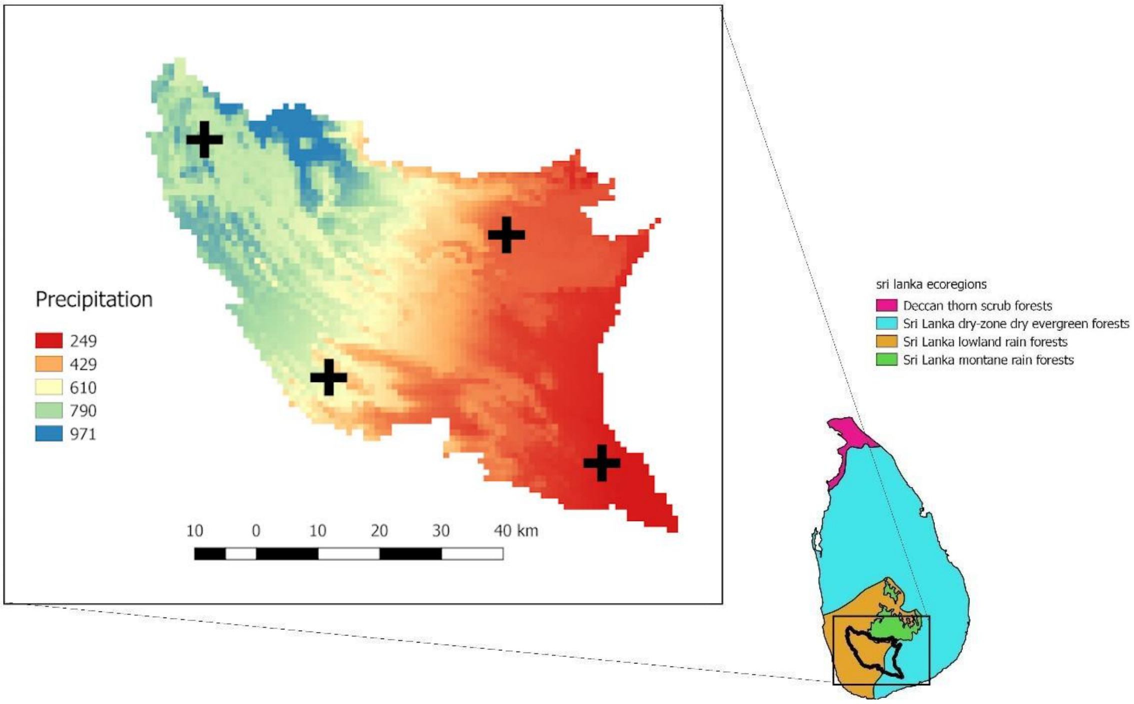

Figure 2: Ecoregions of Sri Lanka [45]. Annual precipitation of the Ratnapura district (Bioclim variable 12; [46]). The four different divisions (Eheliyagoda – northwest, Kalawana – southwest, Balangoda – northeast, and Embilipitiya - southeast) used in analyses are marked. The Ratnapura district borders on the highlands in the center of the country, the dry zone in the south east, and is part of the wet zone in its center and west.

Landcover - The main attribute of each cell in the model is its landcover type (Rice, Tea, Forest, Rubber, Home). We used Sentinel-2 remotely sensed images from 2017 to produce vegetation type classification maps (Tile T44NMN and relative orbit numbers R119 & R076), which were chosen based on quality of images and percentage of cloud cover. Tiles were downloaded from the USGS earth explorer portal and were processed using the SNAP program and the Sen2cor plugin [47]. After removing cloud cover, the tiles were merged into a single tile before classification.

We classified the images into five different landcover types giving importance to major crop types and vegetation in the district: forest, rubber, tea, paddy, and water bodies, with a resolution of 10 x 10m (Figure 3). The classification was made using two different supervised classification algorithms: support vector machine (SVM) and maximum likelihood (ML), with 100 training polygons for each land cover type. We used spectra from 4 different bands and NDVI index for classification (band number 2 – Blue, band number

3 – green, band number 4 – red, and band number 8 – near-infra red), with band numbers 4 and 8 used for calculation of the NDVI index. We obtained an overall accuracy of 83.2% and kappa coefficient of 0.68 for the SVM classification and an accuracy rate of 80.7% and kappa coefficient of 0.66 for the ML classification (see accuracy assessment in S2 Tables 1-2). The classification was later supplemented with a home class, where homes were randomly assigned in each study location in proportion to the population, with a fixed population size of 200 farmers for each simulation.

Climate - We used monthly precipitation (mm) from the climate research unit dataset [48] downscaled to a resolution of 1km^2, using the Delta method [46,49]. For each one of the locations modelled, we extracted the raster values and used them in our model as integer values for each month. In addition, we estimated the number of non-rainy days per month from past literature [50].

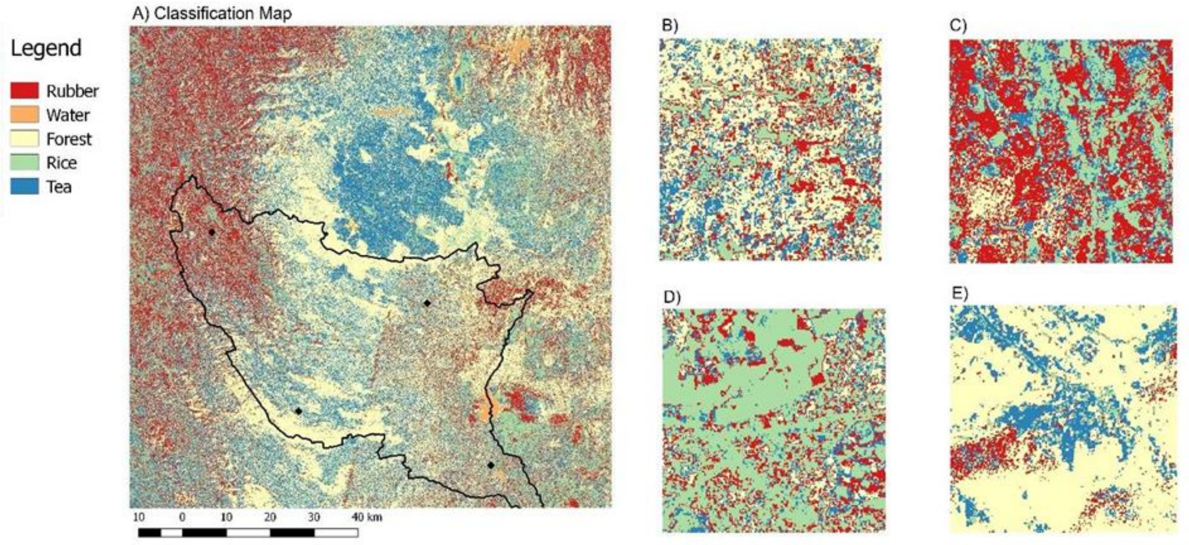

Figure 3: Classification map using support vector machine (SVM). A) Classification map for the Ratnapura region created using a SVM classification and sentinel 2 satellite imagery. The Ratnapura district border is marked on the map (black line), and the four divisions where we conducted field work and ran simulations are marked (black dots, see also Figure 2). Variation in landcover types can be observed between locations, with B) the north east (Balangoda) having a mixture of all landcover types, C) the north west (Eheliyagoda) containing a high concentration of rubber plantations, D) the south east (Embilipitiya) containing a high concentration of rice farming, and E) the south west (Kalawana) containing many tea plantations next to forests.

3. Human agent characteristics

Farmer activity – The characteristics and behavior of farmers in the study area (see above) was first characterized via a community survey conducted during two weeks in July 2019. We visited four different divisions in the district of Ratnapura, and in each one we interviewed 10 farmers (40 in total) of different crops: with 22 engaged in rice farming, 22 in tea farming, and 10 in rubber (some farmers tend multiple crops). Each farmer was asked to answer a set of questions related to work schedules, including: planting season, harvest season, hour of starting work, hour of finishing work, seasonal rotation of crops, as well as size of plot. We also asked farmers about previous encounters with snakes, including location, and season when snakes were encountered . Our final farming dataset included a list of parameters that defined the farming behavior in the model (see Table 1).

Table 1: A complete list of parameters used in the model for all agent types. Each of the parameters is either an input for the snake behavior submodel, farmer behavior submodel, or a global variable (climate and landcover).

**Model**	**Parameter**	**Value**	**Source**
Farmers	Farmer type	Rice, Rubber, Tea	Field work
Farmers	Land type work index	0-110	Field work
Farmers	Starting hour	4-9AM	Field work
Farmers	Number of hours worked	4-14	Field work
Farmers	Percentage of population working as farmers	30-70%	Government reports
Snake	Point process models	0-3*10^-8	Martin et al 2019
Snake	Seasonal activity probability	0-1	Literature
Snake	Daily activity patterns	0-.5	Literature
Snake	Aggressiveness	1-10	Local herpetologists questionnaire
Snake	Land association factor	0-2.429	Calculated from snake data
Land cover	Type of land cover	Rice, Tea, Rubber, Forest, Water, Home	Remote sensing
Climate	Mean monthly precipitation	21-1054	Climate Research Unit
Climate	Number of rainy days	10-25	literature

Based on the results of the survey, we allowed farmer agents in the model to have the option of moving among up to three different landcover types, and to choose between different working schedules on each landcover type. To take into account the seasonal variation of labour requirements according to the various cropping cycles, we first developed a labor index:

$$(1)\quad I_{ij} = \frac{(247 \times F_{ij}) \div A_i}{30 \div D_{ij}}$$

where I_{ij} is the labor index for landcover *i* during month *j* for 1 square kilometer of that landcover, F_{ij} is the number of farmers needed at landcover *i* during month *j* for the size of landcover owned by a specific farmer, A_i is the size of landcover *i* in acres, and D_{ij} is the number of days per month that land cover *i* is farmed during month *j*, and 247 is used to convert acres (the measurement farmers used when answering the questionnaire) into square kilometers.

A mean value of I_{ij} was calculated using the different index values obtained by the farmers and was distributed between the months according to the working schedule described by the farmers in the

interviews. For the rubber landcover the index was calculated for a single day, and then multiplied by the estimated number of non-rainy days that occur in that specific month, since rubber farmers cannot work in the rain due to technical limitations of rubber harvesting methods.

In the model, the probability of each farmer attending each landcover type is then calculated at the beginning of each month:

$$(4)\quad W_{ij} = \frac{S_j \times I_{ij}}{W_{max}} \times P$$

where W_{ij} is the number of farmers that are going to work in month *i* in landcover type *j*, S_j is the size of landcover type *j* in a simulation, I_{ij} is the labor index for month *i* and landcover *j* (from eq 1), W_{max} is the maximum value of *W* possible for the location being simulated, and *P* is the farmer population size of the location being simulated. Once a farmer is assigned a certain landcover for month *i*, they will only work on that specific landcover during that month.

The farmers are then assigned a random number from a uniform distribution composed of the possible number of hours farmers work in the field for that specific landcover, based on what was reported by the farmers interviewed during the field work (S2 Table 4). For the starting hour, the farmers choose a random value out of a normal distribution composed of the possible starting hours for that specific landcover, based on what was reported by the farmers during the field work (S2 Table 5).

4. Snake agent characteristics

Distribution and abundance - We used Poisson point process models (PPMs) to represent potential abundance of snakes for each species [51]. We interpreted these models as representing the relative carrying capacity and a proxy for potential abundance for each species in each one of the locations modelled in our simulation. In order to calibrate our model's snake population size, we used previous research in which the species *Hypnale hypnale* was systematically surveyed to estimate the number of individuals per square kilometer of forest habitat [52]. This provided a link between PPM outputs and measured snake abundance in forest landscapes, which we then applied to other species and habitat types according to relative model weights following a habitat preference analysis (see below). This method resulted in abundance estimates up to 900 individuals per species per 2x2km tile (= up to 225 snakes per species per km^2).

Habitat preferences - Preference of landscape for each snake species was defined by a land association factor, calculated using the data points that were used to create the species distribution models. Using chi-square tests, the likelihood of a snake species being found on a specific land cover versus the probability that it would be found there at random was calculated (Martin et al., unpublished). For this calculation we used land cover maps produced by Erinjery et al. (*unpublished*).

Activity and behaviour – We incorporated several different measures of snake activity and behavior into the model, including seasonal activity patterns, daily behavioural habits, movement preferences among available habitats, and aggressiveness.

In the model, we assumed that there are a fixed number of snakes for each species present on a tile based on the PPM maps and population size estimate. Changes in activity levels throughout the year were defined according to observed seasonal activity in the tropics [53–55], and according to observations made on *Hypnale spp* [52]. At each monthly update a certain percentage of the snakes from each species becomes active according to the level of precipitation measured (see section 4), as calculated by:

$$(2) \quad A_i = \frac{P_i}{P_{max}}$$

where A_i is the activity factor for month i , and P_i is the precipitation level for month i, and P_{max} is the max level of precipitation for the region.

The snake daily activity is determined probabilistically according to the snake activity patterns, with each species being pre-defined as either diurnal, nocturnal, crepuscular, or cathemeral [56]. A probability distribution was designed for each of the different daily activity patterns by identifying hours of sunrise and sunset, and setting the distributions in relation to those hours. All snakes were defined to have a baseline probability of 0.1 (10% chance) for being active even in hours when they are biologically defined as inactive, e.g. nocturnal snakes during daytime, in order to capture the full scope of encounter probability as described by epidemiological surveys (see below).

The probability of snakes moving to a specific landcover type is calculated using the amount of landcover type available and the attraction of the snake to that specific landcover type (see S2 Table 6 for the land association factor). The probability of each species moving to any type of landcover type was defined by a transition rule as:

$$(3) \quad M_{ij} = \frac{P_j L_{ij}}{P_1 L_{i,1} + \cdots + P_n L_{i,n}}$$

where M_{ij} is the probability of an individual of snake species i to move to land cover type j, P_j is the number of cells of land cover j, and L_{ij} is the landcover association factor between snake species i and landcover j. After calculating the transition rule, a random number is drawn to decide what landcover the snake will move to.

5. Snakebites

Agents are tracked within the model locations and their encounters (occurring in the same grid cell at the same time) reco[illegible]. The probability of a snakebite occurring during an encounter is calculated by taking into account the varying propensities of each species to attack during an encounter. We incorporated

aggressiveness by way of an aggressiveness index, which is a ranking of between 1-10 (1= docile, 10= very aggressive) as determined by local herpetologists (Table 2). The probability of a snakebite occurring is therefore calculated as:

$$(5)\ P_i = \frac{A_i}{A_{max}}$$

where P_i is the probability of snake species i causing a snakebite when there is a human-snake interaction, A_i is the aggressiveness index for snake i, and A_{max} is the maximal value for aggressiveness. When humans and snakes meet on the same cell, a random number is drawn between 0-1, and if it is smaller than the value obtained from the calculation then a snakebite occurs.

Table 2: Snake behavior profiles for each species, as reported by local expert herpetologists. These profiles were integrated into the snake agent behavior variables, with the aggressiveness index and dial activity directly integrated into the model, and zonation is given as a broad description while the habitat preference factor was used in order to define snake behavior.

Species	Common name	Aggressiveness	Daily activity	Zonation
Daboia russelli	Russell's viper	8	Nocturnal	Terrestrial
Echis carinatus	Saw scaled viper	10	Cathemeral	Terrestrial
Hypnale hypnale	Hump nosed viper	10	Nocturnal	Semi-arboreal
Hypnale zara	Hump nosed viper	10	Nocturnal	Semi-arboreal
Hypnale napa	Hump nosed viper	10	Nocturnal	Semi-arboreal
Bungarus caeruleus	Common krait	2	Nocturnal	Terrestrial
Bungarus ceylonicus	Ceylon Krait	1	Nocturnal	Terrestrial
Naja naja	Cobra	5	Cathemeral/ Crepuscular	Semi-aquatic

6. Model evaluation

We evaluated our model in two different ways: hypothesis testing (verification) and validation. For validation we used the "multiple patterns" methodology in order to check for consistency between the model and the observed data. This was done to make sure we were not overfitting the model, and to make sure it represented the general dynamics of the system [41,57]. For the hypothesis testing we examined the process representation to make sure our model represented both the micro and macro level phenomenon

correctly, and that the system properly represented the dynamics and mechanism(s) that it is supposed to be representing. For validation we used the model formulations that were chosen during model selection. In addition, for the variables that were tested during the sensitivity analysis we chose variable values that were parameterized using the analysis output in order to make sure the values were above a threshold that allowed emergent patterns to appear in our system. For the full description of model selection and sensitivity analysis see S3.

Validation

For external validation we chose multiple patterns on which there was already research conducted in Sri Lanka, such as temporal patterns of snakebites [7], the relative risk of snakebite between locations [11], and biting snake species composition among bite victims as inferred from hospital records [36]. This was done in accordance with the POM protocol [41], which suggests that multiple patterns be assessed and the fit between the model predictions and these patterns evaluated (as opposed to comparing results to a single statistic or a single pattern). This is supposed to prevent overfitting of the model to an expected output, or falsely representing the model by using only one output parameter, and to make sure that the model can represent the dynamics of the system that it is attempting to represent.

Hypothesis testing

We checked for consistency of process representation, following the spatial and temporal patterns of the snake and farmer agents, and snakebites. We did this for the distribution of snakebites across both the months of the year and across the hours of the day. We then checked when peak snakebites were occurring and their relationship to the movement patterns of the agents. This allowed us to make sure that the system was properly representing both the micro level (agents' movements) and the macro level (snakebite distribution) and the relationship between them.

Hypothesis generation

The POM protocol also suggests looking for secondary predictions that emerge from the model and using them later for further validation if observations become available, and if not then using them to prompt further research in the field [40]. We checked for the following secondary predictions: monthly and daily patterns by snake species, by division, and by landcover type.

Results

Validation

Overall, the model performed well in differentiating between high and low risk locations. The results are based[illegible]mulation runs for 45 different locations across the entire district of Ratnapura, with high and low defined as above or below the median snakebite risk for all locations simulated. Predictions of the ABM showed a significant difference in prediction between locations where snakebite risk was above the median of all locations simulated and those where snakebite risk was below the median using Welch two sample t-test ($t = -5.5391$, $df = 39.198$, $p\text{-value} < 0.001$) (S1 Figure 1).

The model also effectively predicted the relative contribution of different species to overall snakebite patterns as derived from hospital surveys [36], both in divisions 1-[illegible]hich were locatated in the wet zone (Eheliyagoda, Balangoda, and Kalawana), and divions 4 (Emptilipitiya) which was located in the intermediate zone (Table 3 & Figure 4). The number of cobra bites was overestimated in our model in all locations. Additionally, in contrast to the hospital survey our model did not include non-venomous species, so an over estimation is to be expected to a certain extent.

Table 3: The average predicted proportion of bites from different snake species across four different locations. The first three divisions (Balangdoa, Eheliyagoda, Kalawana) belong to the wet zone of Sri Lanka, while the fourth region (Embilipitiya) belongs to the intermediate zone of Sri Lanka.

Wet zone (1-3)	Model prediction	Hospital data
Hump nosed viper	51-57%	65%
Russell's viper	21-24%	25%
Cobra	23-26%	5%
Non-venomous species		5%
Intermedia zone (4)	Model prediction	Hospital data
Russell's viper	[illegible]%	50%
Hump nosed viper	16[illegible]	30%

Common Krait	10%	10%
Cobra	33%	5%
Non-venomous species		5%

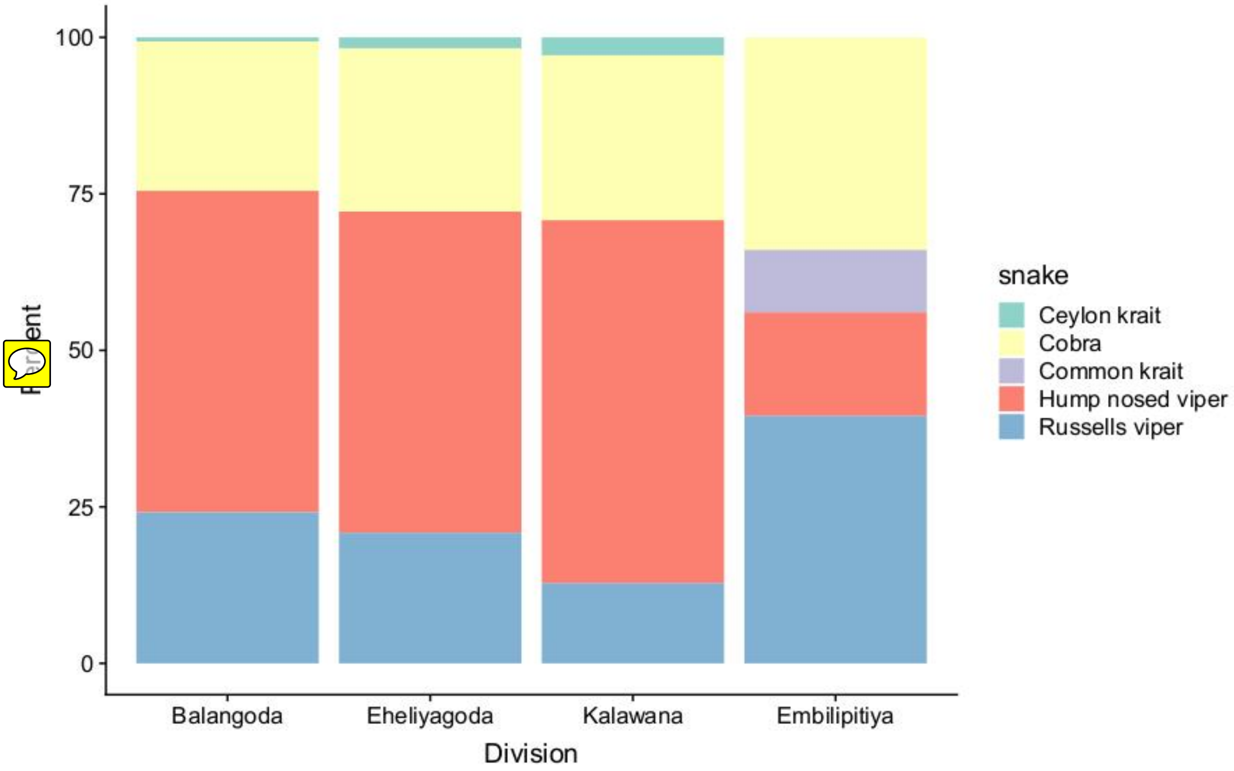

Figure 4: The average predicted proportion of bites from different snake species across four different locations. The first three divisions (Balangdoa, Eheliyagoda, Kalawana) belong to the wet zone of Sri Lanka, while the fourth region (Embilipitiya) belongs to the intermediate zone of Sri Lanka.

The model was also successful in predicting the temporal patterns of snakebite in Sri Lanka reported previously. Snakebite has been reported as having three peaks in general throughout the year (November–December, March–May, August), although there are regional variations [7]. The ABM predicted the possibility of different main peaks of snakebites through the year, including a large peak in March-May (Balangoda, Eyeliyagoda, Kalanawa, Embilipitiya), a second peak around August (Balangoda, Kalanawa), and a third peak in November-December(Balangoda, Eyeliyagoda, Kalanawa, Embilipitiya) (Figure 5).

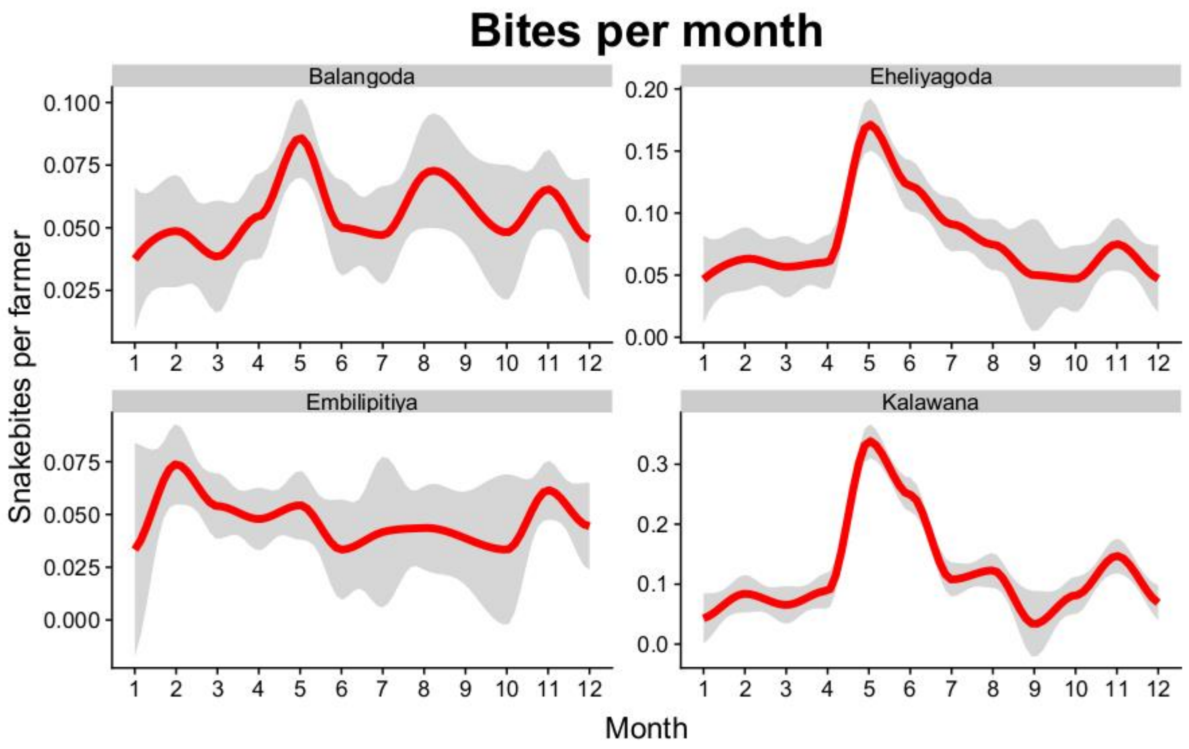

Figure 5: Snakebites per farmer across different months. Results are based on 30 simulation runs for each location across 4 divisions representing snakebite patterns across the year.

Hypothesis testing

The model performed well in representing the micro level (agent movement) and its relation to the macro level (snakebite distribution), with a clear pattern of spatial-temporal overlap between snakes and farmers as the cause of snakebites (Figure 6). The highest frequency of snakebite during the year occurred when both farmers and snakes were present and active on the different landcover types, although bite frequency differed among landcover types. On tea plantations, snakebites are simulated to follow snake activity closely as the activity level of farmers is highly consistent throughout the year (Figure 6A & 6D). Since the level of snake activity is defined by the amount of precipitation, the snakebites patterns follow seasonal rainfall (Figure 6A & 6D). For rice paddies, snakebite peaks occur at different time periods – either in April-May (peak snake activity), in August (peak farmer activity), or November (a combination of both) (Figure 6B & 6E). This reflects seasonal variability of rice farmers' behaviors, which have a different activity peak from snakes (Figure 6B & 6E). On rubber plantations, snakebites are a mixture of both snake and farmer activity as well, with the highest peak in bites occurring when snakes are most active in April-June (Figure 6C & 6F).

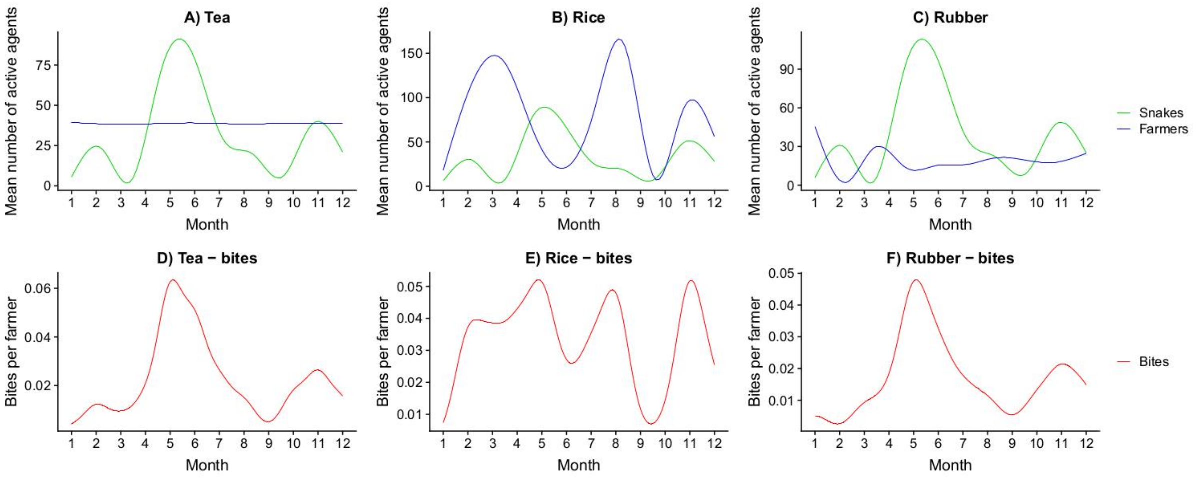

Figure 6: Spatio-temporal overlap between farmers and snakes for each land cover type. Values represent the mean number of farmers, snakes, and bites for 660 simulation runs across all locations. Each graph in the first row follows the monthly spatio-temporal overlap between farmers and snakes for **A)** tea **B)** rice, and **C)** rubber, and each graph in the second row follows the snakebite pattern that emerges out of the spatio-temporal overlaps for **D)** tea **E)** rice, and **F)** rubber **.**

Distinct patterns of spatio-temporal overlaps on the daily level are also evident. For the tea landcover, peak activity tends to follow a bimodal pattern with peaks occurring in both late afternoon and early morning (S1 Figure 2A). This pattern reflects the working pattern of tea farmers that tend to start working early during the day, but also follow long working hours, which results in farmers meeting snakes both when snakes are active early morning, and when snakes are active during late afternoon. For the rice land cover, snakebites have the highest probability of occurring during late afternoon when farmers and snakes have high overlap, but may also occur in the early morning during peak activity months (S1 Figure 2B). This pattern reflects the working pattern of rice farmers that tend to start later during the day, but work for long hours, there for increasing the chances of encounter while snakes are active later in the day. For rubber, snakebites have the highest probability of occurring during the early hours of the morning (S1 Figure 2C). This pattern reflects the working pattern of rubber farmers that tend to start working early in the day when snakes are active, but also have short working hours, so a second snakebite peak later in the day does not occur.

Hypothesis generation

A secondary prediction of our model was that the monthly burden of snakebites varies across locations, (Figure 7). Our model predicted that in drier locations the peak in bites occurs earlier in the year during February-April, whereas wetter locations tend to have a higher peak in bites during the month of May (Figure 7). The different patterns cannot be traced to a single factor but is likely caused by a combined

effect of land cover and climatic differences, and the interaction between snakes, farmers, and their environment between these locations (see S1 Figures 3-7). This prediction also suggests that there may be significant temporal differences in snakebites between the wet, dry, and intermediate zones in Sri Lanka.

Another secondary prediction from our model estimates that the monthly distribution of snakebites varied between species, with a different pattern for each species (Figure 7). These different patterns are not caused by snake activity alone, but by a combination of snake habitat preference, snake activity, and the seasonal patterns of farmers on different landcover types.

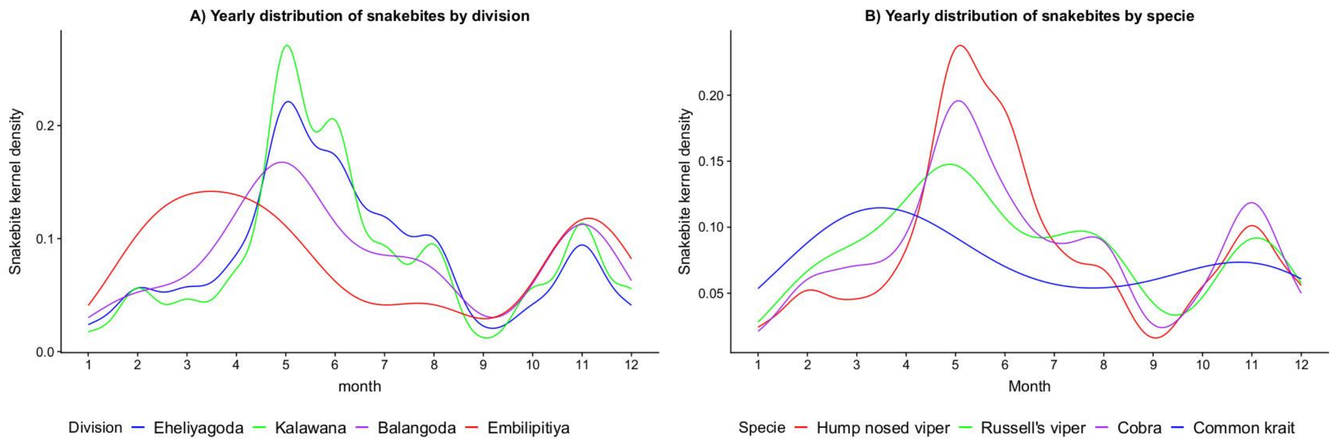

Figure 7: Secondary predictions A) the yearly distribution of snakebites for different divisions. Each division showed a distinct pattern of snakebite, with the largest peak of the year varying between March and May. **B) The yearly distribution of snakebites for different species**. Each species showed different snakebite peaks through the year, with the largest peak occurring between February and May.

Discussion

Snakebite affects poor and rural populations that are exposed to venomous snakes, yet few studies have attempted to decompose spatial and temporal patterns and predict risk on the basis of social-ecological causative mechanisms. Here we develop a mechanistic model to examine snakebite dynamics by simulating snake-human encounters in rural agricultural communities using an agent-based model (ABM). Our simulation represents the farmer-snake interactions that are driving snakebite patterns in Sri Lanka, a bite hotspot country within the highly affected South Asian region. While it has been previously shown that snakebites can have strong spatial and temporal patterns [11,36], and different studies have explored these patterns on local scales [58,59], our model provides a unique mechanistic perspective regarding the emergence of these patterns from basic ecological principals regarding species interactions on a more local scale. Results showed that the model performed well in simulating snakebite occurrences across spatial and temporal scales, including daily and seasonal patterns, biting species assemblages, and bite incidence variation among locations (Figure 4-6 and S1 Figure 1-7).

The results suggest that the risks of snakebite depend on factors influencing the behaviors of both farmers and snakes, including landcover, precipitation, and the interaction between humans and snakes (Figure 6-7). Our model also concurs with previous research showing that seasonal precipitation patterns dictate patterns of snakebites by influencing the activities of both snakes and farmers (Figure 6) [4,11]. We further discovered that different crop types result in distinct work schedule in relation to daily human activities and rainy seasons, greatly altering overall risk profiles of snakebites for each crop (Figure 6 E-G). Additionally, the composition of snake species is different among various crop types (SI 1 Figure 7), leading to complex social-ecological interactions that in turn contribute to snakebite risk [13].

Our model suggests greater resolution on the composition of species delivering bites is essential in order to better resolve snakebite risks in future (Figure 4). Previous research has supported the idea that following the ecology and behavior of each species would give a better understanding of both the mechanism driving bite patterns for individual snake species [17], and for different types of landcover (e.g.: [60]). Our model provides a mechanistic explanation for the ways snake ecology and human behavior combine to result in species specific snakebite patterns. For example, in our study system, although two species (Russell's vipers and Hump nosed vipers) show similar seasonal activity patterns, a stronger preference for rice paddies for one of the species (Russell's vipers) and a stronger preference for rubber plantations in the other species (Hump nosed vipers) results in very different temporal patterns of encounter. Understanding the overall pattern of snakebite therefore requires understanding of the specific ecology of each species (Figure 7B).

Such differences in an example of why predicted snakebite patterns vary considerably between locations, since spatial heterogeneity of famer types and snake species create fine scale differences in encounter risk, a prediction which concurs with previous research [11,12,36,61]. In our study, this difference between

locations was in practice caused by a combination of factors, including different distributions of key landcover types and climatic conditions, which in turn affect either snakes or famers or both. For example, the division of Embilipitiya, which is located in the intermediate climatic zone of Sri Lanka, had a less suitable environment for Hump-nosed vipers and a high concentration of rice paddies, resulting in a snakebite pattern different, including overall risk, temporal patterns of risk and biting species composition, to those found in the sites in the wet zones (Figures 4-5 & 7).

Broader applications of our approach are highly feasible. Since our model describes the socio-ecological mechanisms of snakebites rather than deriving correlative estimates of risk, it would be possible to re-parameterize the model with data from other regions to generate baseline snakebite risk predictions at any spatial or temporal scale pending availability of suitable data types. Other studies have already invoked similar mechanisms to explain observed patterns of risk in rural communities outside of Sri Lanka (e.g.: [13,16]), and as such our model has strong potential for applications in other areas across the tropics. For example, locations outside of Sri Lanka that include some of the same venomous snakes species have shown yearly temporal distributions of snakebites that contrast with those observed inside of Sri Lanka [15,16,62], which provides a strong avenue for hypothesis generation and testing of the model in different systems. Further af[illegible] other studies have similarly reported land-use specific risks (e.g. rubber in Liberia and rice in the Philippines) [63,64]. Transferring the model to these regions could shed further light on the combinations of factors that underpin different snakebite patterns among different locations, again a potentially fruitful avenue for hypothesis generation or validation.

While our model represented some of the most important snake behavior factors relevant to snakebite, there are other elements that we did not address, primarily due to data limitations. These include reproduction phenology and its association with climate [4], seasonal variability in landcover preferences [52], or species-specific feeding strategies. Similarly, we have not captured all the behavioral traits of farmers, such as differences in farming practices between small and large plantations, seasonal crop rotations [65], and additional crop types (e.g., small gardens, cinnamon, banana, coconut) [43], adaptive characteristics that represent farmers' planning strategies over multiple years, or specific behaviors relating to snakebite epidemiology, such as health seeking behavior or the use of protective measures (e.g., boots) [66]. Nevertheless, our model has demonstrated the importance of integrating both human and snake behavior into a single model and has shown that integrating even a few essential characteristics can have strong explanatory value for predicting patterns of snakebite.

Snakebite is an ongoing concern in Sri Lanka, and across southern Asia and much of the tropical and subtropical developing world. The World Health Organization has launched a strategic plan to reduce snakebite injuries and mortality by 50% by the year 2030, yet it has been suggested that one of the key barriers to preventing snakebite is the lack of good quality research to help direct effort [35]. Here we explored fine scale spatially explicit predictions by developing a novel mechanistic model to explain

snakebite risks based on snake behaviors (e.g. snake activities and distributions) and farmer behaviors (e.g. work schedules for different landcover types). Our approach is based on clear, general mechanisms and strong socio-ecological theory and is therefore highly transferrable to other systems, where the risks of snakebite are similarly associated with occupational characteristics, environmental conditions and snake ecological traits [8,16,18,67–69]. Our model, once implemented with local dataset, can examine the local socio-ecological drivers of snakebites and predict spatial and temporal snakebite patterns, as well as generating hypotheses and testing the efficacy of policy intervention. The insights gained in this study will help to focus future efforts to collect relevant data and resolve key mechanisms underlying snakebite risk, which should help advance management planning and the direction of scarce management resources

Bibliography

1. Gutiérrez, J. M., Williams, D., Fan, H. W., & Warrell DA. Snakebite envenoming from a global perspective : Towards an integrated approach. Toxicon. 2010;56: 1223–1235. doi:10.1016/j.toxicon.2009.11.020

2. Kasturiratne, A., Wickremasinghe, A.R., de Silva, N., Gunawardena, N.K., Pathmeswaran, A., Premaratna, R., Savioli, L., Lalloo, D.G. and de Silva HJ. The global burden of snakebite: A literature analysis and modelling based on regional estimates of envenoming and deaths. PLoS Med. 2008;5: 1591–1604. doi:10.1371/journal.pmed.0050218

3. Chippaux J. Snakebite envenomation turns again into a neglected tropical disease ! [illegible]nal Venom Anim Toxins Incl Trop Dis. 2017;23: 38. doi:10.1186/s40409-017-0127-6

4. Chaves LF, Chuang T, Sasa M, Gutiérrez JM. Snakebites are associated with poverty , weather fluctuations , and El Niño. Sci Adv. 2015;1.

5. Harrison RA, Hargreaves A, Wagstaff SC, Faragher B, Lalloo DG. Snake envenoming: A disease of poverty. PLoS Negl Trop Dis. 2009;3. doi:10.1371/journal.pntd.0000569

6. World Health Organization. Snakebite envenoming: A strategy for prevention and control. 2019.

7. Ediriweera DS, Diggle PJ, Kasturiratne A, Pathmeswaran A, Gunawardena NK, Jayamanne SF, et al. Evaluating temporal patterns of snakebite in Sri Lanka: The potential for higher snakebite burdens with climate change. Int J Epidemiol. 2018;47: 2049–2058. doi:10.1093/ije/dyy188

8. Rahman R, Faiz MA, Selim S, Rahman B, Basher A, Jones A, et al. Annual Incidence of Snake Bite in Rural Bangladesh. PLoS Negl Trop Dis. 2010;4: 1–6. doi:10.1371/journal.pntd.0000860

9. Murray, K.A., Martin G and TI. Focus on snake ecology to fight snakebite. Lancet. 2020;395: e14. doi:10.1016/S0140-6736(19)32510-3

10. Angarita-Gerlein, D, Bravo-Vega, CA, Cruz, C, Forero-Munoz, N.R., Navas-Zuloaga, M.G., Umana-Caro JD. Snakebite Dynamics in Colombia: Effects of Precipitation Seasonality on Incidence. Ires. 2017;2017: 1–4. Available: https://mcmsc.asu.edu/IRES

11. Ediriweera DS, Kasturiratne A, Pathmeswaran A, Gunawardena NK, Wijayawickrama BA, Jayamanne SF, et al. Mapping the Risk of Snakebite in Sri Lanka - A National Survey with Geospatial Analysis. PLoS Negl Trop Dis. 2016;10: 1–14. doi:10.1371/journal.pntd.0004813

12. Hansson E, Sasa M, Mattisson K, Robles A, Gutiérrez JM. Using Geographical Information Systems to Identify Populations in Need of Improved Accessibility to Antivenom Treatment for Snakebite Envenoming in Costa Rica. PLoS Negl Trop Dis. 2013;7. doi:10.1371/journal.pntd.0002009

13. Akani GC, Ebere N, Franco D, Eniang EA, Petrozzi F, Politano E, et al. Correlation between annual activity patterns of venomous snakes and rural people in the Niger Delta, southern Nigeria. J Venom Anim Toxins Incl Trop Dis. 2013;19: 1–8. doi:10.1186/1678-9199-19-2

14. Hansson E, Cuadra S, Oudin A, Jong K De, Stroh E, Tore K. Mapping Snakebite Epidemiology in Nicaragua – Pitfalls and Possible Solutions. PLoS Negl Trop Dis. 2010;4. doi:10.1371/journal.pntd.0000896

15. Mohapatra, B., Warrell, D.A., Suraweera, W., Bhatia, P., Dhingra, N., Jotkar, R.M., Rodriguez, P.S., Mishra, K., Whitaker, R., Jha P. Snakebite Mortality in India : A Nationally Representative Mortality Survey. PLoS Negl Trop Dis. 2011;5: 1–8. doi:10.1371/journal.pntd.0001018

16. Sharma SK, Koirala S, Dahal G, Sah C. Clinico-epidemiological features of snakebite : a study from Eastern Nepal. Trop Doct. 2004;34: 20–22.

17. Yañez-Arenas, C., Peterson, A. T., Mokondoko, P., Rojas-Soto, O., & Martínez-Meyer E. The Use of Ecological Niche Modeling to Infer Potential Risk Areas of Snakebite in the Mexican State of Veracruz. PLoS One. 2014;9. doi:10.1371/journal.pone.0100957

18. Yañez-arenas C, Peterson AT, Rodríguez-medina K, Barve N. Mapping current and future potential snakebite risk in the new world. Clim Change. 2016;134: 697–711. doi:10.1007/s10584-015-1544-6

19. Brown DG, Riolo R, Robinson DT, North M, Rand W. Spatial process and data models : Toward integration of agent-based models and GIS. 2005; 25–47. doi:10.1007/s10109-005-0148-5

20. Filatova T, Verburg PH, Cassandra D, Ann C. Environmental Modelling & Software Spatial agent-based models for socio-ecological systems : Challenges and prospects q. Environ Model Softw. 2013;45: 1–7. doi:10.1016/j.envsoft.2013.03.017

21. Parker DC, Manson SM, Janssen MA, Hoffmann MJ, Deadman P. Multi-agent systems for the simulation of land-use and land-cover change: A review. Ann Assoc Am Geogr. 2003;93: 314–337. doi:10.1111/1467-8306.9302004

22. Wilensky, U. and Rand W. An introduction to agent-based modeling: modeling natural, social, and engineered complex systems with NetLogo. MIT Press; 2015.

23. Bonabeau E. Agent-based modeling : Methods and techniques for simulating human systems. 2002;99.

24. Epstein, J.M. and Axtell R. Growing artificial societies: social science from the bottom up. Brookings Institution Press; 1996.

25. Deadman P, Robinson D, Moran E, Brondizio E. Colonist household decisionmaking and land-use change in the Amazon Rainforest: An agent-based simulation. Environ Plan B Plan Des. 2004;31: 693–709. doi:10.1068/b3098

26. Iwamura T, Lambin EF, Silvius KM, Luzar JB, Fragoso JMV. Agent-based modeling of hunting and subsistence agriculture on indigenous lands: Understanding interactions between social and ecological systems. Environ Model Softw. 2014;58: 109–127. doi:10.1016/j.envsoft.2014.03.008

27. An L, Liu J, Ouyang Z, Linderman M, Zhou S, Zhang H. Simulating demographic and socioeconomic processes on household level and implications for giant panda habitats. Ecol Modell. 2001;140: 31–49. doi:10.1016/S0304-3800(01)00267-8

28. Evans TP, Kelley H. Multi-scale analysis of a household level agent-based model of landcover change. J Environ Manage. 2004;72: 57–72. doi:10.1016/j.jenvman.2004.02.008

29. Lambin EF, Tran A, Vanwambeke SO, Linard C, Soti V. Pathogenic landscapes: Interactions between land, people, disease vectors, and their animal hosts. Int J Health Geogr. 2010;9: 1–13. doi:10.1186/1476-072X-9-54

30. Almeida SJ de, Martins Ferreira RP, Eiras ÁE, Obermayr RP, Geier M. Multi-agent modeling and simulation of an Aedes aegypti mosquito population. Environ Model Softw. 2010;25: 1490–1507. doi:10.1016/j.envsoft.2010.04.021

31. Thulke HH, Grimm V, Müller MS, Staubach C, Tischendorf L, Wissel C, et al. From pattern to practice: A scaling-down strategy for spatially explicit modelling illustrated by the spread and control of rabies. Ecol Modell. 1999;117: 179–202. doi:10.1016/S0304-3800(98)00198-7

32. Dion E, Lambin EF. Scenarios of transmission risk of foot-and-mouth with climatic, social and landscape changes in southern Africa. Appl Geogr. 2012;35: 32–42. doi:10.1016/j.apgeog.2012.05.001

33. Kasturiratne, A., Pathmeswaran, A., Wickremasinghe, A.R., Jayamanne, S.F., Dawson, A., Isbister, G.K., de Silva, H.J. and Lalloo DG, de Silva HJ, Lalloo DG. The socio-economic burden

of snakebite in Sri Lanka. PLoS Negl Trop Dis. 2017;11: 1–9. doi:10.1371/journal.pntd.0005647

34. De Silva A, Ranasinghe L. Epidemiology of snake-bite in Sri Lanka: a review. he Ceylon Med J. 1983;28: 144.

35. Ralph R. The timing is right to end snakebite deaths in south Asia. bmj. 2019;364: k5317. doi:10.1136/bmj.k5317

36. Kasturiratne A, Pathmeswaran A, M.M.D. F, Lalloo DG, Brooker S, Silva HJ De. Estimates of disease burden due to land-snake bite in Sri Lankan hospitals. Southeast Asian J Trop Med Public Health. 2005;36: 733–740. Available: http://www.embase.com/search/results?subaction=viewrecord&from=export&id=L41316804

37. Wilensky U. NetLogo. Evanston, IL: Center for connected learning and computer-based modeling, Northwestern University. 1999.

38. Macal CM, North MJ. Agent-based modeling and simulation. Proc 2009 Winter Simul Conf. 2009; 86–98. doi:10.1109/WSC.2009.5429318

39. Liu, J., Dietz, T., Carpenter, S.R., Alberti, M., Folke, C., Moran, E., Pell, A.N., Deadman, P., Kratz, T., Lubchenco, J. and Ostrom E. Complexity of coupled human and natural systems. Science (80-). 2007;317: 1513–1517. doi:10.1126/science.1144004

40. Wiegand T, Jeltsch F, Hanski I, Grimm V. Using pattern-oriented modeling for revealing hidden information : a key for reconciling ecological theory and application. Oikos. 2003;100: 209–222.

41. Grimm V, Revilla E, Berger U, Jeltsch F, Mooij WM, Railsback SF, et al. Complex Systems : Lessons from Ecology. Science (80-). 2005;310: 987–991.

42. Department of census and statistic Sri Lanka. Detail information on Tea in Sri Lanka. 2005.

43. Ministry of Plantation Industries Sri Lanka. Statistical Information on Plantation Crops. 20[illegible]

44. Supphiah R, Yoshino M. Some agroclimatological aspects of rice production in Sri Lanka. Geogr Rev Jpn. 1986;59: 137–153.

45. Olson, D. M., Dinerstein, E., Wikramanayake, E. D., Burgess, N. D., Powell, G. V. N., Underwood, E. C., D'Amico, J. A., Itoua, I., Strand, H. E., Morrison, J. C., Loucks, C. J., Allnutt, T. F., Ricketts, T. H., Kura, Y., Lamoreux, J. F., Wettengel, W. W., KR. Terrestrial ecoregions of the world: a new map of life on Earth. Bioscience. 2001;53: 933–938.

46. Hijmans RJ, Cameron SE, Parra JL, Jones G, Jarvis A. Very high resolution interpolated climate surfaces for global land areas. Int J Climatol A J R Meteorol Soc. 2005;25: 1965–1978. doi:10.1002/joc.1276

47. Zuhlke, M., Fomferra, N., Brockmann, C., Peters, M., Veci, L., Malik, J., & Regner P. SNAP (sentinel application platform) and the ESA sentinel 3 toolbox. Sentin Sci Work. 2015;Vol. 734.

48. Harris, I., Jones, P.D., Osborn, T.J. and Lister DH. Updated high-resolution grids of monthly climatic observations - the CRU TS3.10 Dataset. Int J Climatol. 2014;34: 623–642.

49. Mosier TM, Hill F, Sharp K V. 30-Arcsecond monthly climate surfaces with global land coverage. Int J Climatol. 2014;34: 2175–2188. doi:10.1002/joc.3829

50. Domroes M, Ranatunge E. A statistical approach towards a regionalization of daily rainfall in Sri Lanka. Int J Climatol. 1993;13: 741–754. doi:10.1002/joc.3370130704

51. Martin G. Unpublished. 2019.

52. Sawant NS, Jadhav TD, Shyama SK. Distribution and abundance of pit vipers (Reptilia: Viperidae) along the Western Ghats of Goa, India. J Threat Taxa. 2013;2: 1199–1204. doi:10.11609/jott.o2489.1199-204

53. França, Frederico Gustavo Rodrigues and V da SB. Diversity , activity patterns , and habitat use of the snake fauna of Chapada dos Veadeiros National Park in Central Brazil. Biota Neotrop. 2013;13: 74–84.

54. Rahman. Monsoon does matter. annual activity patterns in a snake. Herp J 23(4). 2013;23: 203–208.

55. Rocha CFD, Bergallo HG, Vera y Conde CF, Bittencourt EB, Santos H de C. Richness, abundance, and mass in snake assemblages from two Atlantic Rainforest sites (Ilha do Cardoso, São Paulo) with differences in environmental productivity. Biota Neotrop. 2008;8: 117–122. doi:10.1590/s1676-06032008000300011

56. de Silva A. Colour guide to the snakes of Sri Lanka. R & A. Portishead, UK: R & A Publishing Ltd.; 1990. doi:10.1016/0041-0101(91)90090-e

57. Grimm V, Railsback SF. Pattern-oriented modelling: A “multi-scope” for predictive systems ecology. Philos Trans R Soc B Biol Sci. 2012;367: 298–310. doi:10.1098/rstb.2011.0180

58. De Silva A. Snakebites in Anuradhapura district. The Snake. 1981;13: 117–130.

59. Farooqui JM, Mukherjee BB, Manjhi SNM, Farooqui AAJ, Datir S. Incidence of fatal snake bite in Loni, Maharashtra: An autopsy based retrospective study (2004-2014). J Forensic Leg Med. 2016;39: 61–64. doi:10.1016/j.jflm.2016.01.013

60. Kularatne SAM, Silva A, Weerakoon K, Maduwage K. Revisiting Russell ’ s Viper (Daboia russelii) Bite in Sri Lanka : Is Abdominal Pain an Early Feature of Systemic Envenoming ? PLoS One. 2014;9: 1–8. doi:10.1371/journal.pone.0090198

61. Molesworth AM, Harrison R, Theakston RDG, Lalloo DG. Geographic information system mapping of snakebite incidence in northern Ghana and Nigeria using environmental indicators: A preliminary study. Trans R Soc Trop Med Hyg. 2003;97: 188–192. doi:10.1016/S0035-9203(03)90115-5

62. Kumar S. Clinical and epidemiologic profile and predictors of outcome of poisonous snake bites – an analysis of 1 , 500 cases from a tertiary care center in. Int J Gen Med. 2018;11: 209–216.

63. Stahel E. Epidemiological aspects of snake bites on a Liberian rubber plantation. Acta Trop. 1980;37: 367–374.

64. Watt, G., Padre, L., Tuazon, M. L., & Hayes CG. Bites by the Philippine Cobra (Naja naja philippinensis): an Important Cause of Death among Rice Farmers. Am J Trop Med Hyg. 1987;37: 636–639.

65. Food and Agriculture Organization of the United: regional office for Asia and The Pacific. Crop diversification in the Asia-Pacific region. 2001.

66. Silva, A., Marikar, F., Murugananthan, A. and Agampodi S. Awareness and perceptions on prevention , first aid and treatment of snakebites among Sri Lankan farmers : a knowledge practice mismatch ? J Occup Med Toxicol. 2014;9: 20.

67. Albuquerque HN, Fernandes A, Albuquerque ICS. Snakebites in Paraíba, Brazil. J Venom Anim Toxins Incl Trop Dis. 2005;11: 242–251. doi:10.1590/s1678-91992005000300003

68. Inamdar IF, Aswar NR, Ubaidulla M, Dalvi SD. Snakebite : Admissions at a tertiary health care centre in. S Afr Med J. 2010;100: 456–458.

69. Zacarias D, Loyola R. Climate change impacts on the distribution of venomous snakes and snakebite risk in Mozambique. Clim Change. 2019;152: 195–207. doi:10.1007/s10584-018-2338-4

Supporting information 1 – Additional Results

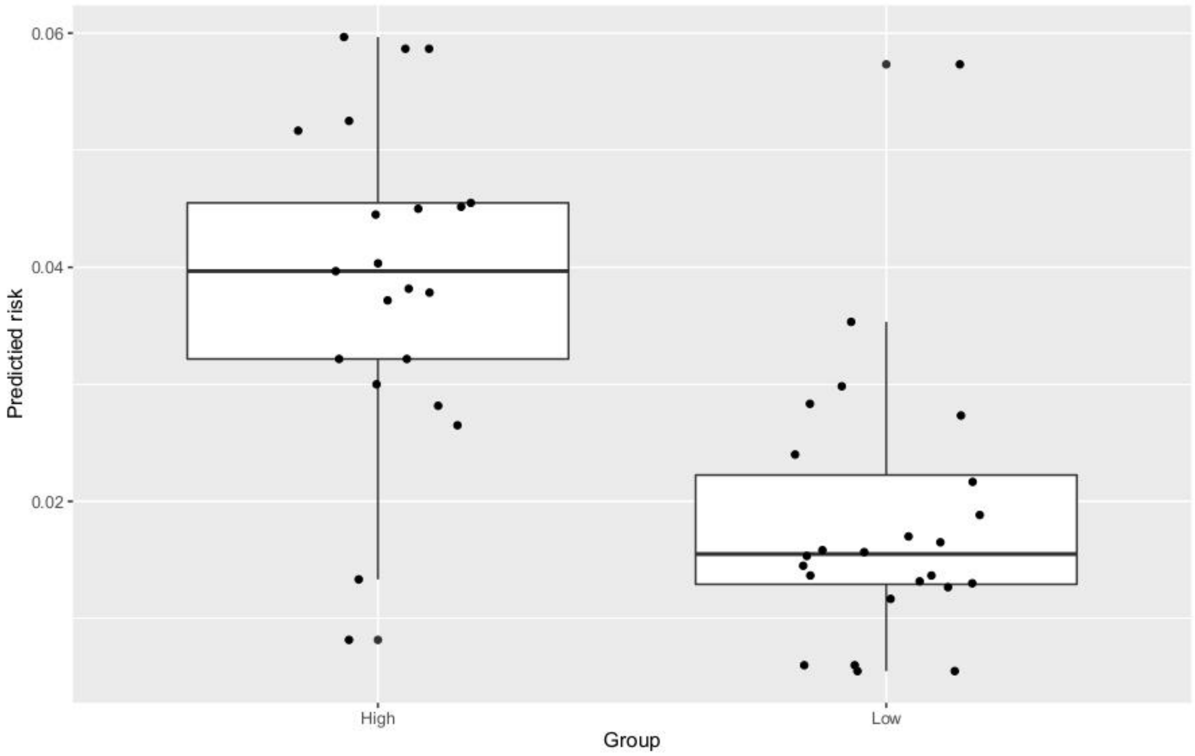

Figure 1: Model output of mean snakebite risk for locations with high and low snakebite occurrence values estimated by Ediriweera et al 2016. Results are based on 30 simulation runs for 45 different locations across the entire district of Ratnapura, with high and low defined as above or below the median snakebite risk for all locations.

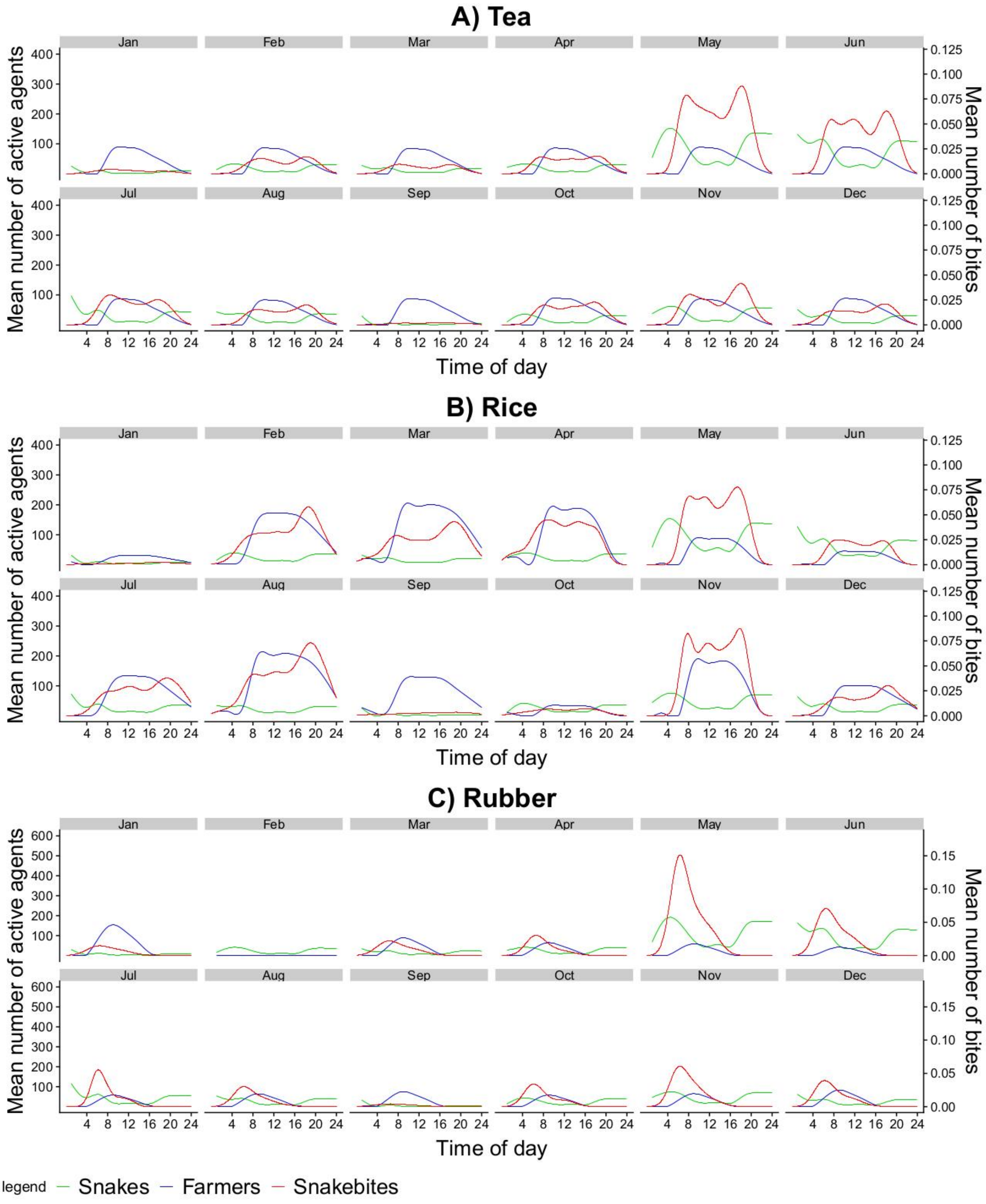

Figure 2: The daily spatial temporal overlap of farmers and snakes. The values are an average of 660 simulation runs. Each graph follows the daily spatio-temporal overlap between farmers and snakes that cause the emergence of snakebites patterns for **A)** tea **B)** rice, and **C)** rubber.

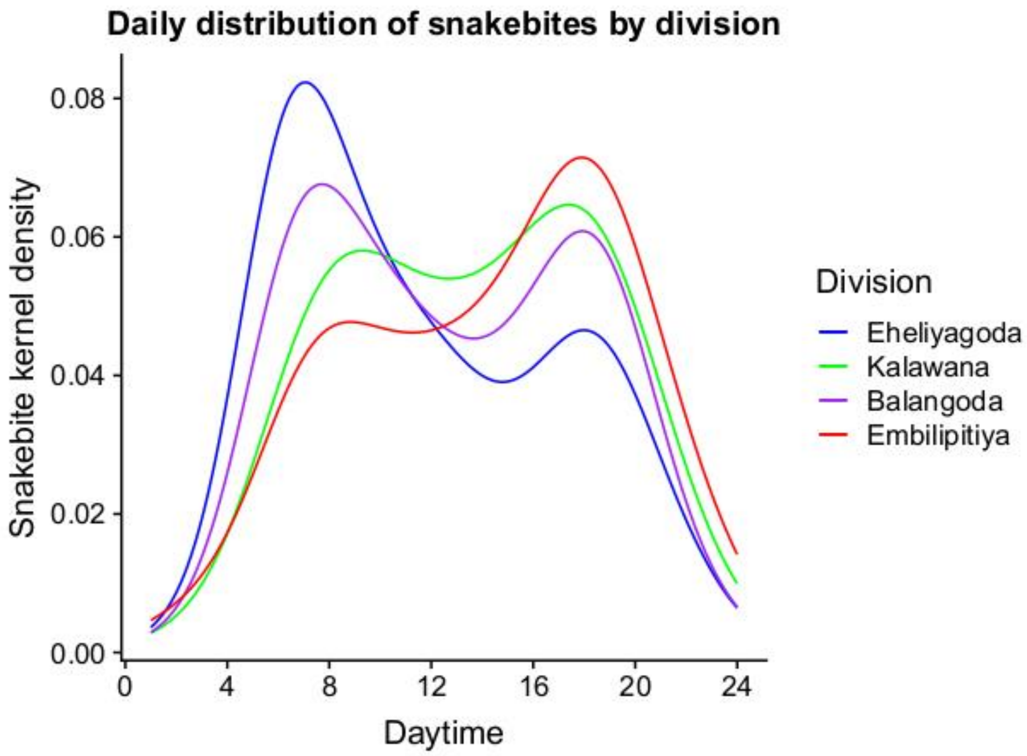

Figure 3: A secondary prediction of bitting hour between different divisions. our model shows that different divisions are going to have different daily distributions of snakebites.

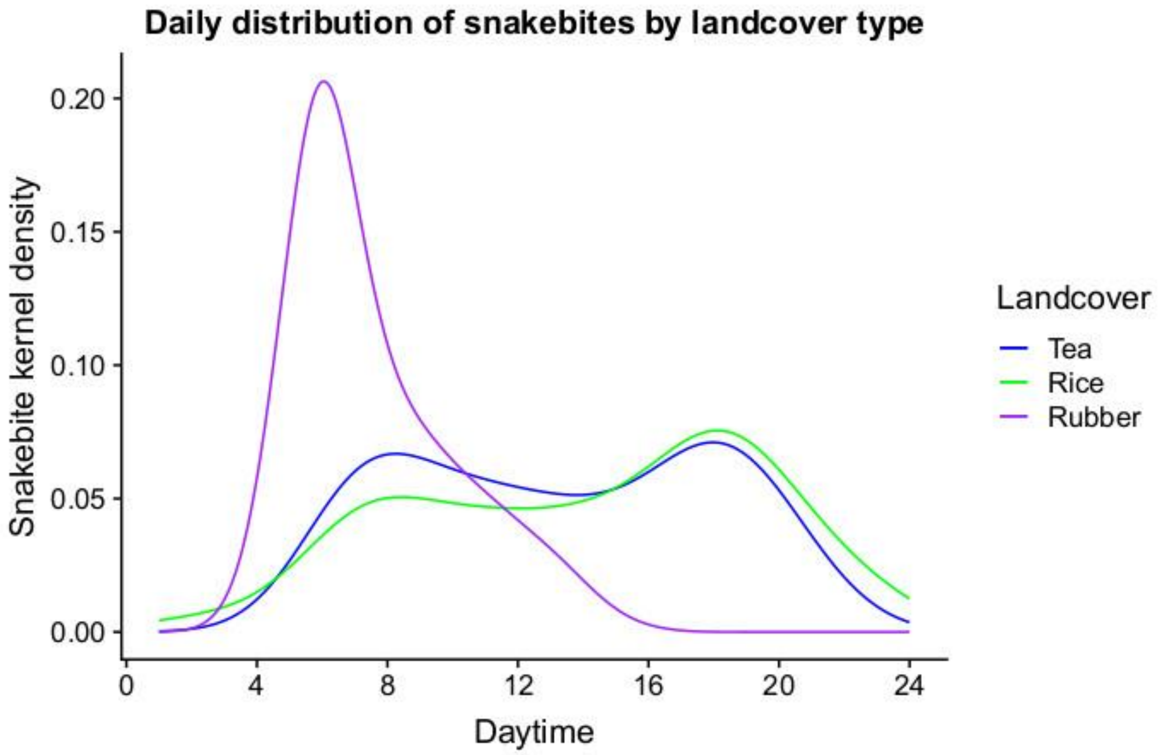

Figure 4: A secondary prediction of snakebite occurrence hour in different landcover types. our model shows that different landcover types are going to have different daily distributions of snakebites across the hours of the day.

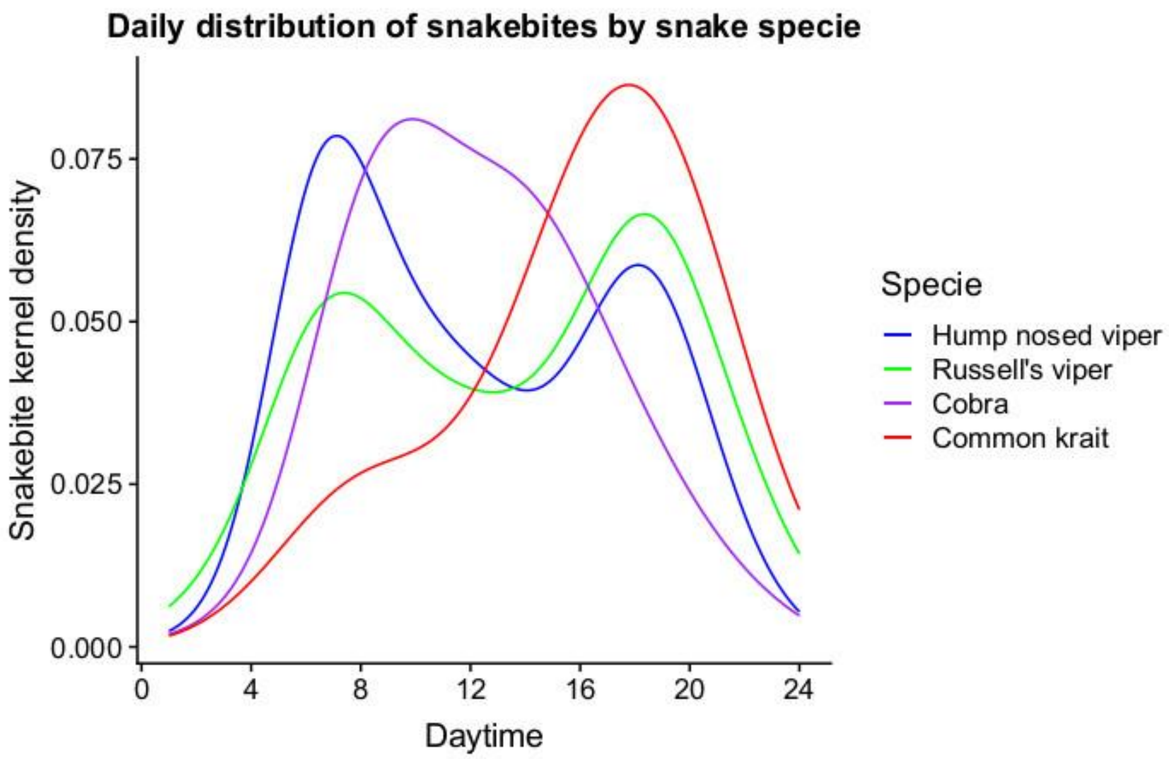

Figure 5: A secondary prediction of species biting hour. Our model shows that different species of snakes are going to have different distributions of snakebites across the day.

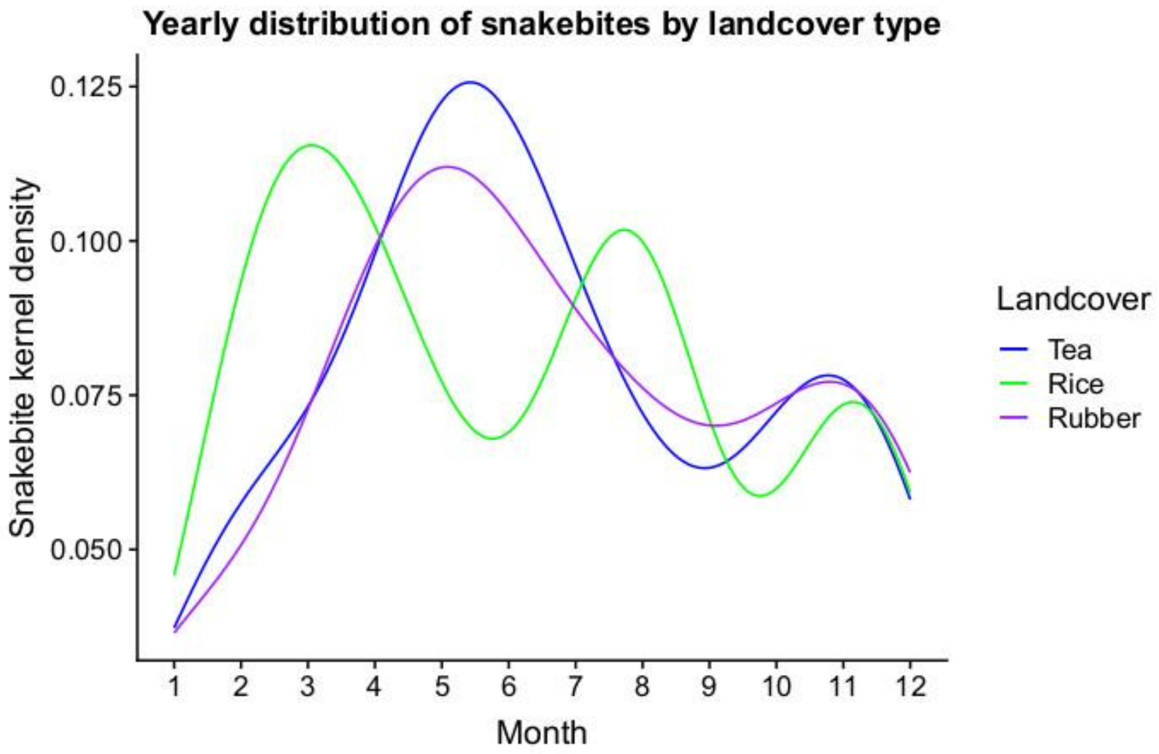

Figure 6: A secondary predictions monthly distribution of bites by landcover. Our model shows that the different landcovers are going to have different patterns of snakebites distribution across the year.

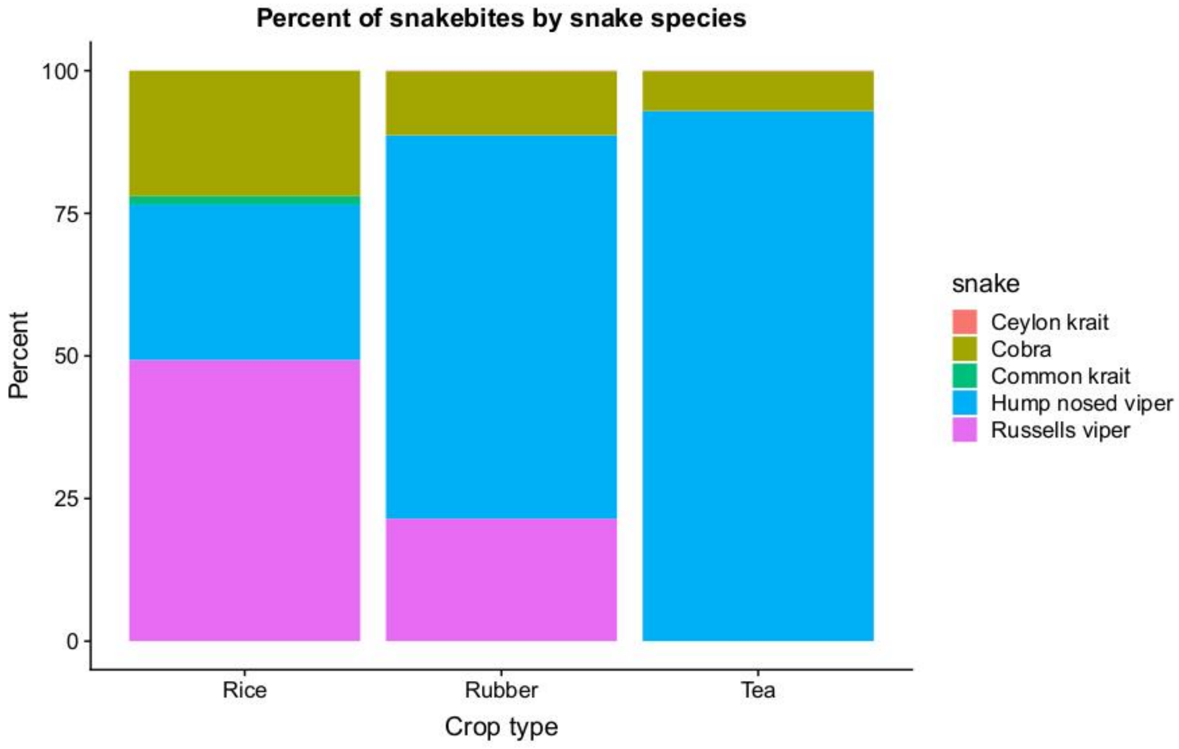

Figure 7: A secondary predictions of species biting on each landcover. Our model shows that the different landcovers are going to have different compositions of snake species causing snakebites.

Supporting information 2 – Supporting Materials

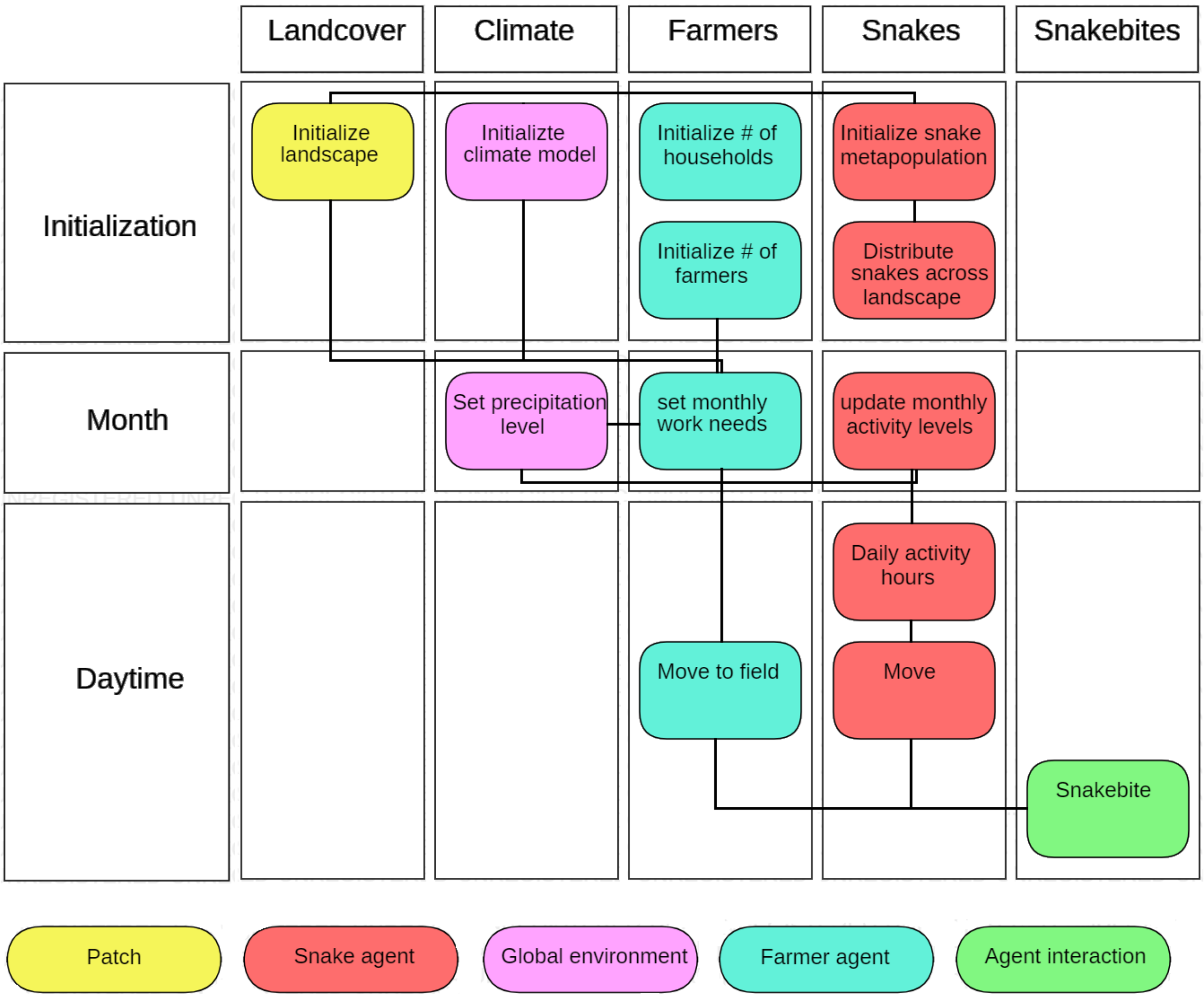

Figure 1: Model outline. Columns represent the different subprocesses in the model, rows represent the different units of scheduling, and colours represent the different agents and inputs/outputs of the model. Lines between boxes represent the relationship between the agents and variables. Climate and landcover affect the agents' behaviours across space and time, and this generates the spatio-temporal overlap between snake and humans that drive snakebite patterns.

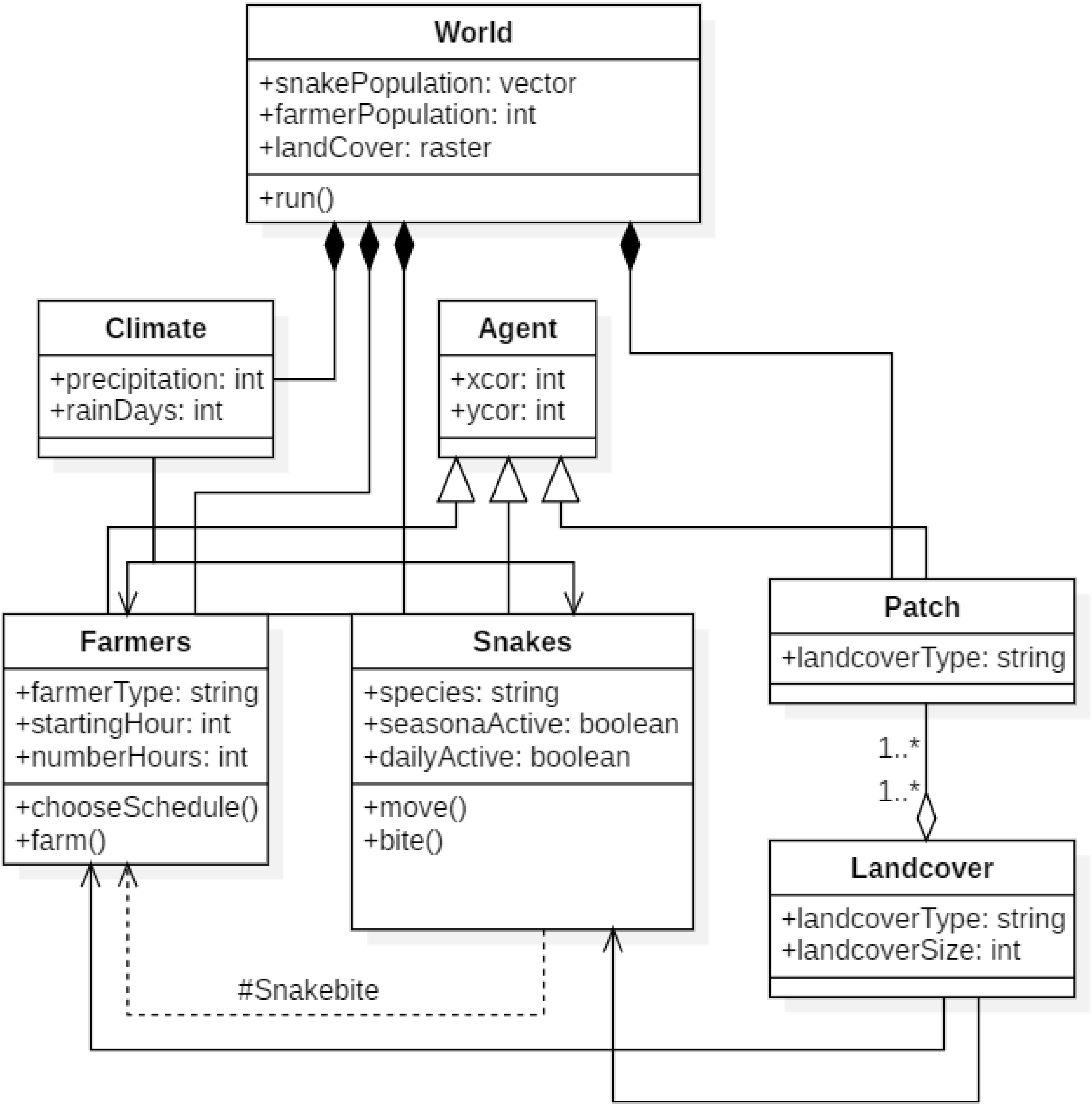

Figure 2: Model structure. The model structure represented through UML class diagram. The attributes and operations of each class are simplified in order to represent the entire process and the relations between classes.

Overall Accuracy = (53684/64481) 83.2555%

Kappa Coefficient = 0.6847

Class	Commission	Omission	Commission	Omission
	(Percent)	(Percent)	(Pixels)	(Pixels)
Rubber	43.58	87.05	659/1512	5732/6585
Water	0.59	0.00	43/7265	0/7222
Forest	16.43	3.68	7556/45990	1469/39903
Rice	13.39	29.14	654/4885	1740/5971
Tea	39.03	38.67	1885/4829	1856/4800

Class	Prod. Acc.	User Acc.	Prod. Acc.	User Acc.
	(Percent)	(Percent)	(Pixels)	(Pixels)
Rubber	12.95	56.42	853/6585	853/1512
Water	100.00	99.41	7222/7222	7222/7265
Forest	96.32	83.57	38434/39903	38434/45990
Rice	70.86	86.61	4231/5971	4231/4885
Tea	61.33	60.97	2944/4800	2944/4829

Table 1: accuracy assessment for support vector machine classifications

Overall Accuracy = (52041/64481) 80.7075%

Kappa Coefficient = 0.6664

Class	Commission	Omission	Commission	Omission
	(Percent)	(Percent)	(Pixels)	(Pixels)
Rubber	57.38	81.97	1598/2785	5398/6585
Water	0.12	0.00	9/7231	0/7222
Forest	11.86	10.88	4783/40344	4342/39903
Rice	14.34	33.96	660/4603	2028/5971
Tea	56.63	14.00	5390/9518	672/4800

Class	Prod. Acc.	User Acc.	Prod. Acc.	User Acc.
	(Percent)	(Percent)	(Pixels)	(Pixels)
Rubber	18.03	42.62	1187/6585	1187/2785
Water	100.00	99.88	7222/7222	7222/7231
Forest	89.12	88.14	35561/39903	35561/40344
Rice	66.04	85.66	3943/5971	3943/4603
Tea	86.00	43.37	4128/4800	4128/9518

Table 2: accuracy assessment for maximum likelihood classifications

Month	Rice	Tea	Rubber
Jan	12.16	80.8	5.2*R
Feb	72.96	80.8	0
Mar	99.39	80.8	5.2*R
Apr	84.5	80.8	5.2*R
May	33.8	80.8	5.2*R
Jun	16.9	80.8	5.2*R
Jul	52.2	80.8	5.2*R
Aug	110.06	80.8	5.2*R
Sep	50.28	80.8	5.2*R
Oct	13.26	80.8	5.2*R
Nov	79.68	80.8	5.2*R
Dec	38.72	80.8	5.2*R

Table 3: The mean index value for labour needed to farm a square kilometre of each one of the landcover types. The index value for rice varies through the year and is based on both the labour needed and the likelihood to work on the landcover at a specific month. The mean value for tea is equal between all months since seasonality doesn't affect labour in tea plantations. The value for rubber is dependent on the mean value of the index and on R which represents number of non-rainy days during a specific month.

Hours	Rice	Tea	Rubber
3	0	0	1
4	1	1	0
5	1	1	2
6	0	3	2
7	1	2	3
8	1	8	0
9	5	2	0
10	1	3	1
11	5	1	0
12	2	0	0
13	1	1	0
14	0	0	0
15	0	0	0
16	0	0	0
17	1	0	0
18	1	0	0

Table 4: The table represents the different possible number of hours worked on the different landcover types as reported by farmers during our field work.

Hours	rice	tea	rubber
4	0	0	1
5	0	1	4
6	5	2	3
7	10	12	1
8	4	6	0

Table 5: The table represents the different possible start hours for the different landcover types as reported by farmers during our field work.

Specie	Forest	Rubber	Rice	Home	Tea
Hypnale hypnale	0.929	0.976	0.348	2.472	1.387
Naja naja	.653	.895	1.352	5.209	.387
Daboia russelii	0.573	0.953	1.440	5.599	0
Bungarus ceylonicus	0.825	1.087	0	24.92	1.27
Bungarus caeruleus	0.722	1.012	1.289	4.277	0
Trimeresurus trigonocephalus	1.051	0.947	0.235	2.269	0.777
Echis carinatus	0.391	0.408	2.429	4.585	0

Table 6: The land association factor between different snake species and landcover types, based on Martin et al. 2019 unpublished.

Supporting information 3 – Technical Evaluation

Model selection:

For model selection we removed models that gave unrealistic representations of the system we are modeling, including non-realistic proportions of snakebites between species and unlikely months for snakebite peaks. If a certain mathematical formulation of our model caused the loss of a pattern that was previously observed on the macro level, then it was not used for later analysis.

We checked the structure of the model by comparing different relationships between variables and outcomes. More specifically, we checked for the possible relationship between the aggressiveness index and the propensity of snakes to bite, and the different possible relationships between precipitation and snake activity. This allowed us to make sure our modeling method was sensible before checking for the sensitivity of our model to changes in variable magnitude. The different relationships were observed through the yearly distribution of bites, the daily distribution of bites, the attack assemblage of species causing the bites, and the total number of bites.

1. The aggressiveness index was collected as ordinal data, a ranking of the propensity to bite of the different snake species. For this index, in addition to the linear relationship between the aggressiveness index and the propensity to bite as described above, we checked two additional mathematical relationships: a concave relationship represented by $\sqrt{aggressiveness}$, where snakes with a low score would be more affected by the index while snake species with high scores would show similar behavior; and a convex relationship represented by $(\text{aggressiveness})^2$, where snakes with a high score would be more affected by the index and snakes with low scores would show more similar behavior.
2. We also checked three different relationships between precipitation and snake activity, a linear relationship as described above, where snake activity is directly proportional to precipitation; a concave (decelerating) relationship represented by $\sqrt{\frac{precipitation}{precipitation_{max}}}$, and a convex (accelerating) relationship that was represented by $(\text{precipitation}/\text{precipitation}_{\text{max}})^2$.

Sensitivity analysis

We then conducted a sensitivity analysis against four different variables for which we had insufficient data or no data at all. Our sensitivity analysis helped identify which variables were the most influential on the simulation output. During the sensitivity analysis we used a linear relationship between the aggressiveness index and the propensity to bite for the sake of simplicity and saving computation time.

The variables used for the sensitivity analysis were:

1. After the model selection step, we chose to conduct additional sensitivity analysis for both a linear relationship and a concave relationship between precipitation and snake seasonal activity, but not for a convex relationship because it produced unrealistic patterns in the model selection step. For the two possibilities we checked the effects of different strengths of association between the precipitation and activity as:
    a. $(\text{precipitation} / \text{precipitation}_{max})^x$ with x = 0.1, 0.25, 0.5, for the concave relationship.
    b. $(x + \text{precipitation})/(x + \text{precipitation}_{max})$, with x = 100, 500, 900 For the linear relationship.
2. We originally defined the baseline dial activity levels of snakes with a probability of being active at p = 0.1. For the sensitivity analysis we checked a baseline probability of activity with a value of p = 0, 0.2 and 0.3 for all species except for cathemeral snakes, which were kept at a probability of 0.1 across all times of day.
3. The labour index value was collected during the field work and represented the expected number of people working in a 1 km^2 area for each landcover. This index was used as an input for the algorithm that decides how farmers allocated their time to different land cover types according to seasonal needs. For the sensitivity analysis we checked the lowest and the highest value of the index.
4. Since our snake population size was calibrated using previous research, for the sensitivity analysis we checked for different population sizes. We changed the factor that was used for scaling up the PPM models by values that ranged between $1x10^{10}$ and $9x10^{10}$.

The results of the sensitivity analyses were monitored with several different model outputs: frequency of bites in different landcovers, frequency of bites across snake species, daily distribution of bites, monthly distribution of bites, number of bites per location, and total number of bites per simulation run.

Results

The most-probable relationships between the aggressiveness index and the propensity of snakes to bite were convex and linear, based on the aggressiveness index (see Figures C.1-4). The most probable relationships between snake seasonal activity and rainfall were linear and concave (decelerating) (see Figures C.5-8).

In the sensitivity analysis, for the precipitation signal strength we found a threshold value with which a yearly pattern of snakebites is transformed (see Figures C.10, C.14). For population size a significant threshold was observed when the PPM models were scaled up by a value of $1x10^{10}$ under which our model no longer showed any significant pattern (see Figures C.26). For the labour index and the baseline activity, we kept the values as they were originally defined (see Figure C.17-24), since we did not find any significant thresholds.

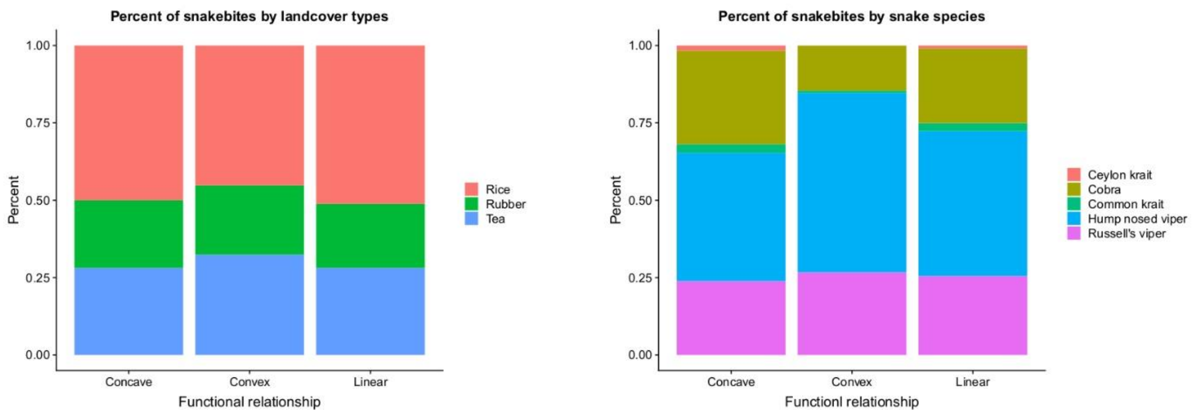

Figure 1: Our simulation was executed 30 times for each functional relationship at each one of the locations modelled. Changing the functional relationships for the aggressiveness index and propensity to bite had only a small effect on the percentage of snakebites occurring on each landcover type, but had a large effect on the percentage of bites caused by each one of the species, with a convex functional relationship showing an increased number of Hump nosed viper bites, and a decreased number of Cobra bites.

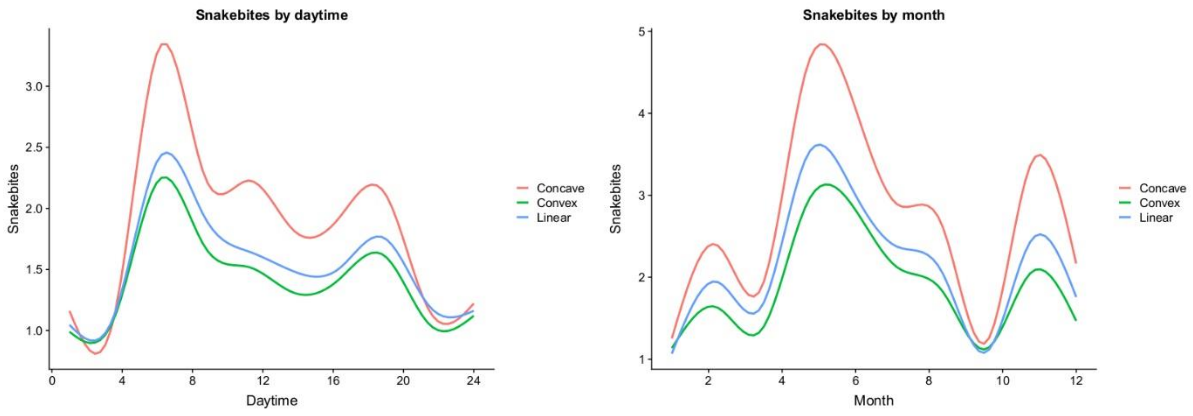

Figure 2: Changing the functional relationships between the aggressiveness index and the propensity to bite had an effect on the daily distribution of snakebites, with the early morning snakebite peak becoming much large when a concave relationship was defined. For the yearly distribution of snakebites there was change in magnitude but not in pattern.

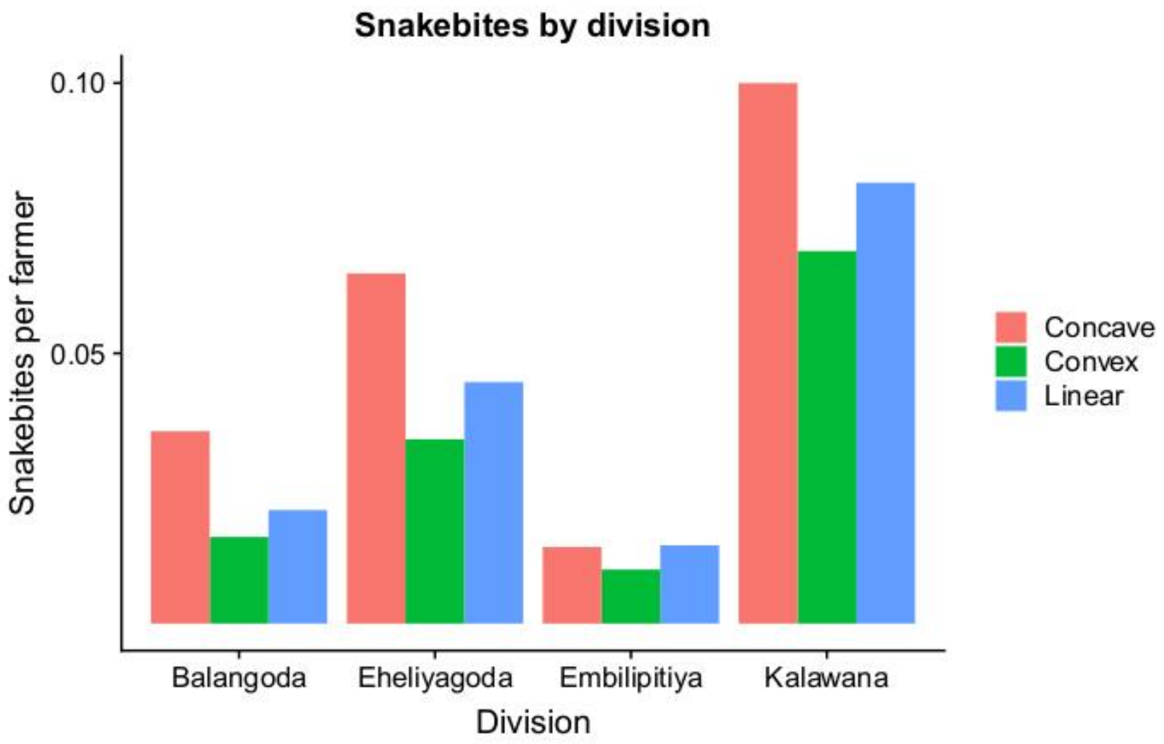

Figure 3: Changing the functional relationships between the aggressiveness index and propensity to bite had a only a moderate effect on the geographical patterns of snakebites, where each one of the divisions showed similar patterns when the functional relationship changed.

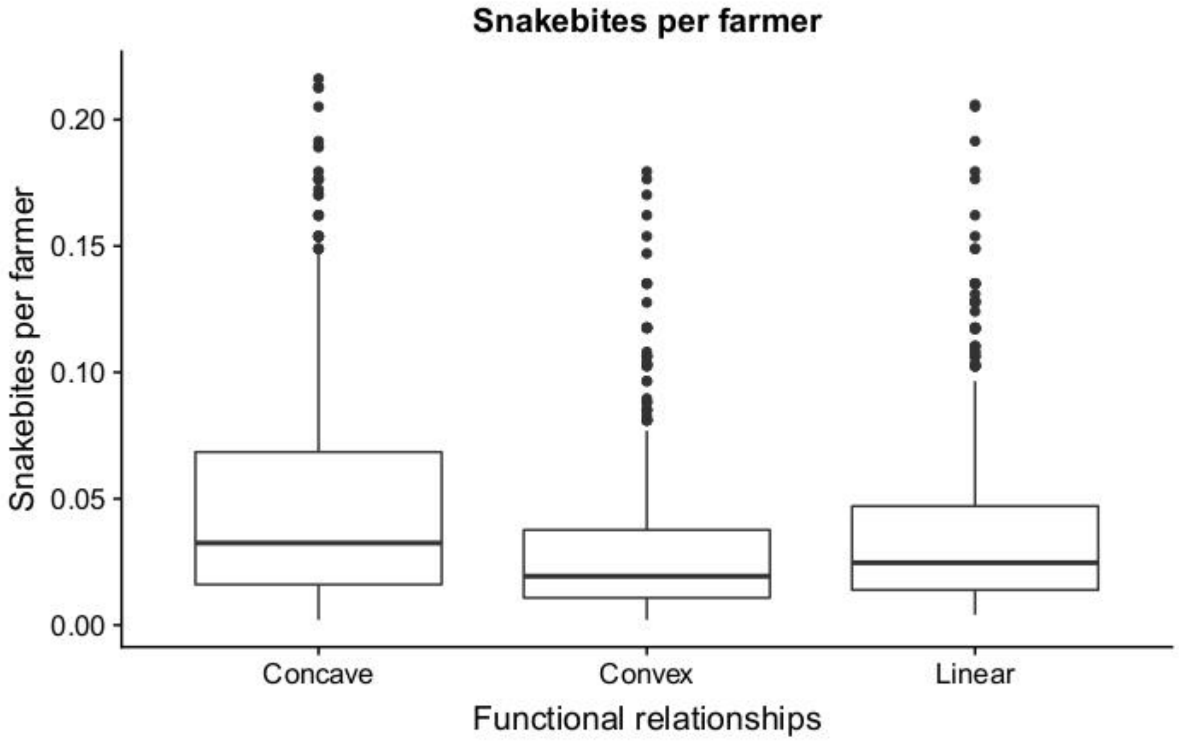

Figure 4: Changing the functional relationships had some effect on the total number of bites, with the mean number of snakebites remaining similar, but for the convex relationships there was a lower mean and less variance in comparison with the linear, and concave.

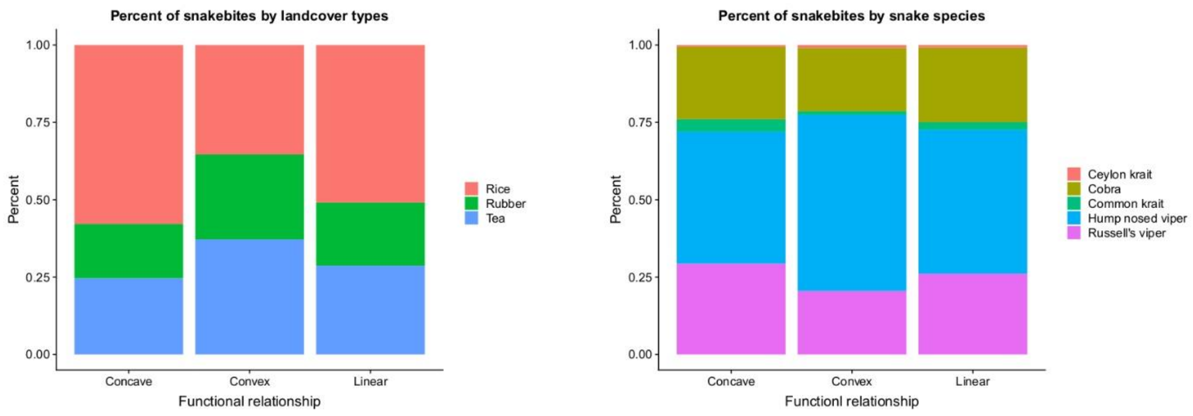

Figure 5: Our simulation was executed 30 times for each functional relationship at each one of the locations modelled. Changing the functional relationships influenced the percentage of snakebites occurring on each landcover type with the convex relationship causing less snakebites on rice paddies and more snakebites on rubber plantations. There was also an effect on the percentage of bites caused by each one of the species, with a convex functional relationship showing an increased number of Hump nosed viper bites, and fewer Russell's viper bites.

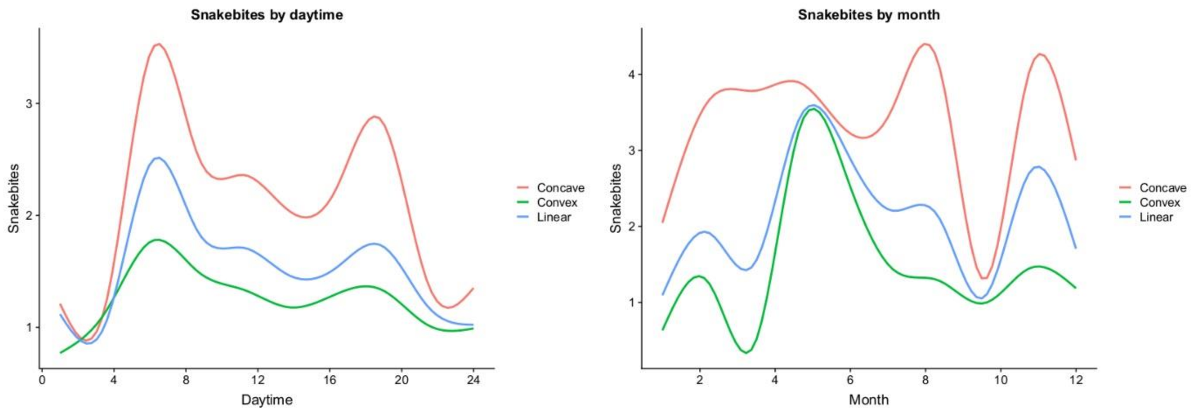

Figure 6: Changing the functional relationships influenced the distribution of bites both on the daily level and on the monthly level. On the daily level there was only a change in magnitude, with the concave relationship showing a higher number of snakebites through the different hours of the day. On the monthly level there was also a change in pattern. While the linear and convex relationships showed similar patterns across the year, the concave relationship showed a different patter, with a much larger peak between February and May, and a second large snakebite peak at the month of August.

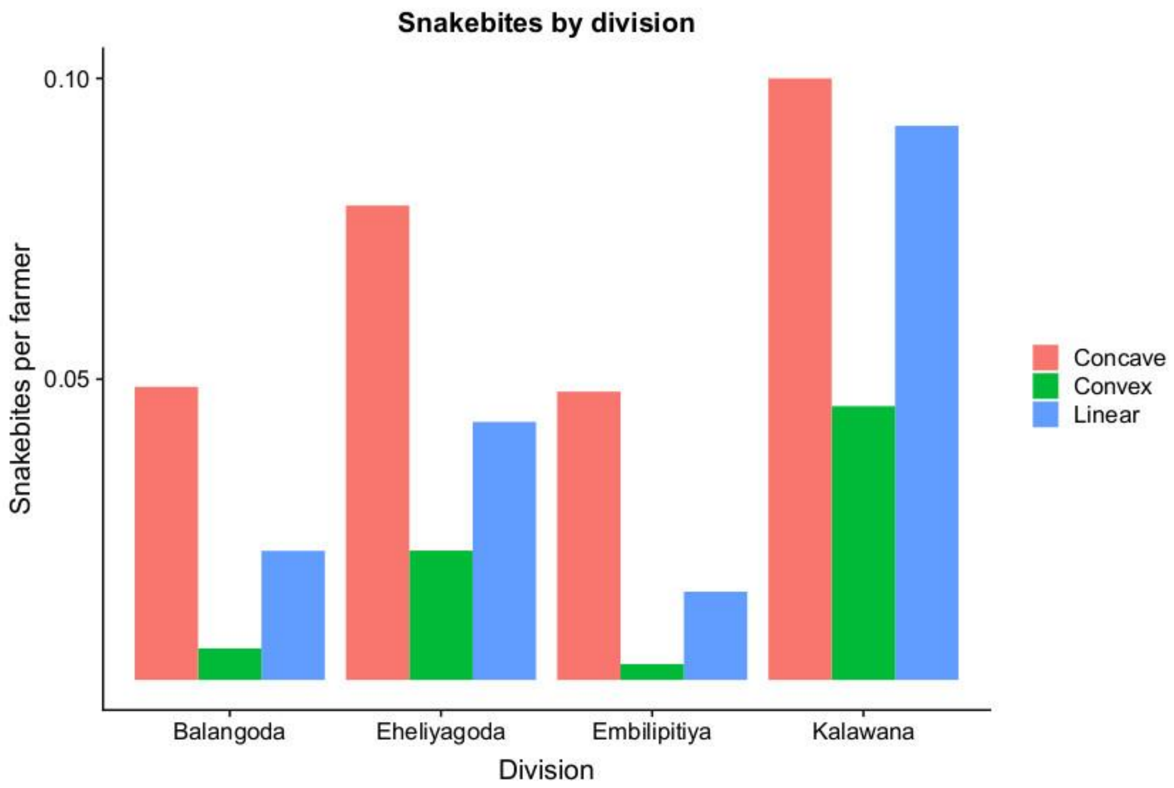

Figure 7: Changing the functional relationships between precipitation and snake seasonal activity levels influenced the different locations modelled at the different divisions in different ways, meaning that the different functional relationships had an effect on the geographical distribution of snakebites.

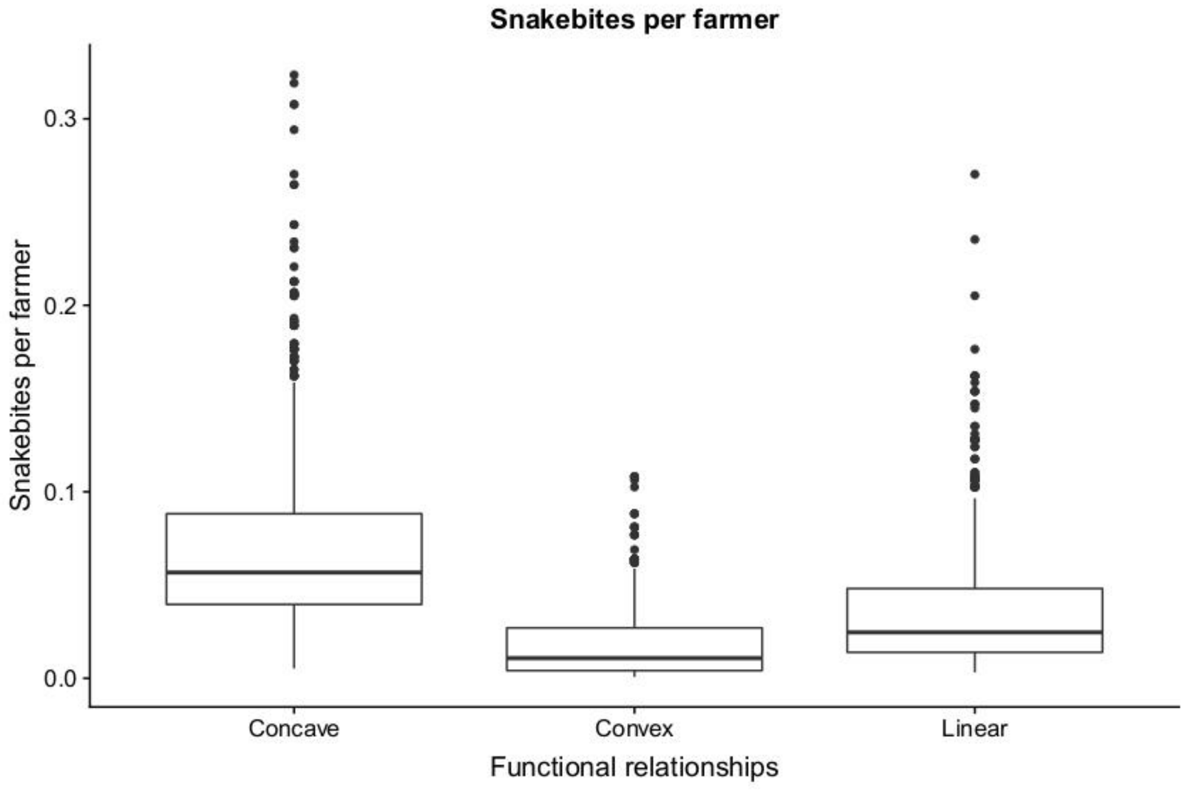

Figure 8: Changing the functional relationships between precipitation and the snake seasonal activity influenced the total number of snakebites. The convex relationship showed a smaller mean of snakebites across different simulations, and less variation in the number of snakebites as well. The concave relationship showed a high mean than the other two, and much more uncertainty in model outcomes.

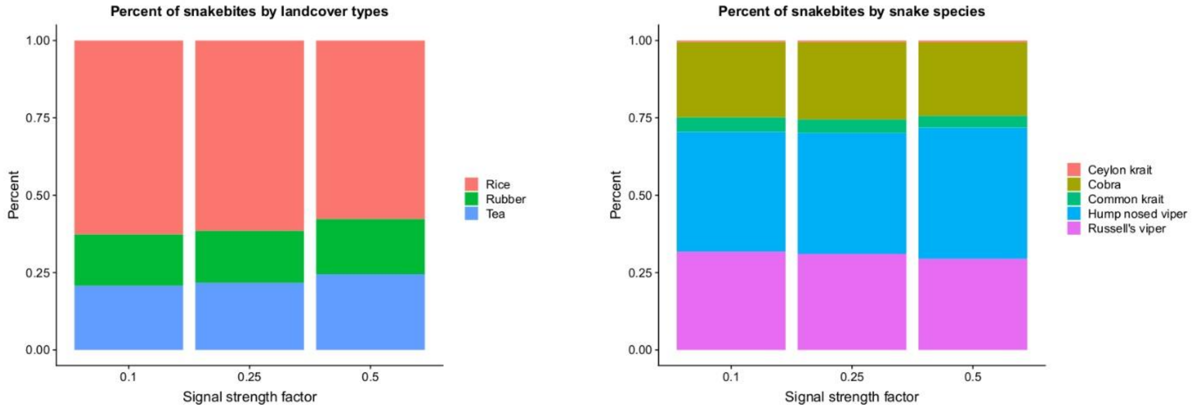

Figure 9: Our simulation was executed 30 times for each relationship intensity at each one of the locations modelled according to the following posibilites: $(\text{precipitation} / \text{precipitation}_{max})^x$ with x = 0.1, 0.25, 0.5,. The different signal strengths between a concave precipitation function precipitation and snake seasonal activity had only a small effect on the percentage of snakebites occurring on each landcover type, and the percentage of snakebites caused by each one of the snake species.

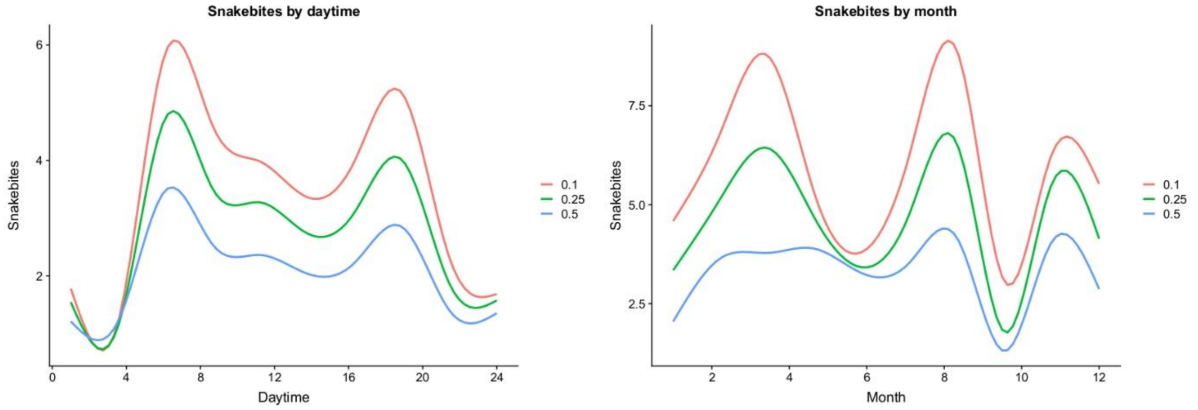

Figure 10: The different relationships intensities between a concave precipitation function and snake seasonal activity had a strong effect on the temporal distribution of snakebites. On the daytime level the differences only amounted to change in magnitude as the signal was strengthened. On the monthly level we observed change in pattern as well, when after a certain reduction in signal strength we lose some of the distinct yearly snakebite patterns such as a snakebite peak between March and May.

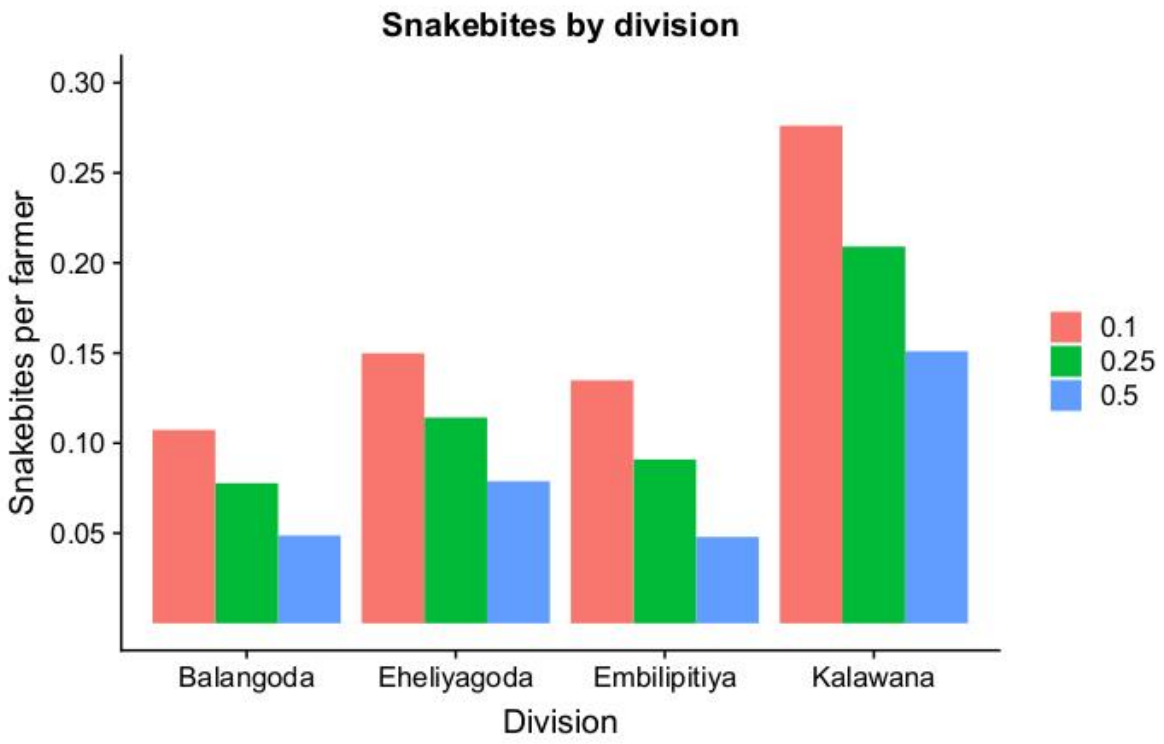

Figure 11: The different relationship intensities between a concave precipitation function and snake seasonal activity tended to effect different regions in similar ways. An increase in intensity of signal had the same effect regarding number of snakebites across all locations that we modelled.

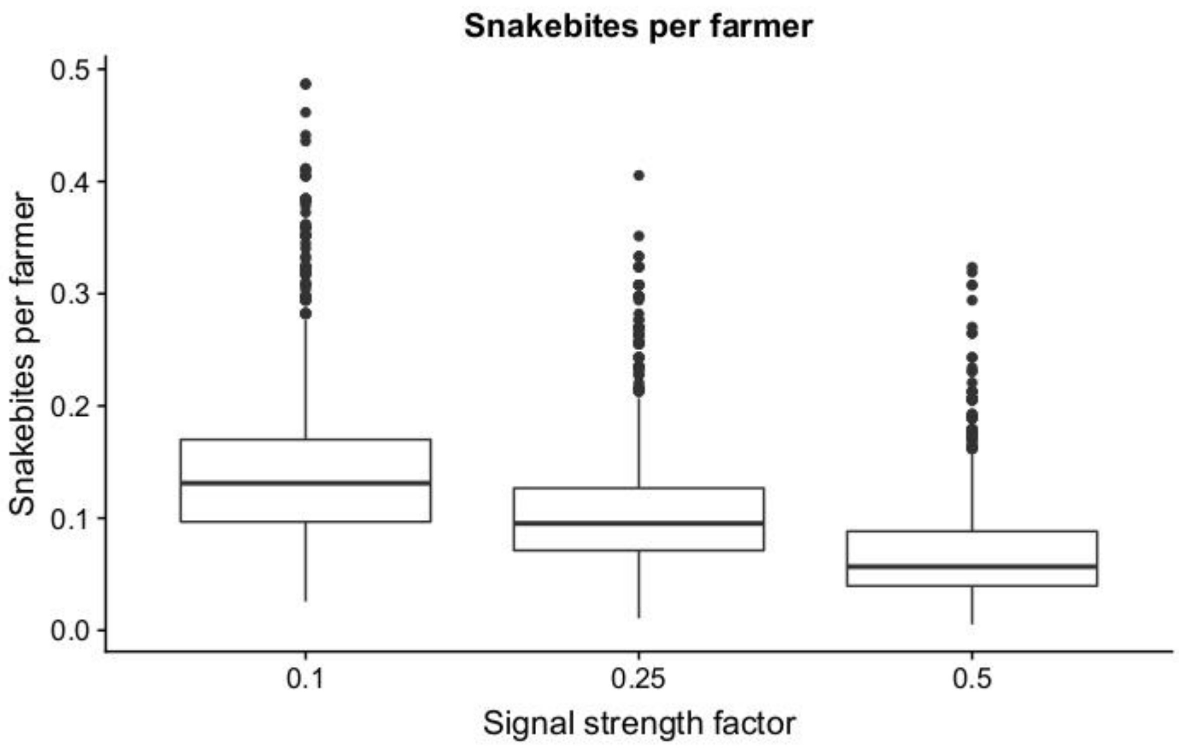

Figure 12: The different relationship intensities between a concave precipitation function and snake seasonal activity showed that an increase in intensity causes an increase in total number of snakebites. The distribution of snakebites around the mean remained relatively similar regardless of the intensities.

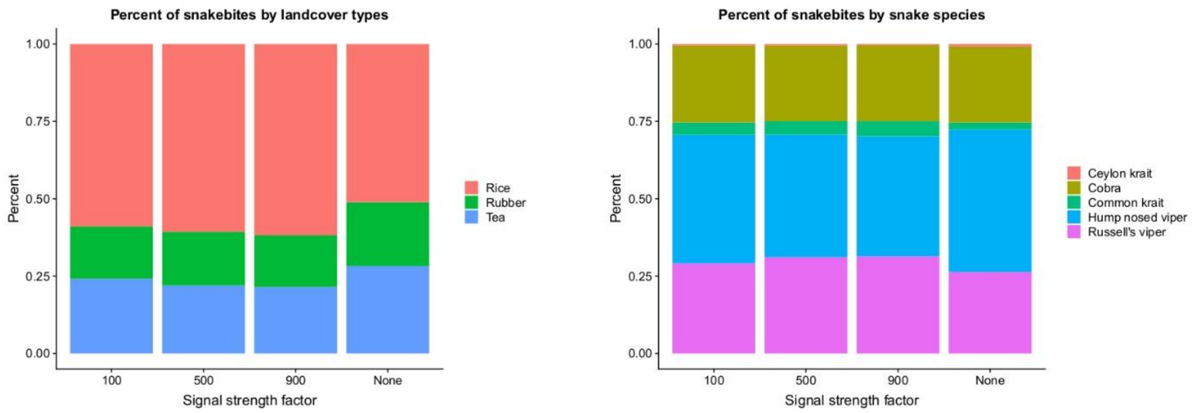

Figure 13: Our simulation was executed 30 times for each signal strength at each one of the locations modelled according to the following possibilities: (x + precipitation)/(x + $\text{precipitation}_{max}$), with x = 100, 500, 900. Changing the signal strength had only a small effect on the percentage of snakebites occurring in each landcover type, and only minor effects on the percentage of bites caused by each on the of the snake species.

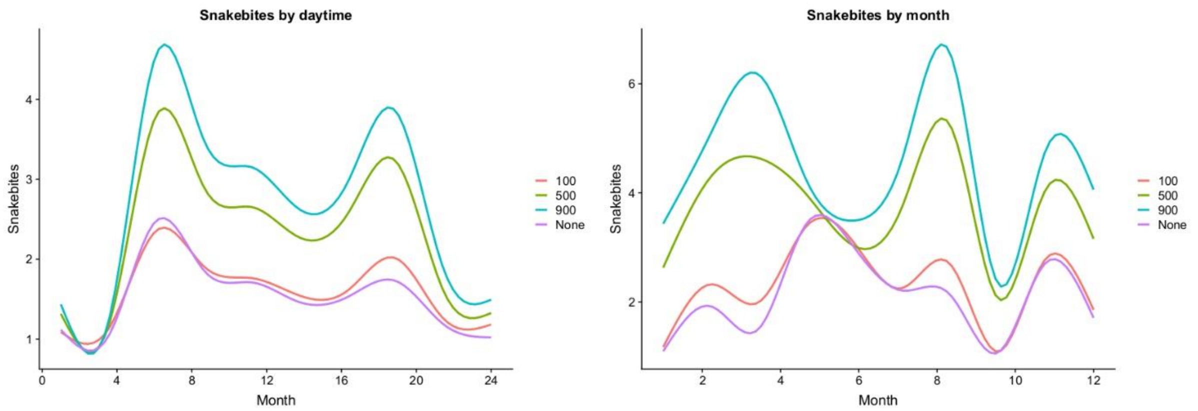

Figure 14: Changing the signal strength had an effect both on the daily distribution of snakebites and on the monthly distribution of snakebites. With a weak and medium signal, the snakes were mostly active regardless of precipitation, so snakebite patterns tended to follow the working patterns of the farmers, while with the strong and linear signals snakes were only active when precipitation was high, and in these two cases snakebite patterns tended to be more influenced by snake activity.

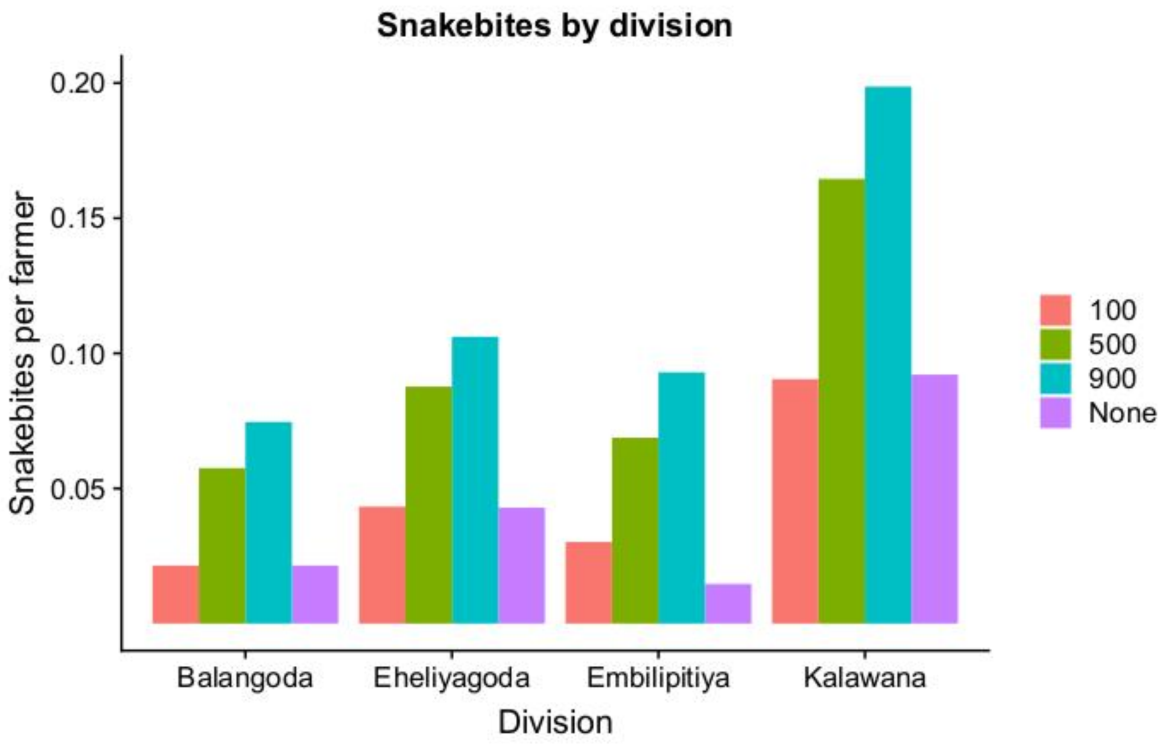

Figure 15: changing the signal strength tended to effect different regions in similar ways across different divisions.

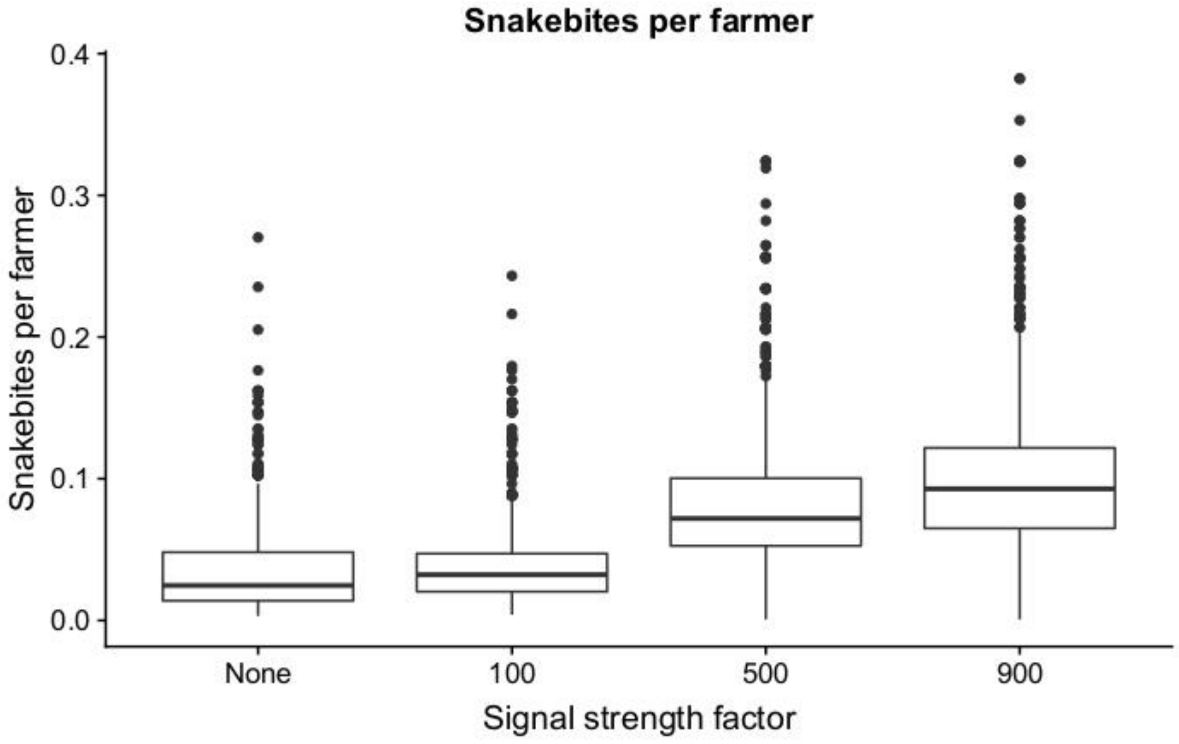

Figure 16: Changing the signal strength factor influenced the total number of snakebites both on the mean and the variance of snakebites per farmer.

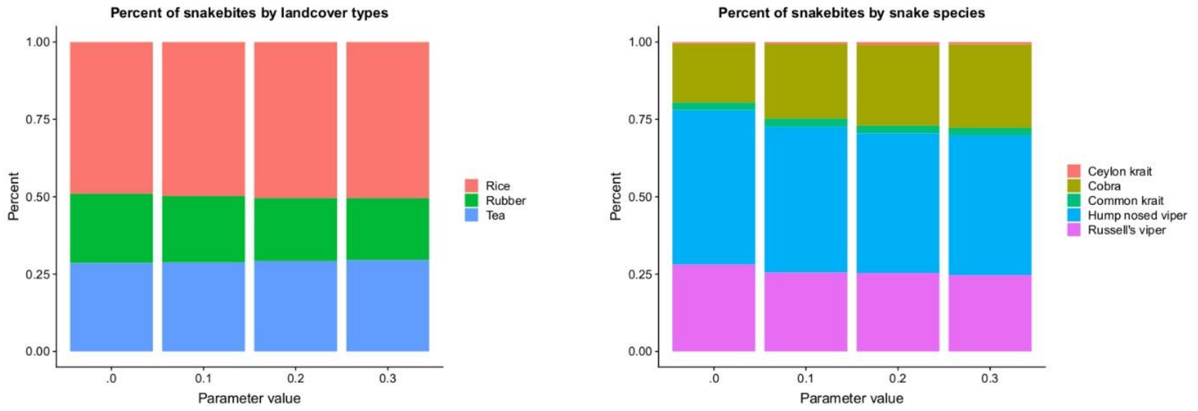

Figure 17: Our simulation was executed 30 times for each activity baseline at each one of the locations modelled with the following possibilities for baseline activity: p = 0, 0.1, 0.2 and 0.3 for all species except for cathemeral snakes, which were kept at a probability of 0.1 across all times of day. Changing the baseline activity probability had only a small effect on the percentage of snakebites on each landcover type but had some effect on the percentage of bites caused by each one of the snake species. A lower baseline probability tended to result in a higher proportion of Russell's viper bites, while a higher baseline level increased the proportion of bites caused by Cobras.

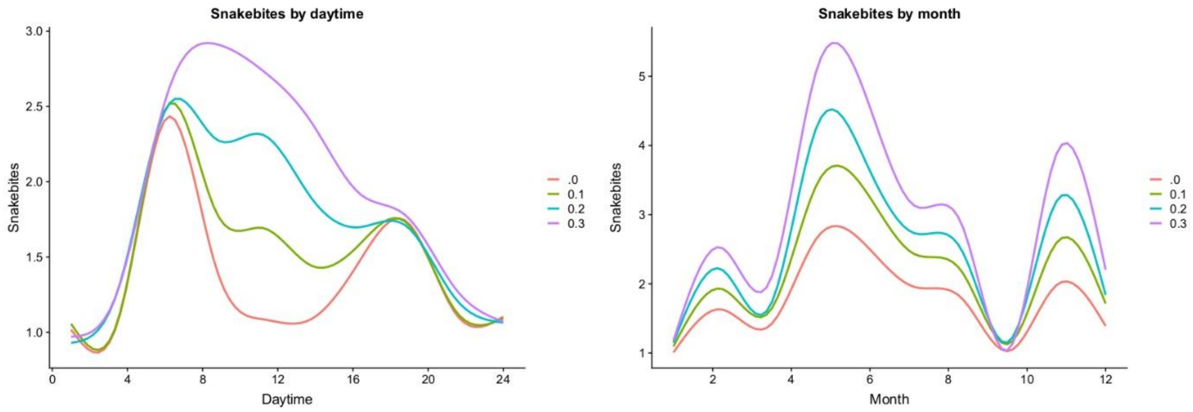

Figure 18: Changing the baseline activity probability had a very strong effect on the distribution of snakebites across the day. A high baseline probability caused a shift of snakebite peak later into the day. Lower baseline activity levels tended to generate a bimodal peak pattern with one large peak in the morning and a second large peak at late afternoon. Changing baseline activity caused a change in magnitude for the yearly distribution of snakebites, but not in pattern.

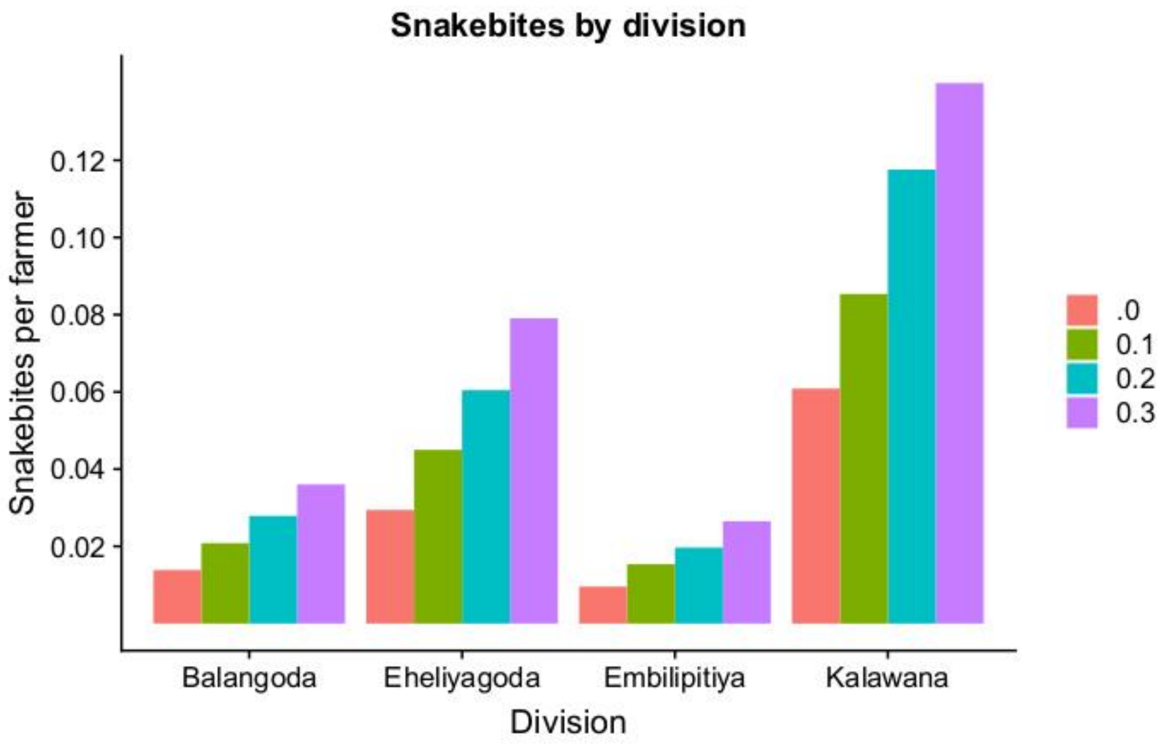

Figure 19: Different baseline probabilities tended to effect different divisions in similar ways. An increase in baseline probability had the same effect regarding the relative number of snakebites across all divisions that we modelled.

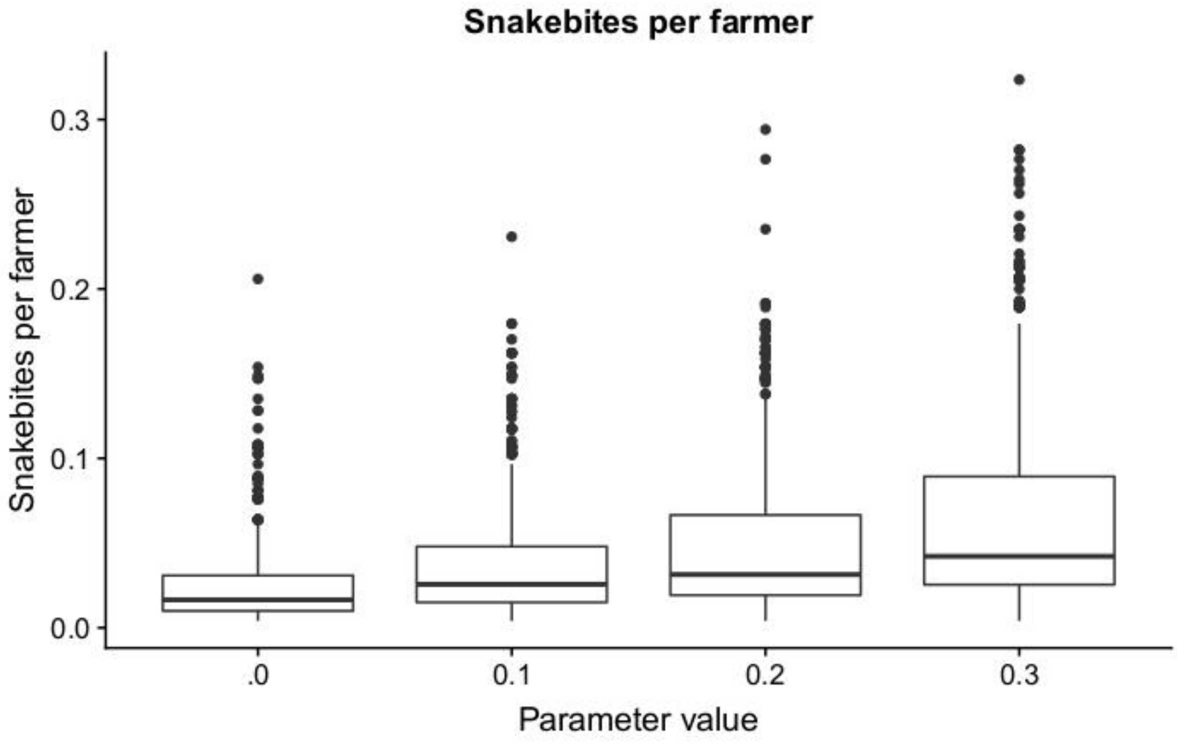

Figure 20: The different baseline probabilities showed that an increase in value would cause an increase in total number of snakebites. The distribution of snakebites around the mean also changed, with a larger variance for higher baseline probability levels, meaning that the uncertainty levels were increase as well.

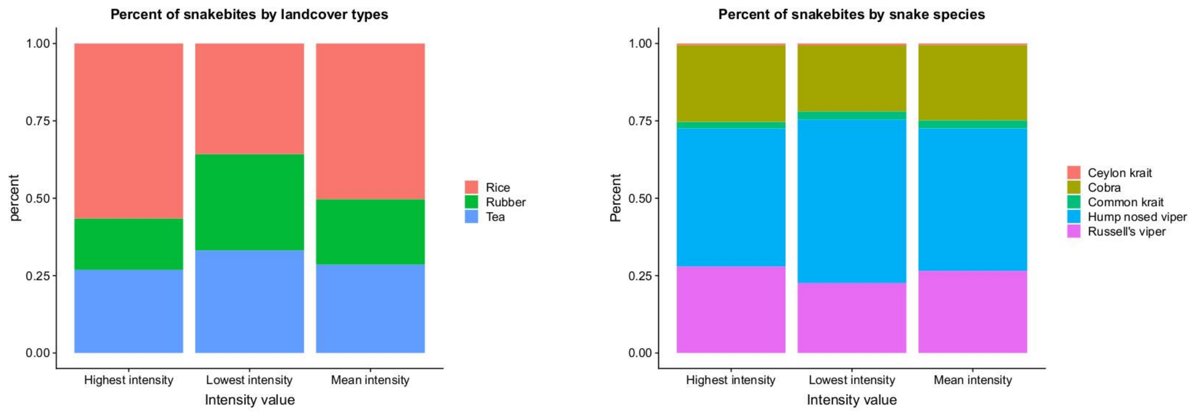

Figure 21: Our simulation was executed 30 times for each intensity value at each one of the locations modelled, where we checked the lowest and highest index values in addition to the mean value. Changing the index influenced the percentage of bites that occurred on the different landcover types. The higher index values that were used caused more snakebites on the rice landcover. Rice had the largest difference between the lowest index value and the highest index values, and this was most likely driving the variation in output. The species of snakes causing snakebites only showed a moderate variation between index values.

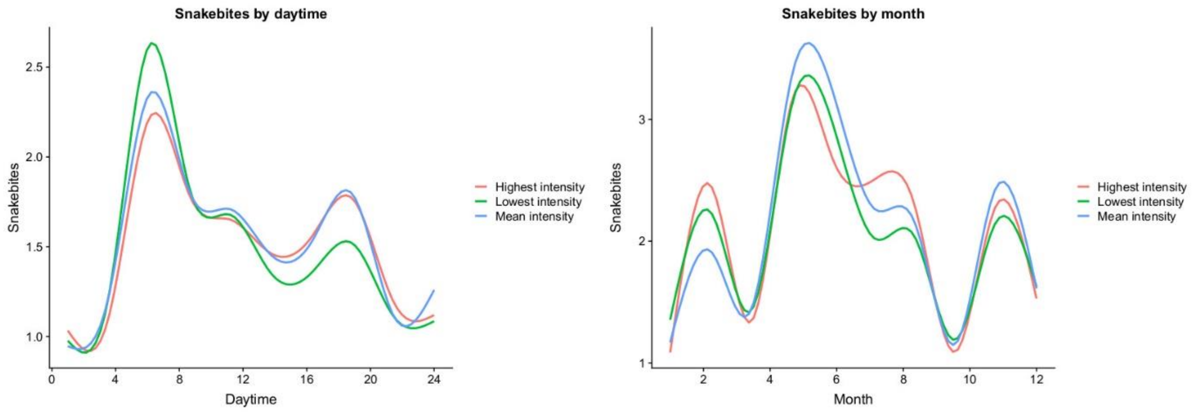

Figure 22: Changing the index value had almost no effect on the patterns of snakebites both on the daily level and a moderate effect on monthly level, with minor differences in the August peak between the different index values.

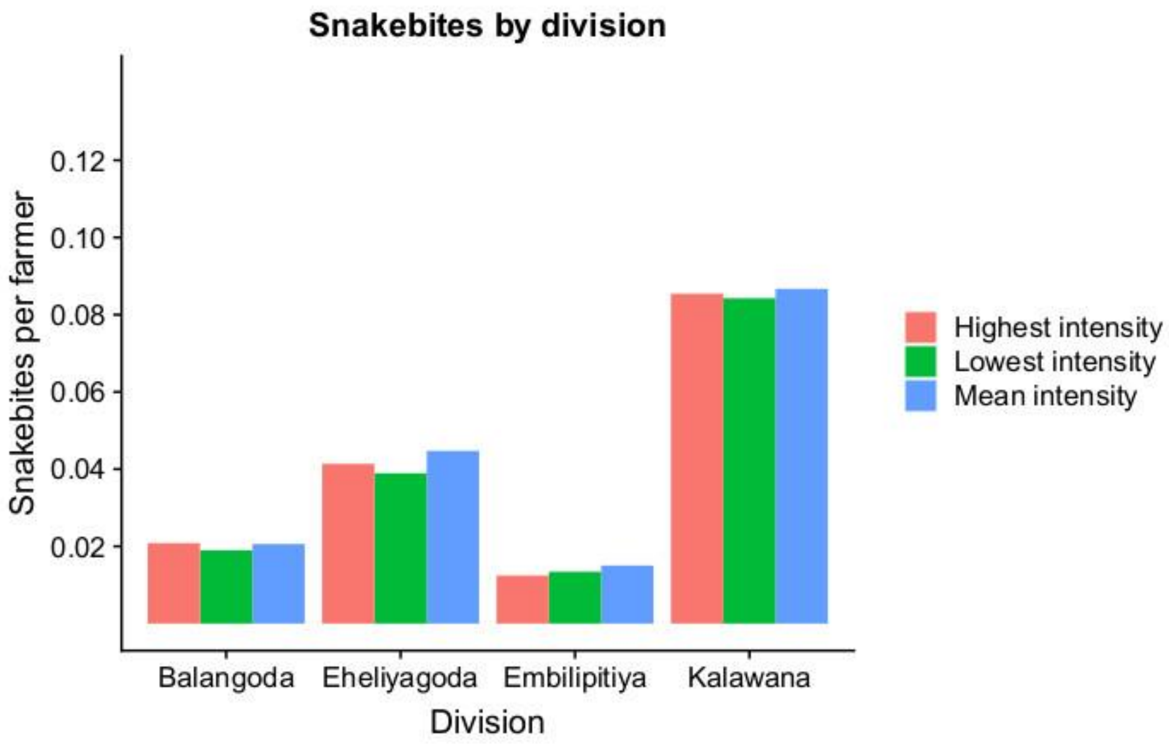

Figure 23: Changing the work index values had only small differences in the total number of snakebites on the division level.

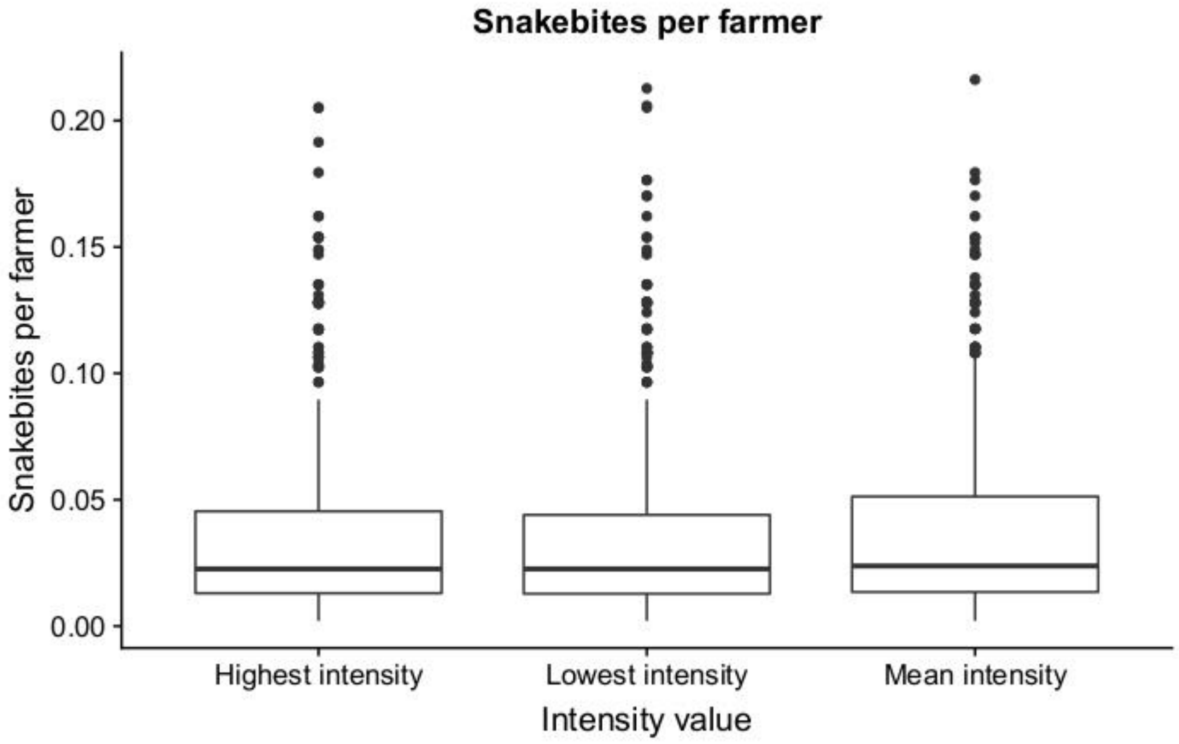

Figure 24: Changing the work index values had not difference in the total number of snakebites. In this output measurement our system showed robustness to the variation in values that we collected during our field work.

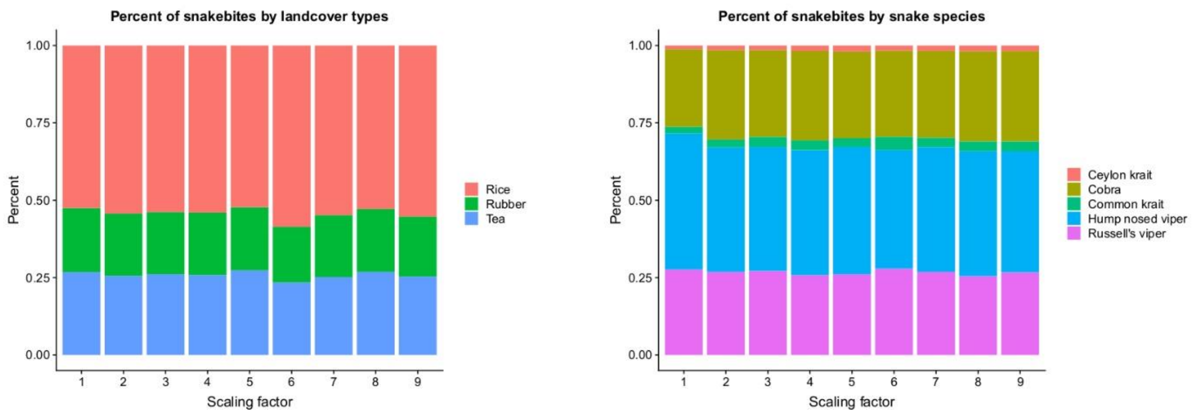

Figure 25: Our simulation was executed 30 times for each population factor at each one of the locations modelled, where changed the factor that was used for scaling up the PPM models by values that ranged between $1x10^{10}$ and $9x10^{10}$. Different population sizes had only a minor effect on either the percentage of snakebites on each landcover type and the percentage of bites caused by each one of the snake species.

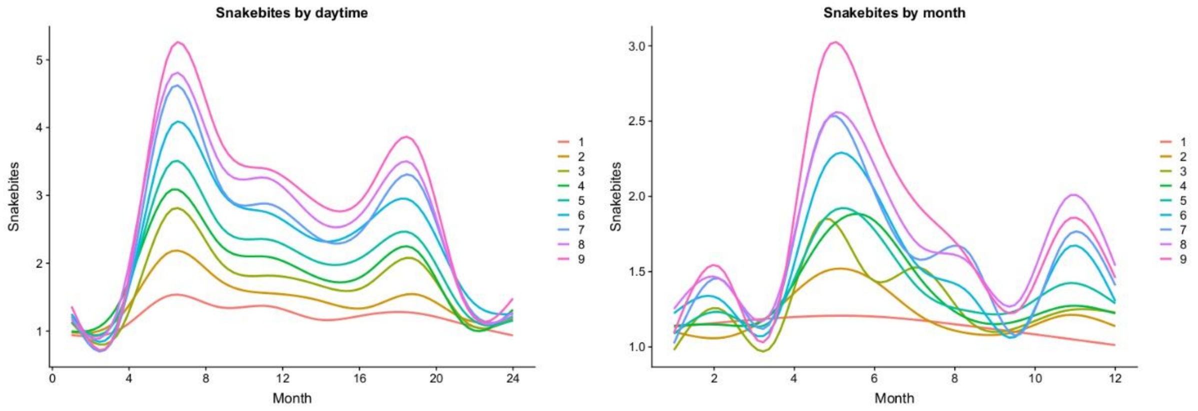

Figure 26: changing the population size had a change in magnitude in the number of snakebites across days, and across months. Below a certain population size the monthly pattern of snakebites stopped showing a distinct yearly pattern where there are distinct major peaks in snakebites, as well as distinct peaks in the daily distribution of snakebites.

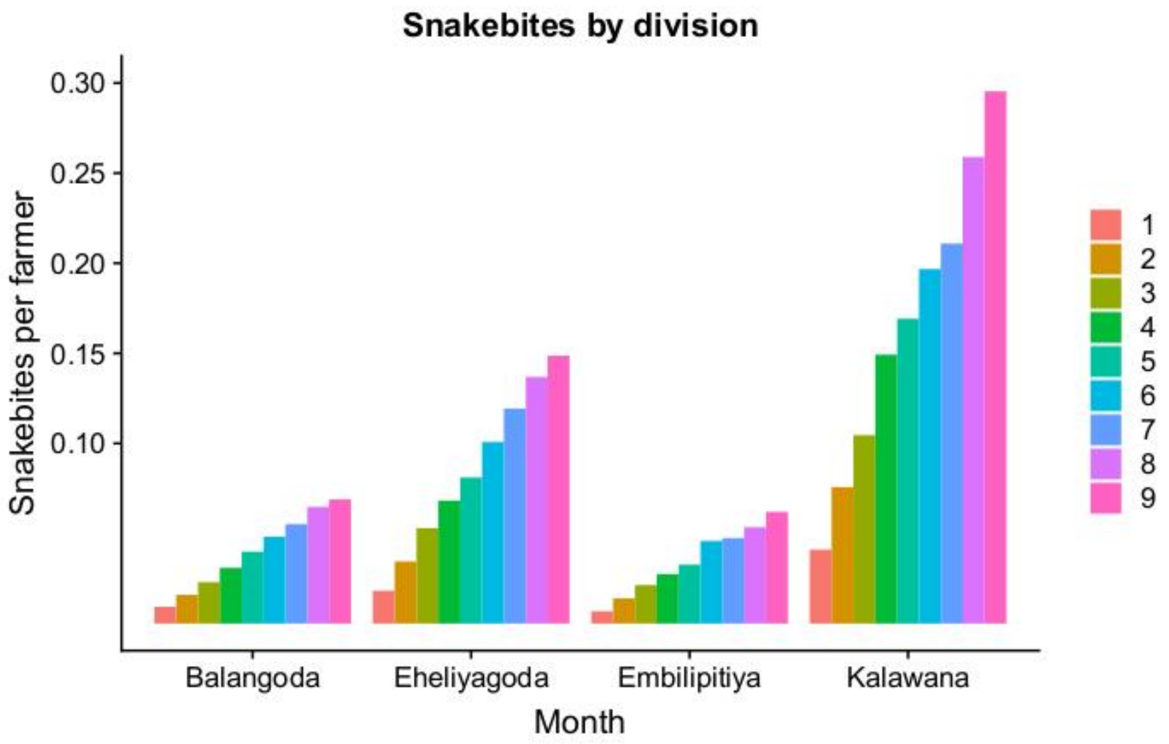

Figure 27: The different population sizes effect the different divisions in distinct ways. While all divisions showed a linear increase in number of snakebites as the snake population was increased, the rate of increase between regions was different.

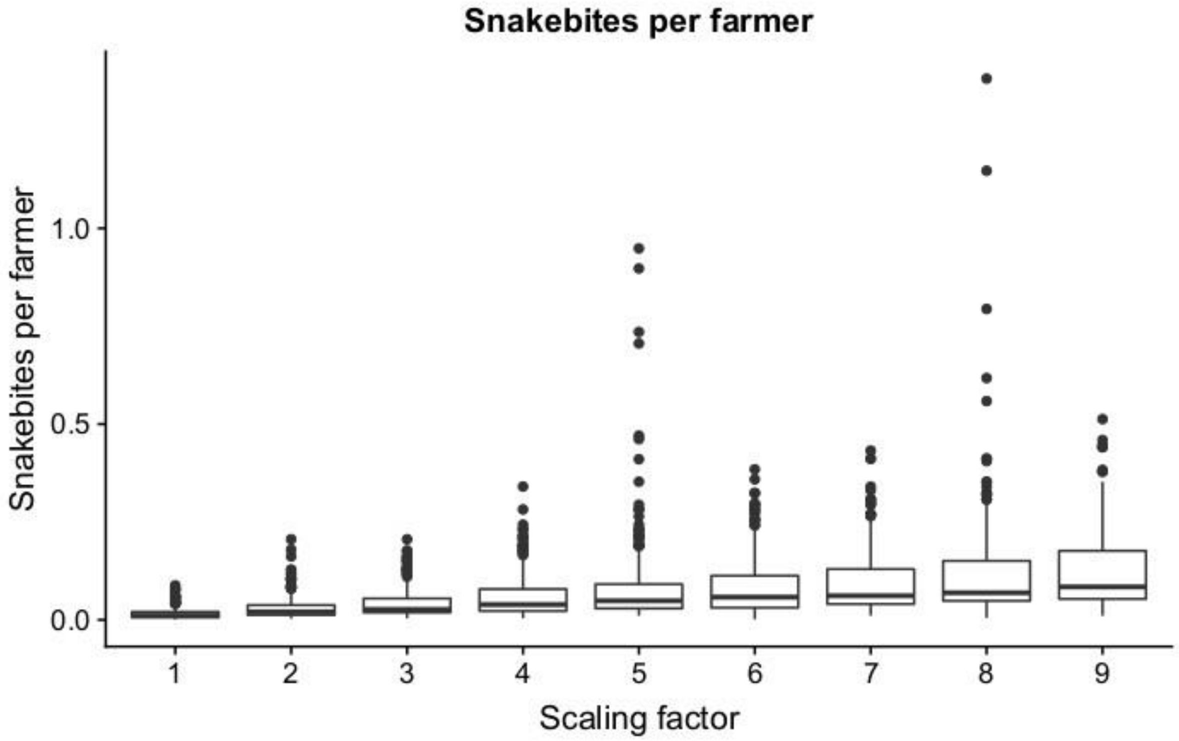

Figure 28: Different population sizes tended to increase the mean number of snakebites, but also increase the variance in number of snakebites per farmer. The larger population sizes had a larger uncertainty level regarding number of bites per farmer.
