# Supplementary material for: Integrating human behavior and snake ecology with agent-based models to predict snakebite in high risk landscapes
Source: PLoS Negl Trop Dis. 2021 Jan 22;15(1):e0009047. doi: 10.1371/journal.pntd.0009047 (PMC7857561; doi:10.1371/journal.pntd.0009047)
Supplement: Attachment — Submitted filename: Review_Goldstein et al.docx [file pntd.0009047.s015.docx]

Review of " Integrating Human Behavior and Snake Ecology with Agent-Based Model to predict Snakebite in High Risk Landscape", by Goldstein *et al.*

This paper proposes and studies a mechanistic model to examine snakebite dynamics by simulating snake-human encounters in rural agricultural communities using agent-based modeling. The paper incorporates snake distributions and behavioral pattern, landscape characteristics and farmers’ behavioral pattern in the model.

This is a well-written paper, and the few missing words never compromise understanding. The paper also appears to be very accurate. Therefore, I recommend the paper be accepted, subject to minor revisions.

The main comment I have is that can the authors scientifically explain their claim that:” When humans and snakes meet on the same cell, a random number is drawn between 0-1, and if it is smaller than the value obtained from the calculation then a snakebite occurs.”

.

Minor points:

1. Pages 7, lines 13 and 30, also page 8, line 30: whenever you are referring to something be more specific. So remove …(see above) and …(see below)
2. Page 8, line 4: the word **to** is missing in the sentence … are able ***to*** work…
3. Page 12, Table 1: some of the sources are not very specific e.g. literature, government’s report etc.
4. Page 17, line 2: the word **on** is missing in the sentence …based **on** simulation…
5. Page 17, Table 3: misspelling of the word ‘intermediate’ in row 6 column 1.
