# Supplementary material for: Integrating human behavior and snake ecology with agent-based models to predict snakebite in high risk landscapes
Source: PLoS Negl Trop Dis. 2021 Jan 22;15(1):e0009047. doi: 10.1371/journal.pntd.0009047 (PMC7857561; doi:10.1371/journal.pntd.0009047)
Supplement: Attachment — Submitted filename: Responses to reviewers.docx [file pntd.0009047.s017.docx]

**Our responses to the comments from reviewers**

**Reviewer #1:**

Methods:

The method is relevant and well explained.

**Thank you.**

Results:

The results are presented according to the analysis plan and clearly exposed.

**Thank you.**

Conclusions:

The limitations of the model are mentioned. The authors clearly show implication of the model and its public health relevance, in Sri Lanka but also in other regions of the world where snakebite envenomation is an issue. The conclusions are consistent and reliable.

**Thank you.**

Editorial and Data Presentation Modifications:

Page 17, 2nd paragraph, line 3: divisions 4 (not divions 4).

**Done (L. 406).**

Summary and General Comments:

Goldstein et al’s manuscript “Integrating human behavior and snake ecology with agent-based models to predict snakebite in high risk landscapes” described an agent based modelling to explain the determinants of man – snake encounter. Results of the study corroborate the observations from several authors and are both convincing and rational.

**Thank you.**

Although it has been hypothesized since a long time that human-snake encounter was influenced by combinations between human activity and snake behavior (in addition to references quoted by the authors: Chippaux et al. Bull Soc Path Exot. 1981; 74 (4): 458-67; Sawai et al. Problem snake management. The Habu and the Brown Treesnake. Cornell University Press, Ithaka, 1999:107-15; Bochner & Struchiner. Cad Saude Publica. 2004;20(4):976-85; Chippaux. Snake venoms, Envenomations. Krieger Publishing Co, Malabar, 2006; Stock et al. Nat Biotechnol. 2007; 25 (2): 173-7; Chippaux. Med Sci (Paris). 2009;25(10):858-62), it had never been proposed a model –particularly an agent based modeling– to predict its impact and define a preventive strategy.

**Thank you, and we appreciate the additional references – we added Stock et al. 2007 (L. 73)**

I am not an expert in modeling and I cannot give a relevant opinion on this topic. However, on the one hand the approach is perfectly appropriate and justified and, on the other hand, the variables considered to build the model seem adequate.

**Thank you.**

As pointed by the authors, some determinants concerning snake population and behavior should be considered in more sophisticated models. For example, several studies have shown that during the mating period, males were encountered 5 to 10 times more than in other seasons of the year and, at the same moment, than females (see Wang et al. Zool Stud. 2003;42(2):379-85; Chippaux. Med Sci (Paris). 2009;25(10):858-62; Bauwens & Claus. Ecol Evol. 2019;9(10):5821-34), which proportionately increases the risk of snakebites. However, it is not certain that such improvements lead to significantly better predictions.

**Thank you. We agree with the reviewer that our model can be improved with such a direction. We added a sentence to further expand along the line of this comments as “For example, it is known that reproductive behavior can increase the chances of encountering snakes [68,69], and integrating this behavior into the model may improve predictions.” (L. 561-563).**

For more detailed analysis of the relationship between human activities and snake behavior, the authors should refer to the chapter by Sawai et al. (in Problem snake management. The Habu and the Brown Treesnake. Cornell University Press, Ithaka, 1999:107-15).

**Thank you for the reference.**

**Reviewer #2 (The review comments of the reviewer 2 are copied from the attached files):**

This paper proposes and studies a mechanistic model to examine snakebite dynamics by simulating snake-human encounters in rural agricultural communities using agent-based modeling. The paper incorporates snake distributions and behavioral pattern, landscape characteristics and farmers’ behavioral pattern in the model.

This is a well-written paper, and the few missing words never compromise understanding. The paper also appears to be very accurate. Therefore, I recommend the paper be accepted, subject to minor revisions.

**Thank you.**

The main comment I have is that can the authors scientifically explain their claim that:” When humans and snakes meet on the same cell, a random number is drawn between 0-1, and if it is smaller than the value obtained from the calculation then a snakebite occurs.”

**We appreciate this comment that point out the area we left not well explained. We have based this section of the model on the assumption that the probability of snakebite incidents can be decomposed into the aggressiveness of each snake and the probability of snake presence for each species. Drawing a number randomly against for a probability threshold is a standard method in simulation to represent such probabilistic events. By computing this for every single cell for every single time step, eventual frequency will follow statistical distribution based on snake biology.**

**We have now added the following to this section of the paper to clarify the biological implication “This function is designed in order to assign a threshold for bites according to each snakes agressivness level, with the assumption that combining the agressivness along with the human-snake overlap would provide a good measure for snakebites occurring [53]” (L.343-345)**

Minor points:

Pages 7, lines 13 and 30, also page 8, line 30: whenever you are referring to something be more specific. So remove …(see above) and …(see below)

**Done (L.149, L.166, L.173).**

Page 8, line 4: the word **to** is missing in the sentence … are able ***to*** work…

**Done (L.174).**

Page 12, Table 1: some of the sources are not very specific e.g. literature, government’s report etc.

**We’ve now added specific citations.**

Page 17, line 2: the word **on** is missing in the sentence …based **on** simulation…

**Done (L. 396).**

Page 17, Table 3: misspelling of the word ‘intermediate’ in row 6 column 1.

**Done (L. 418).**

**Reviewer #3 (including the responses for the PDF file):**

Methods:

The introduction is adequate, objectives are well presented. The study design appropriate.

**Thank you.**

Results:

The data generated are well articulated.

**Thank you.**

Conclusions:

The conclusion needs editing to relate the finding of the study to real time realities in the study sites.

**We appreciate this comment. While we see the benefit to summarize the local implication in the conclusion section, we also would like to expand general implication of our approach. Thus we modified the last three paragraphs, the two paragraphs which subsequently lead to the final conclusive paragraph, and the conclusion paragraphs to include the insights from our approach for the study regions. We modified the third last paragraph as “Our study clearly showed that the spatio-temporal synchronicity in both snake and farmer behaviors is the key to understand the snakebite patterns in the Ratnapura district in Sri Lanka (Figure 6 & SI 1 Figure 2). In particular, difference in climatic profiles in the district may results complex snake-farmer associations evident from the snakebites patterns as well as the composition of responsible snake species (Figure 4-5 & 7). While our study shows a strong implication of social-ecological dynamisms of snakebites in dry and wet-dry climate zones in Sri Lanka, other studies have already invoked similar mechanisms to explain observed patterns of risk in rural communities outside of Sri Lanka (e.g.: [14,17])…..” (L. 537-549)**

**Also in the second last paragraph: “...and integrating these traits may reduce the overestimation of cobra bites in comparison to other snake species and improve our predictions for the Ratnapura region” (L565-566)**

**As well as in the summary “With snakebite burden in Sri Lanka expected to increase under climate change [7] our findings carry important implications for future snakebite prevention in the study sites where it was developed.” (.588-590)**

Summary and General Comments:

The study is novel and well presented.

**Thank you.**

Comments on the PDF file (PNTD-D20-01368_reviewed.pdf):

L.79-80: use the right reference format.

**Done. We changed the format, and it now reads: “For example, Yañez-arenas et al., (2016) [19] show a correlation between snake distributions and bites, and Akani et al., (2013) [14] ...”.**

L.912: changes.

**kept as ‘change’ because land use and cover change (LUCC) is commonly used is scientific literature**

L.133: University

**Done.**

L.136 recast: repetition

**The section now reads “****Agent based modelling (ABM) is a bottom up approach for modeling complex and adaptive systems using autonomous agents, that explain macro level phenomenon [19–22]. ABM is used both for studying complexity that is not easily reducible to differential equations, and discovering emergent patterns and phenomenon found in those systems, as well as study the internal dynamics of these system [23,24].” (L.136-141)**

L.143: what is R?

**It is the name of statistical package. Reference added.**

L.147: Repetition

**Left as is, because transmission of zoonotic disease across landscape is a different category of models than land use and land cover change.**

L.167: Delete ‘of time’

**Done.**

L.174: able to work

**Done.**

L.181: What about feeding habit? This is a very important that influences the behavioral traits of snakes?

**Agreed. In the paragraph stating the current limitation of the model, we have mentioned the importance of integrating feeding habits of snakes. It reads “While our model represented some of the most important snake behavior factors relevant to snakebite, there are other elements that we did not address, primarily due to data limitations. These include reproduction phenology and its association with climate [4], seasonal variability in landcover preferences [59], or feeding habits and species-specific feeding strategies.” (L.558-561)**

L.256: what about distance of house to farmer-snake?

**Agreed and we have indeed collected relevant information during our fieldwork. In the end we didn’t incorporate this factor, as in this paper we focused on the landcover types and associated farmers behaviors, and the interactions with these factors and snake biology. Our dataset is not strong enough to be able to include the incidents during the commute into our simulation model, which is already complicated. We mentioned the potential implications of this aspects in the Discussion section. Now reads “Additionally, we did not integrate the distance between homes and fields and snakes due to data limitations, even though it has been known to be an important factor for snakebite occurrence.” (L. 571-573).**

L.286: Poisson Point Process Model

**Done.**

L.341 Recast for clarity

**The section now reads “Agents are tracked within the model locations and their encounters (occurring in the same grid cell at the same time) are recorded as snakebites under the following conditions.” (L.331-333).**

L.396: based on

**Done.**

Figure 4: What percent?

**changed to ‘Percent of snakebite’**

L.414: insert the locations here

**Done. Now reads “both in divisions 1-3 (Eheliyagoda, Balangoda, and Kalawana)” (L. 404-405).**

L.418 (Table 3): is this not underestimated? (The reviewer mentions the estimated composition of Russel’s viper and Hump nose viper in the model prediction).

**Agreed that compared with the hospital records, the simulated contribution percentage of Russel’s viper and Hump nose viper against entire snakebite incidents are lower in intermediate zone. However, we would like to point out that our simulation correctly captured the order of significance among species. It is possible that a few species specific mechanisms contribute such discrepancy. We have now stated the discrepancy in the results, which reads “The simulation contribution of Cobras was overestimated in all locations, and the simulated contribution of Russell’s viper and hump nosed viper against entire snakebites were underestimated in the intermediate zone.” (L. 406-409). We also discussed the potential reason in the Discussion as “Additionally, differences between feeding strategies such as active hunting (e.g.N*naja naja*) and ambush (e.g. *Hypnale hypnale* & *Daboia russellii*) may lead to different encounter outcomes, and integrating these traits may reduce the overestimation of cobra bites in comparison to other snake species and improve our predictions for the Ratnapura district. ” (L. 563-566).**

L.477: within these locations?

**Edited as suggested.**

L. 553 “Further afield, other studies have similarly reported land-use specific risks (e.g. rubber in Liberia and rice in the Philippines) [63,64].”: ???

**We understand that the reviewer pointed out “Further afield,” is hard to understand. Now reads “Outside of Sri Lanka,”**

L.593: it is important to relate the results of your model with the reality on the ground

**See comment above from the “conclusion” section of the Reviewer #3**

L.606: Journal

**Changed to “J Venom Anim Toxins Incl Trop Dis.** ”

L.719 (on a reference "Ministry of Plantation Industries Sri Lanka. Statistical Information on Plantation Crops. 2013".): page numbers

**Page numbers are not included because we find the entire report is relevant for regional patterns of tea and rubber, including statistics for small holders and large plantations in the different districts, therefore we prefer not to include page numbers.**
